# Supplementary material for: Identification of Paired-related Homeobox Protein 1 as a key mesenchymal transcription factor in pulmonary fibrosis
Source: eLife. 2023 Jun 1;12:e79840. doi: 10.7554/eLife.79840 (PMC10275639; doi:10.7554/eLife.79840)

Blots for Figure 1C

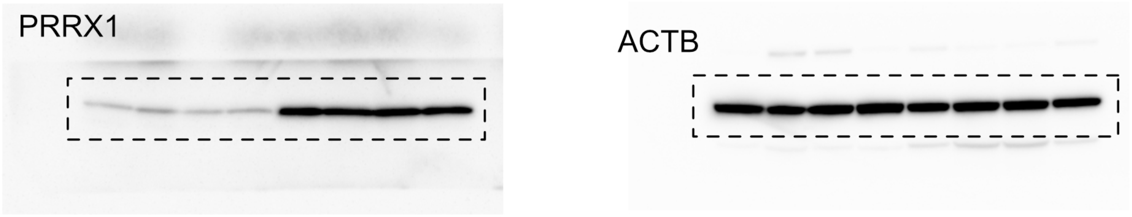

Blots for Figure 2D

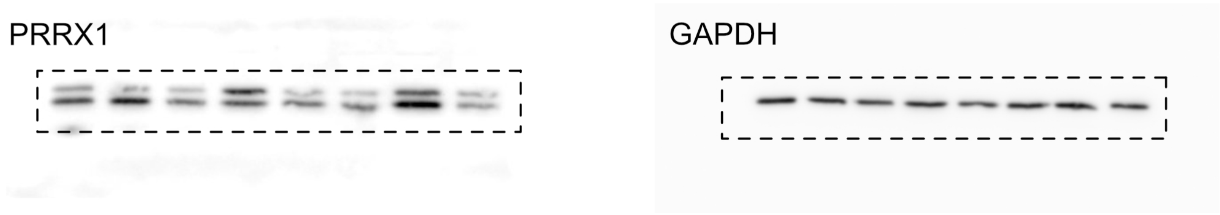

Blots for Figure 4B

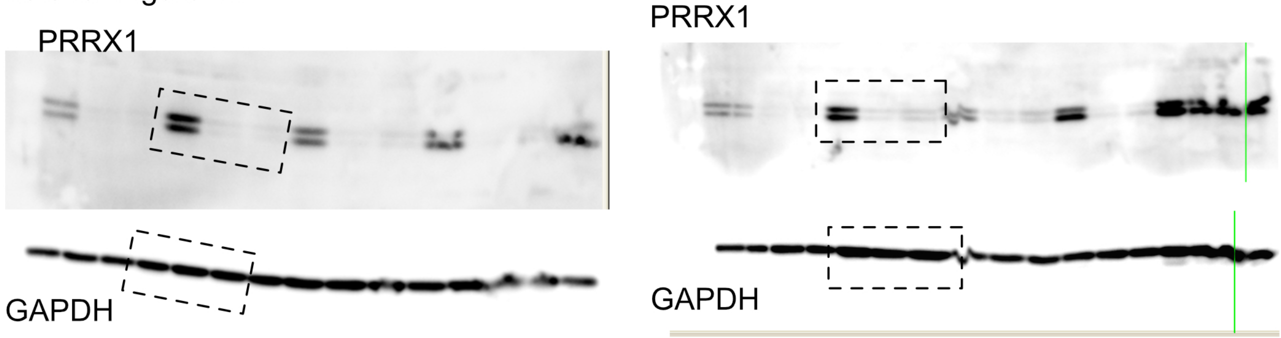

Blots for Figure 5B

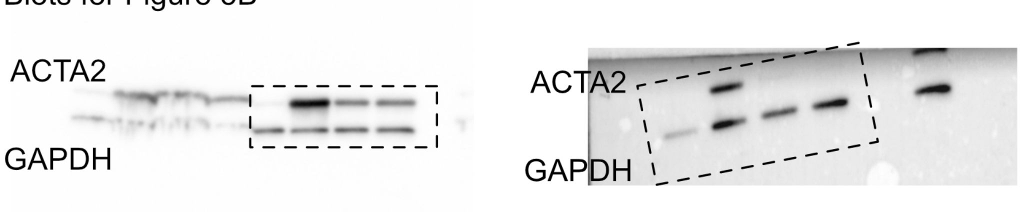

Blots for Figure 5E

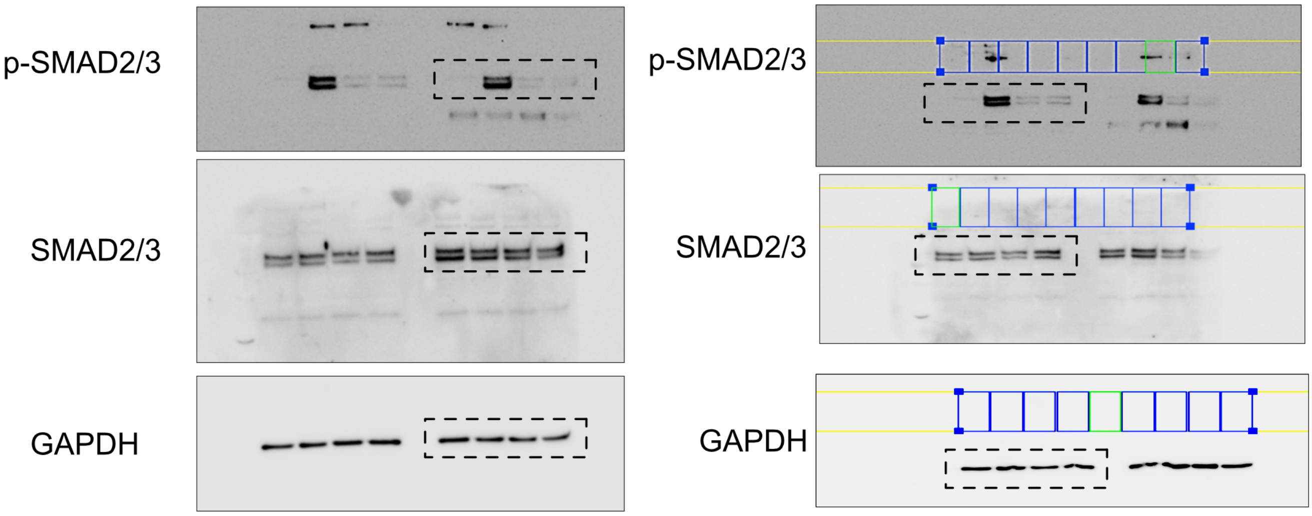

Blots for Figure 6B

PRRX1

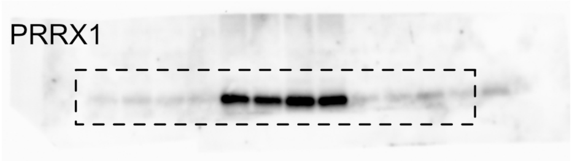

TUB

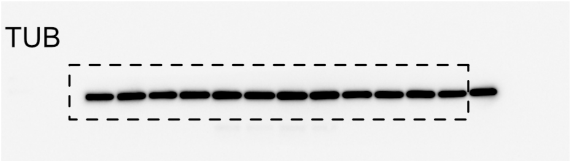

Blots for Figure 7B

FN1

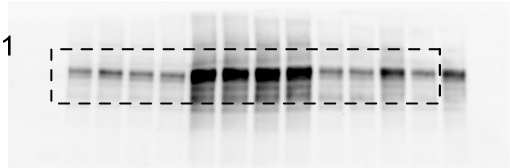

COL1

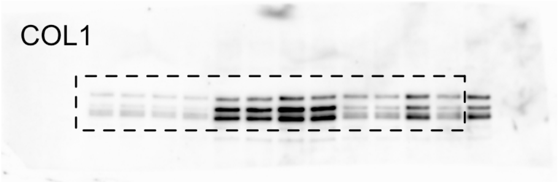

ACTA2

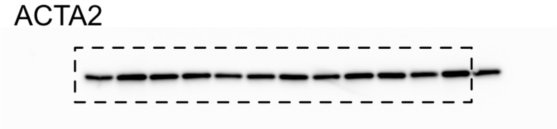

(With respect to TUB, see unedited blots for Figure 6B above - from same blot serie)

Blots for Figure 8B

FN1

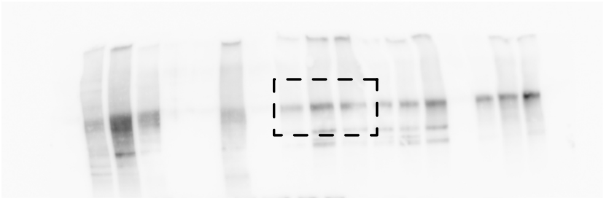

COL1

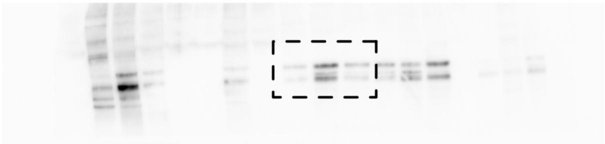

ACTA2

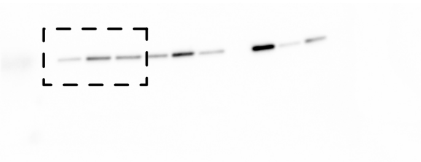

TUB

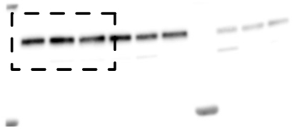

Supplement: Figure 1—source data 1. — Labelled (.pdf) and raw (folder) blot images showed in panel C are also included. [file elife-79840-fig1-data1.zip › Figure 1 - source data/Blot-MainFigures-labelled.pdf]
