# Supplementary figures and images for: Identification of Paired-related Homeobox Protein 1 as a key mesenchymal transcription factor in pulmonary fibrosis
Source: eLife. 2023 Jun 1;12:e79840. doi: 10.7554/eLife.79840 (PMC10275639; doi:10.7554/eLife.79840)

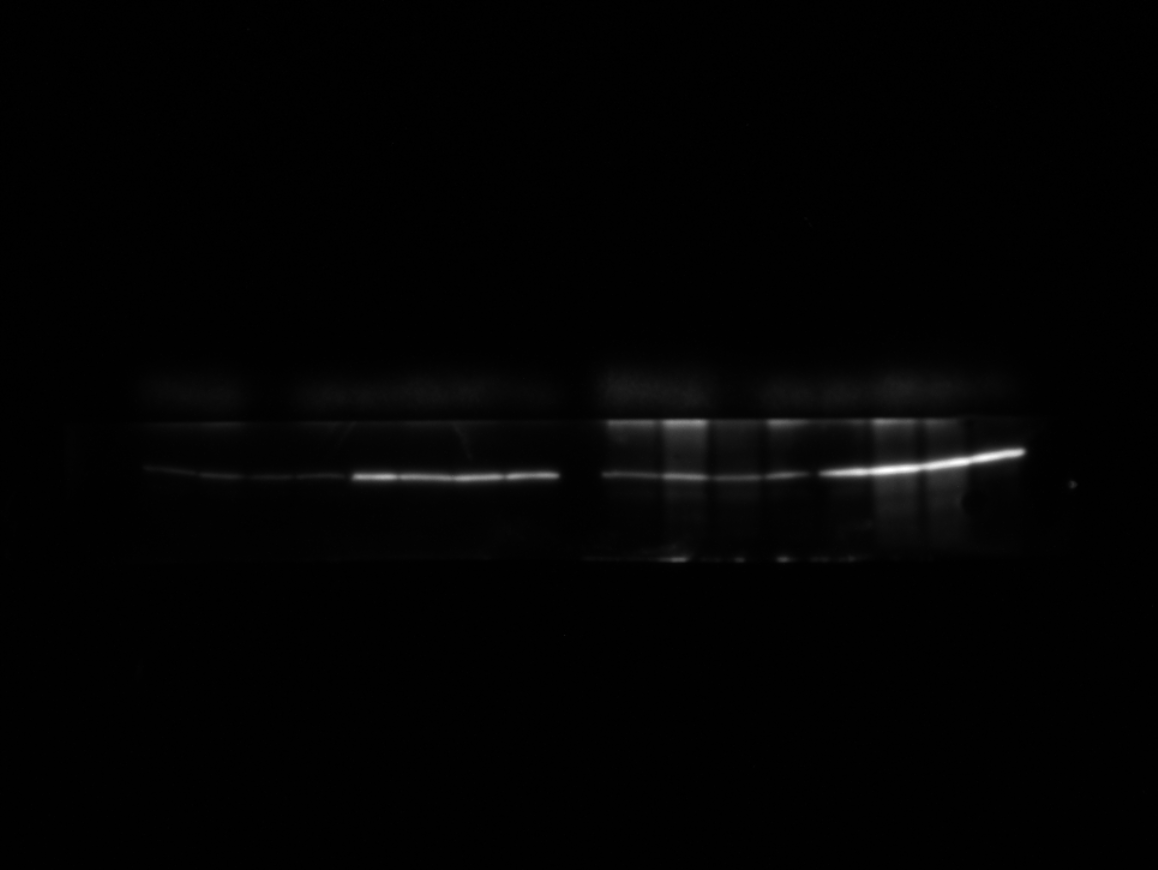

Supplement: Figure 1—source data 1. — Labelled (.pdf) and raw (folder) blot images showed in panel C are also included. [file elife-79840-fig1-data1.zip › Figure 1 - source data/Blot Figure 1C/Lane_PRRX1-1C.tif]

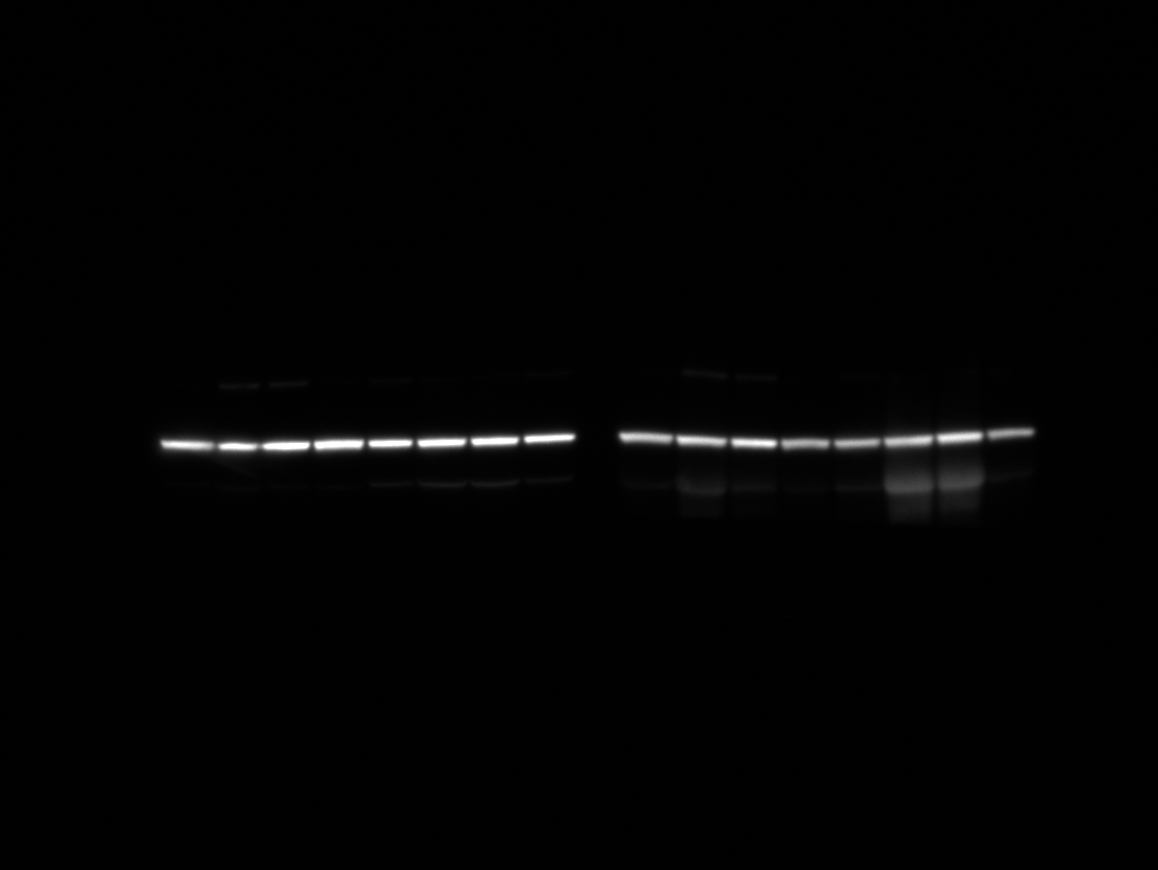

Supplement: Figure 1—source data 1. — Labelled (.pdf) and raw (folder) blot images showed in panel C are also included. [file elife-79840-fig1-data1.zip › Figure 1 - source data/Blot Figure 1C/Lane_ACTB-1C.tif]

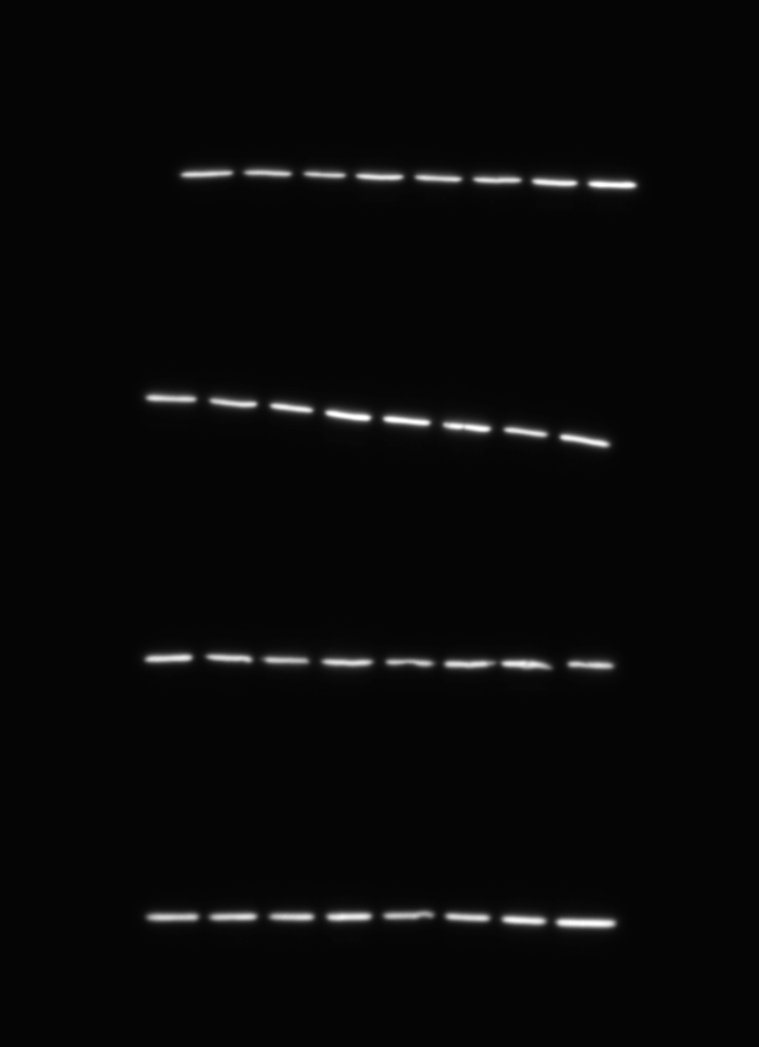

Supplement: Figure 2—source data 1. — Labelled (.pdf) and raw (folder) blot images showed in panel E are also included. [file elife-79840-fig2-data1.zip › Figure 2 - source data/Blot Figure 2E/Lane_TUB-2E.tiff]

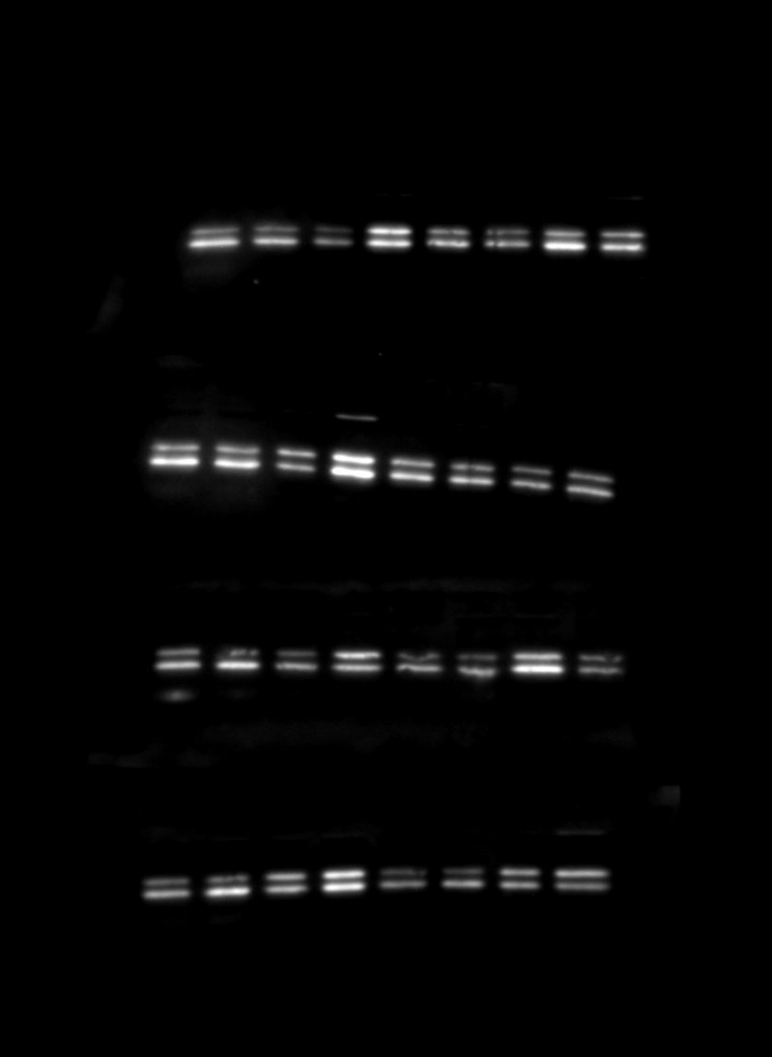

Supplement: Figure 2—source data 1. — Labelled (.pdf) and raw (folder) blot images showed in panel E are also included. [file elife-79840-fig2-data1.zip › Figure 2 - source data/Blot Figure 2E/Lane_PRRX1-2E.tiff]

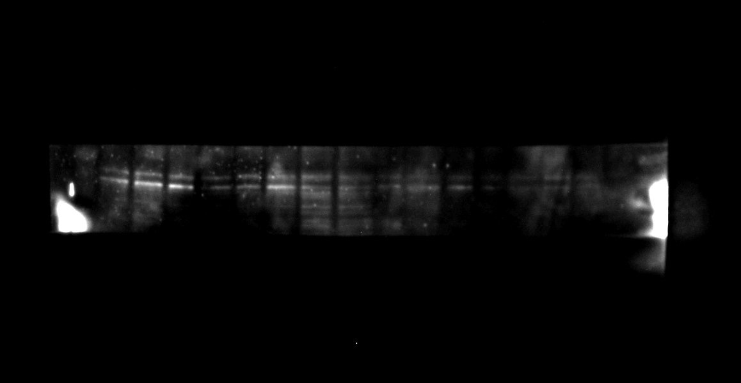

Supplement: Figure 3—figure supplement 1—source data 1. — Labelled (.pdf) and raw (folder) blot images showed in panels A,D, and E are also included. [file elife-79840-fig3-figsupp1-data1.zip › Figure 3-figure supplement 1 - source data/Blot Figure 3 - figure supplement 1/Figure 3-S1D/Lane_PRRX1-Control-3S1D.tiff]

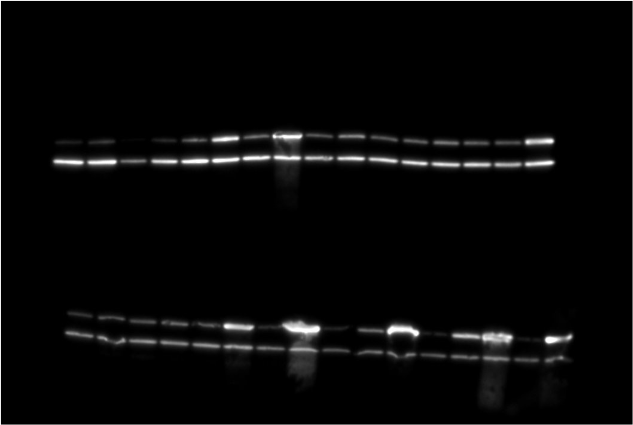

Supplement: Figure 3—figure supplement 1—source data 1. — Labelled (.pdf) and raw (folder) blot images showed in panels A,D, and E are also included. [file elife-79840-fig3-figsupp1-data1.zip › Figure 3-figure supplement 1 - source data/Blot Figure 3 - figure supplement 1/Figure 3-S1D/Lane_GAPDH-IPF-3S1D.tiff]

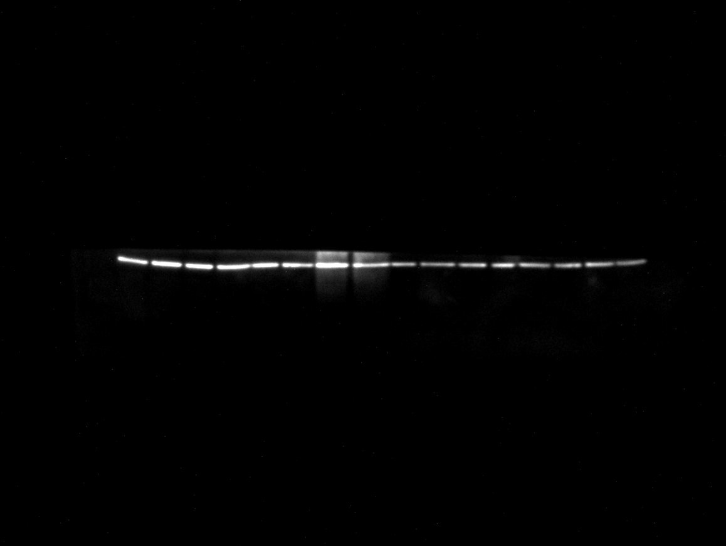

Supplement: Figure 3—figure supplement 1—source data 1. — Labelled (.pdf) and raw (folder) blot images showed in panels A,D, and E are also included. [file elife-79840-fig3-figsupp1-data1.zip › Figure 3-figure supplement 1 - source data/Blot Figure 3 - figure supplement 1/Figure 3-S1D/Lane_GAPDH-Control-3S1D.tiff]

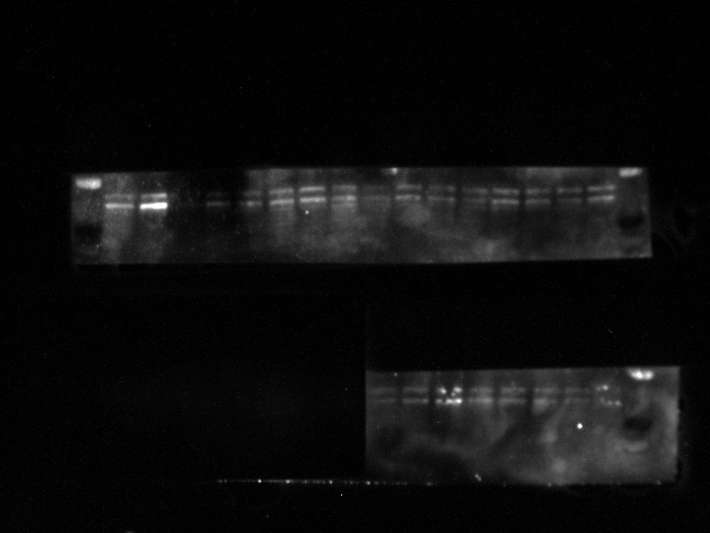

Supplement: Figure 3—figure supplement 1—source data 1. — Labelled (.pdf) and raw (folder) blot images showed in panels A,D, and E are also included. [file elife-79840-fig3-figsupp1-data1.zip › Figure 3-figure supplement 1 - source data/Blot Figure 3 - figure supplement 1/Figure 3-S1D/Lane_PRRX1-IPF-3S1D.tiff]

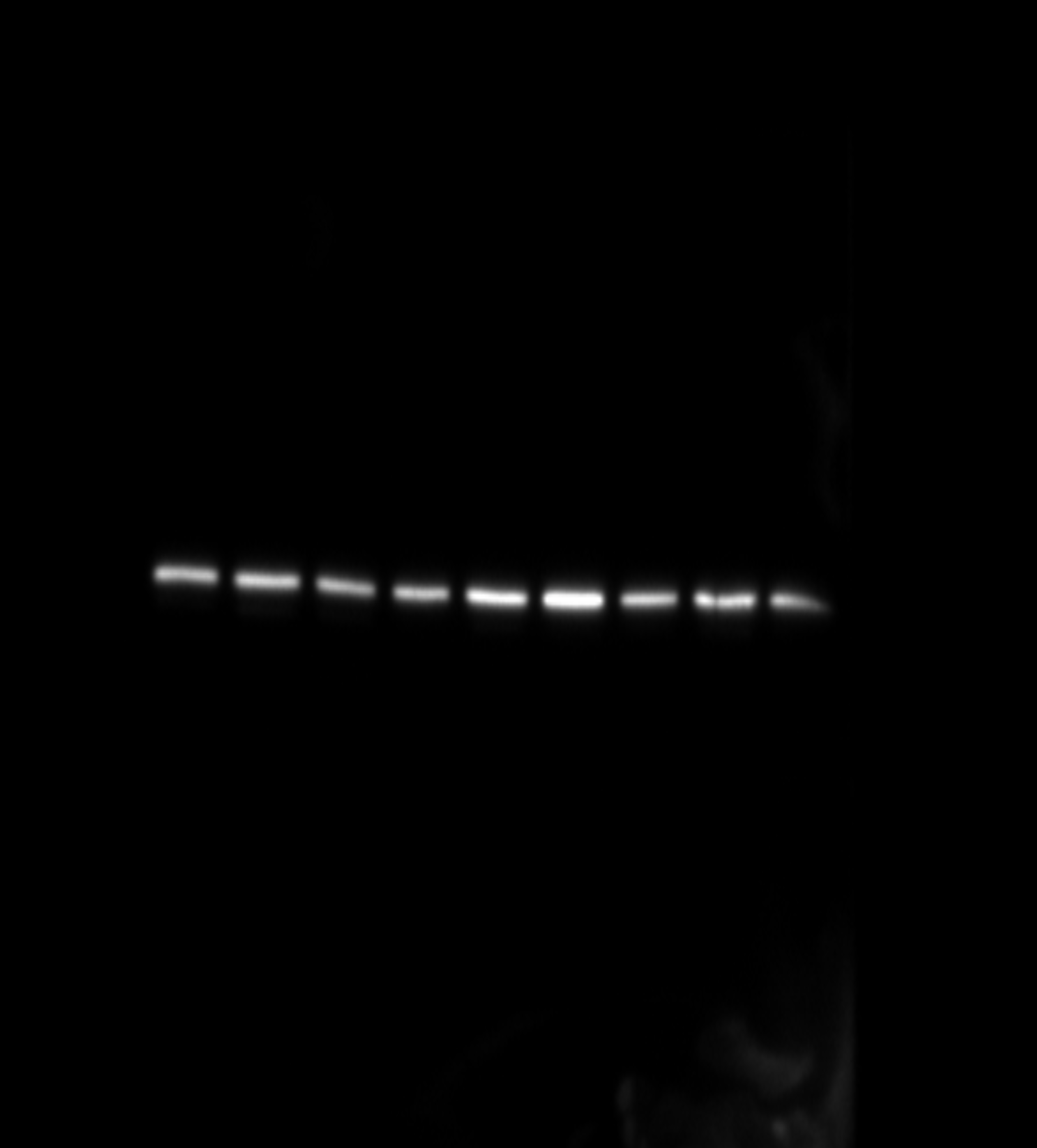

Supplement: Figure 3—figure supplement 1—source data 1. — Labelled (.pdf) and raw (folder) blot images showed in panels A,D, and E are also included. [file elife-79840-fig3-figsupp1-data1.zip › Figure 3-figure supplement 1 - source data/Blot Figure 3 - figure supplement 1/Figure 3-S1E/Lane-IPF-GAPDH-3S1E.tiff]

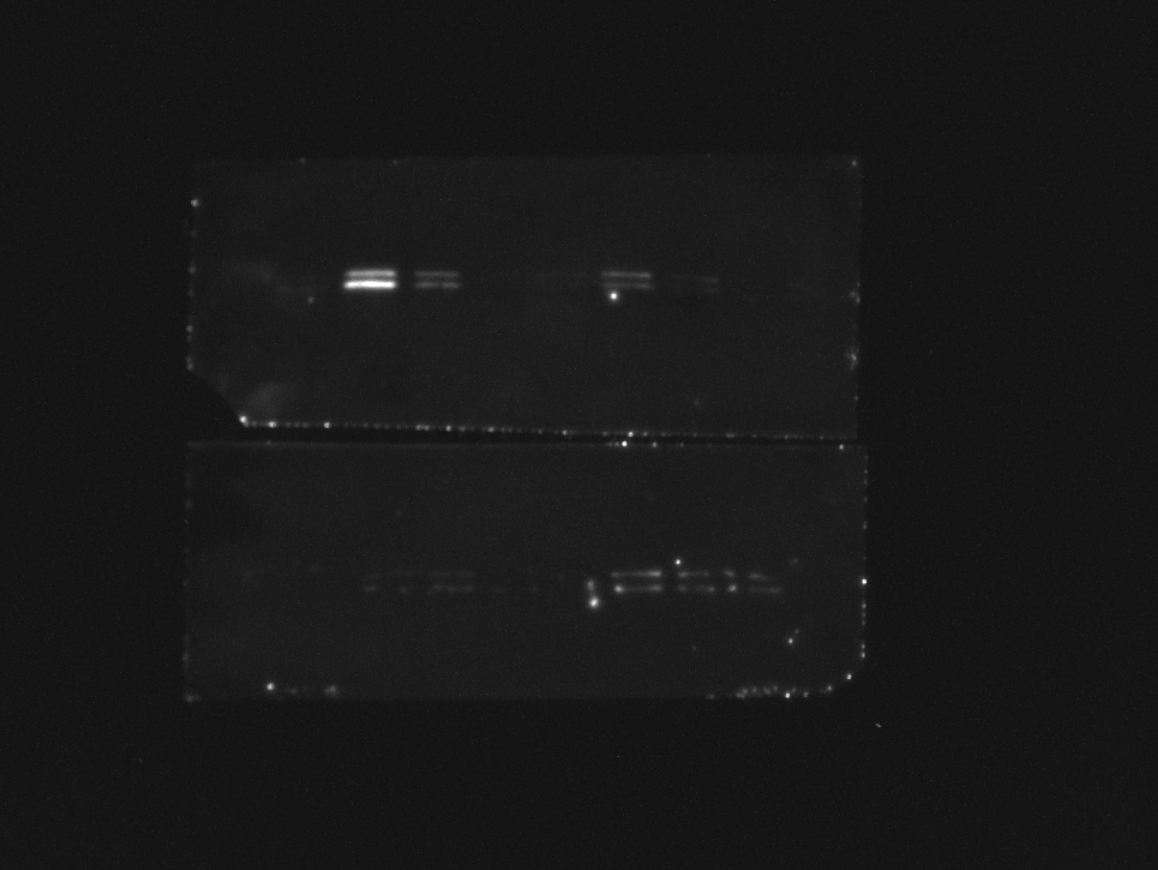

Supplement: Figure 3—figure supplement 1—source data 1. — Labelled (.pdf) and raw (folder) blot images showed in panels A,D, and E are also included. [file elife-79840-fig3-figsupp1-data1.zip › Figure 3-figure supplement 1 - source data/Blot Figure 3 - figure supplement 1/Figure 3-S1E/Lane-control-PRRX1-3S1E.tiff]

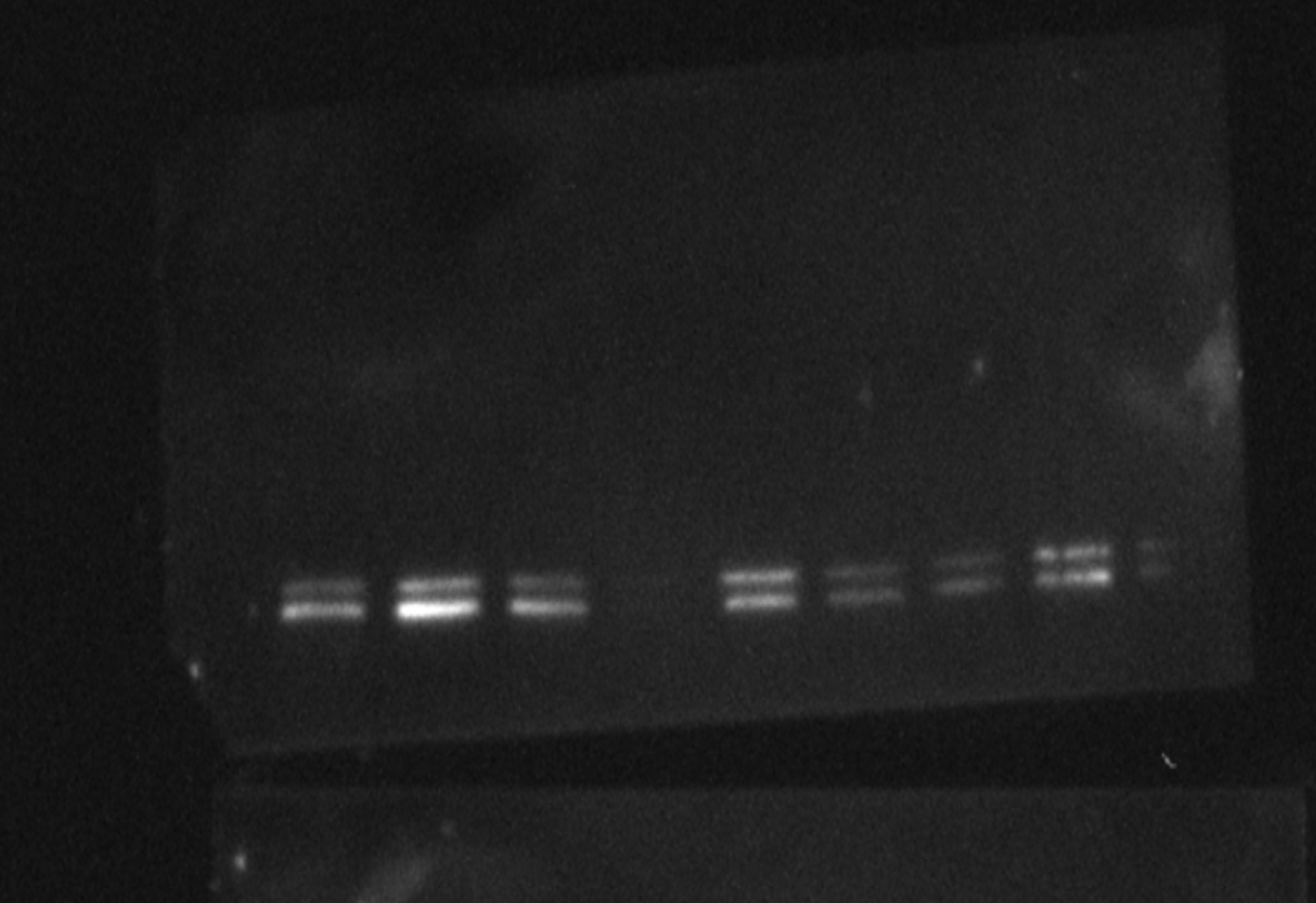

Supplement: Figure 3—figure supplement 1—source data 1. — Labelled (.pdf) and raw (folder) blot images showed in panels A,D, and E are also included. [file elife-79840-fig3-figsupp1-data1.zip › Figure 3-figure supplement 1 - source data/Blot Figure 3 - figure supplement 1/Figure 3-S1E/Lane-IPF-PRRX1-3S1E.tiff]

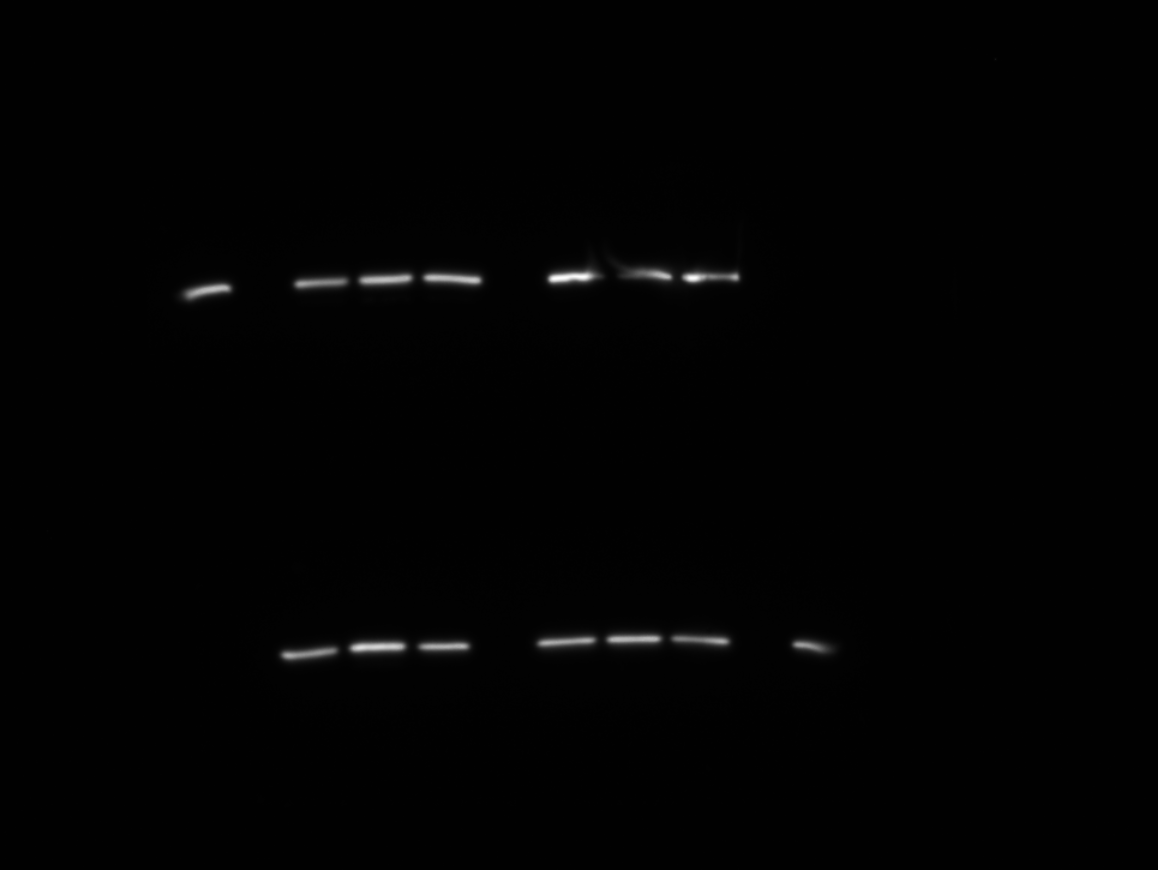

Supplement: Figure 3—figure supplement 1—source data 1. — Labelled (.pdf) and raw (folder) blot images showed in panels A,D, and E are also included. [file elife-79840-fig3-figsupp1-data1.zip › Figure 3-figure supplement 1 - source data/Blot Figure 3 - figure supplement 1/Figure 3-S1E/Lane-control-GAPDH-3S1E.tiff]

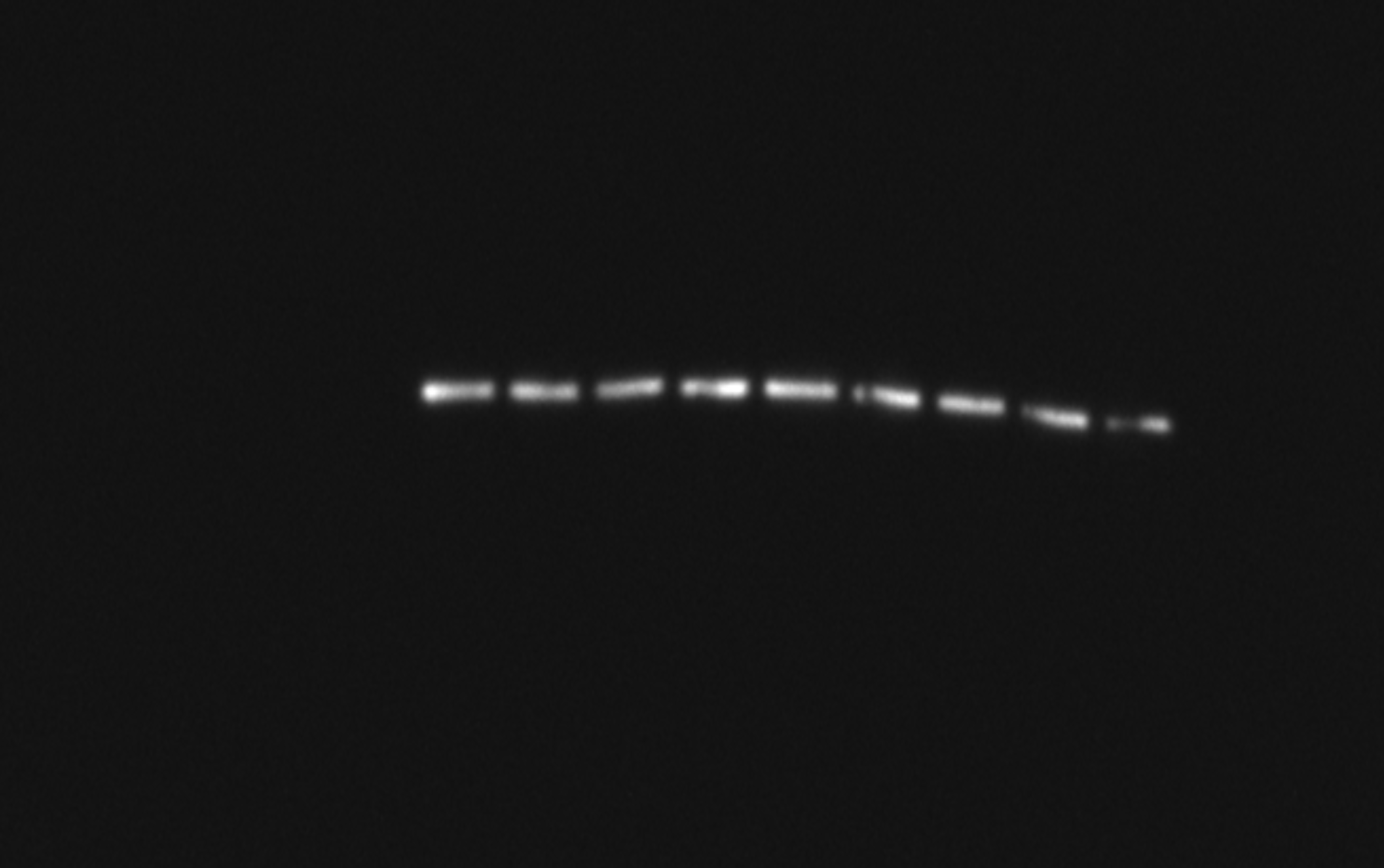

Supplement: Figure 3—figure supplement 1—source data 1. — Labelled (.pdf) and raw (folder) blot images showed in panels A,D, and E are also included. [file elife-79840-fig3-figsupp1-data1.zip › Figure 3-figure supplement 1 - source data/Blot Figure 3 - figure supplement 1/Figure 3-S1A/Lane-control-GAPDH-3S1A.tiff]

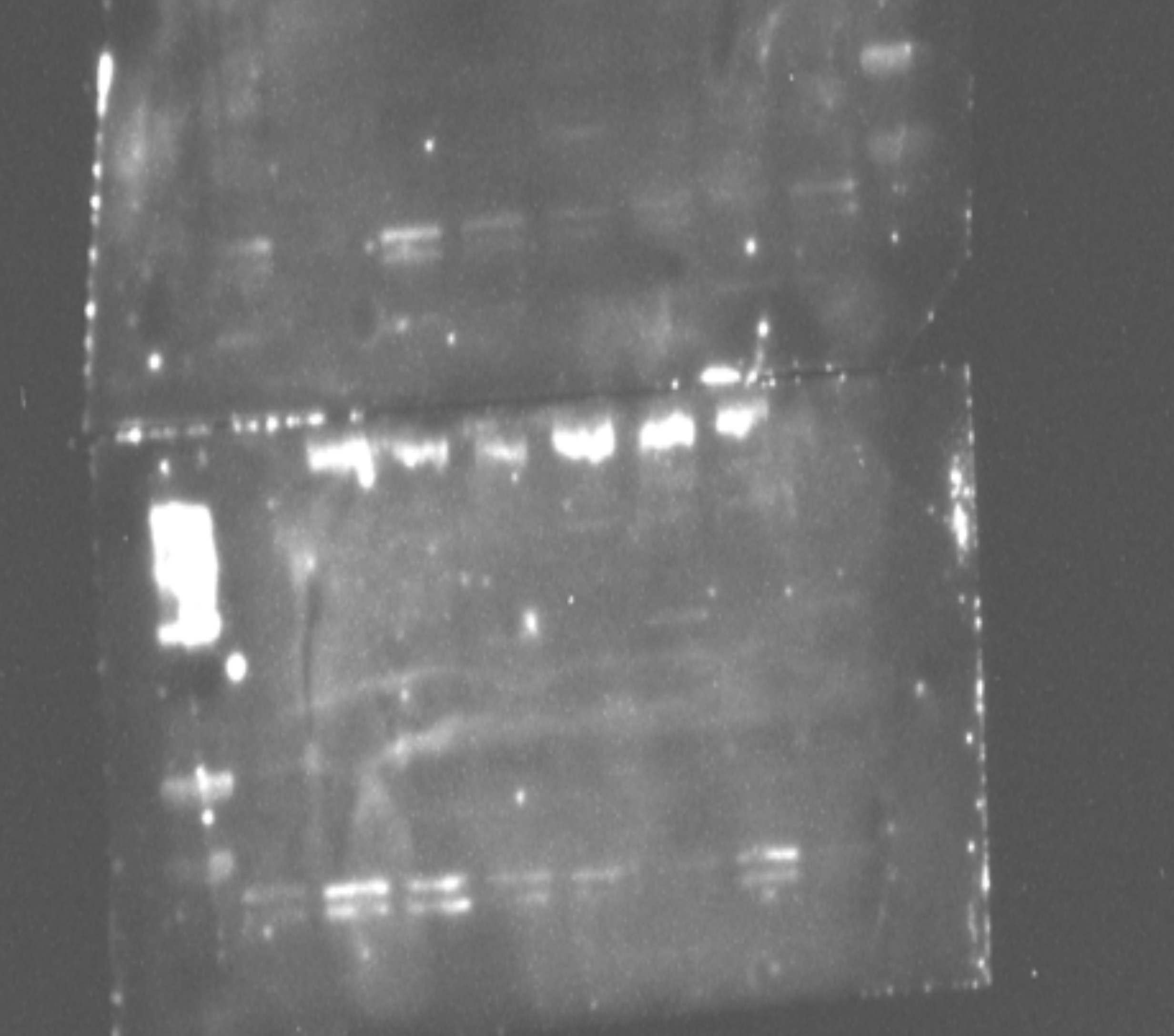

Supplement: Figure 3—figure supplement 1—source data 1. — Labelled (.pdf) and raw (folder) blot images showed in panels A,D, and E are also included. [file elife-79840-fig3-figsupp1-data1.zip › Figure 3-figure supplement 1 - source data/Blot Figure 3 - figure supplement 1/Figure 3-S1A/Lane-IPF-PRRX1-3S1A.tiff]

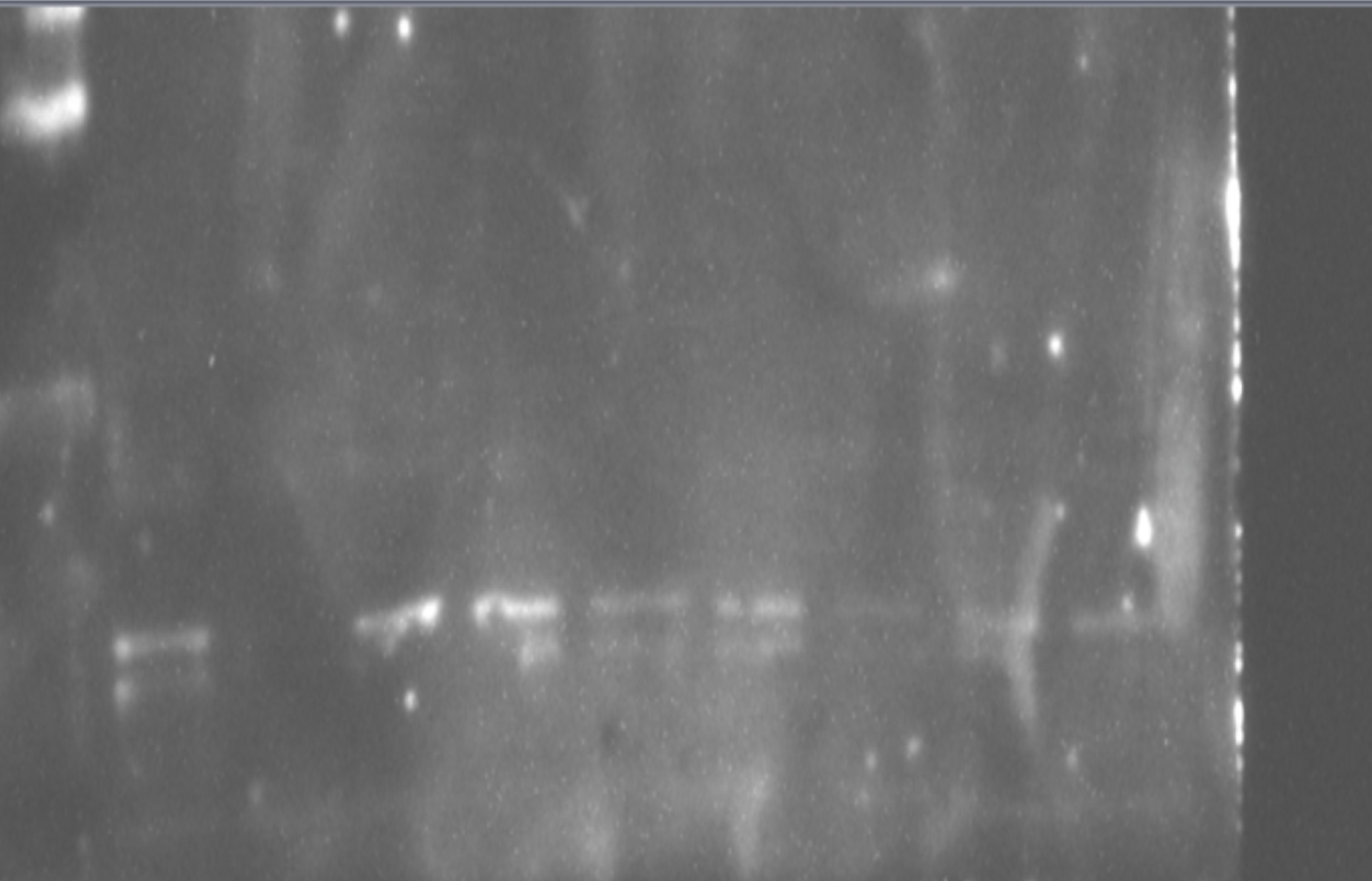

Supplement: Figure 3—figure supplement 1—source data 1. — Labelled (.pdf) and raw (folder) blot images showed in panels A,D, and E are also included. [file elife-79840-fig3-figsupp1-data1.zip › Figure 3-figure supplement 1 - source data/Blot Figure 3 - figure supplement 1/Figure 3-S1A/Lane-control-PRRX1-3S1A.tiff]

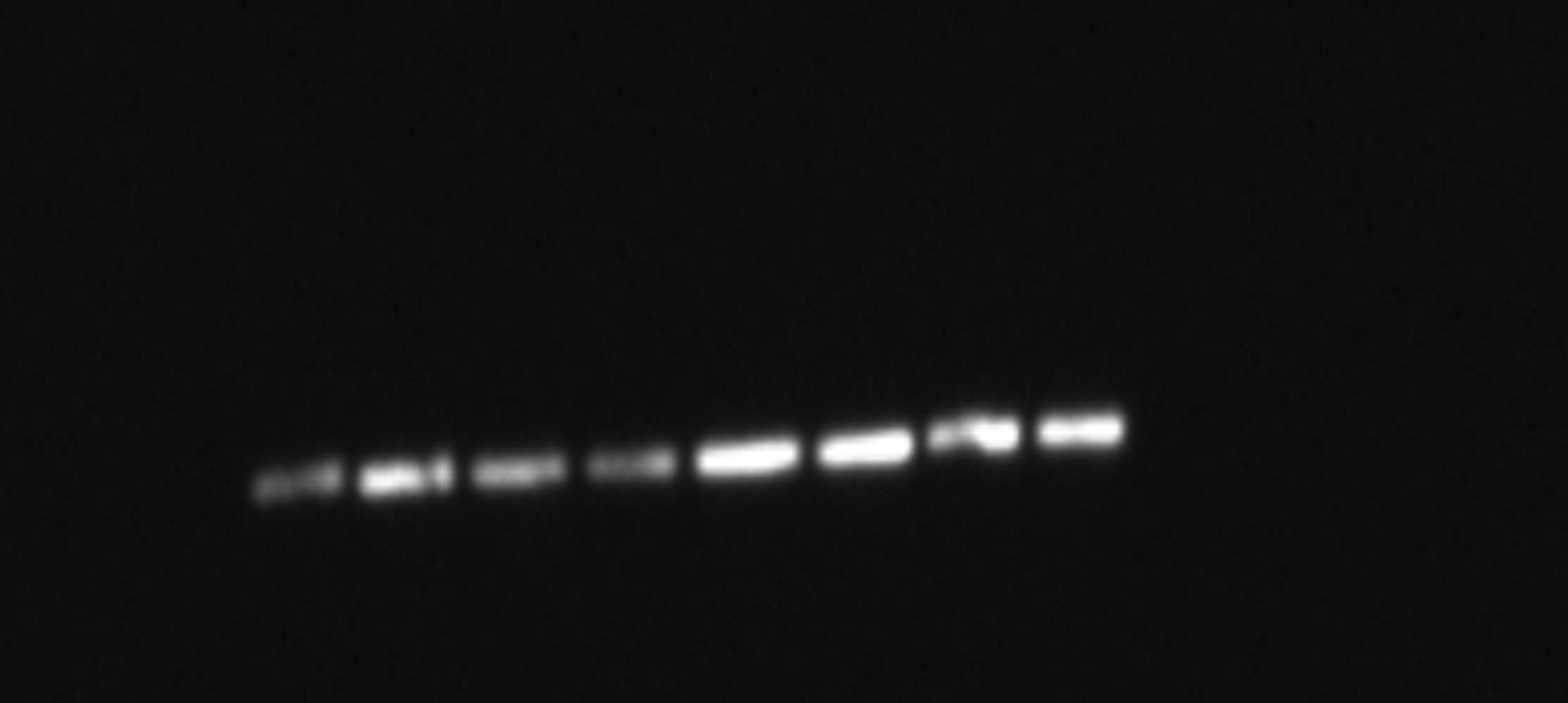

Supplement: Figure 3—figure supplement 1—source data 1. — Labelled (.pdf) and raw (folder) blot images showed in panels A,D, and E are also included. [file elife-79840-fig3-figsupp1-data1.zip › Figure 3-figure supplement 1 - source data/Blot Figure 3 - figure supplement 1/Figure 3-S1A/Lane-IPF-GAPDH-3S1A.tiff]

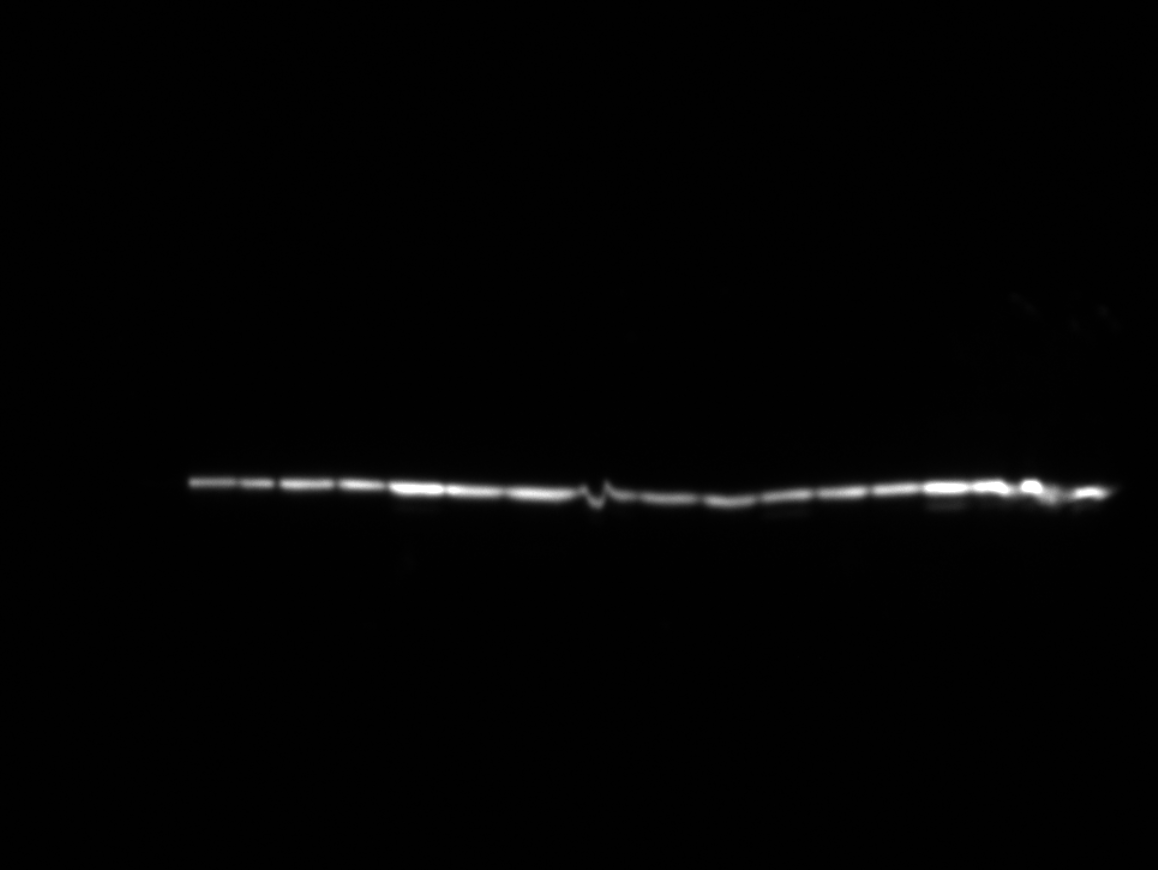

Supplement: Figure 4—source data 1. [file elife-79840-fig4-data1.zip › Figure 4 - source data/ blot Figure 4B/Lane-IPF-GAPDH-4B.tif]

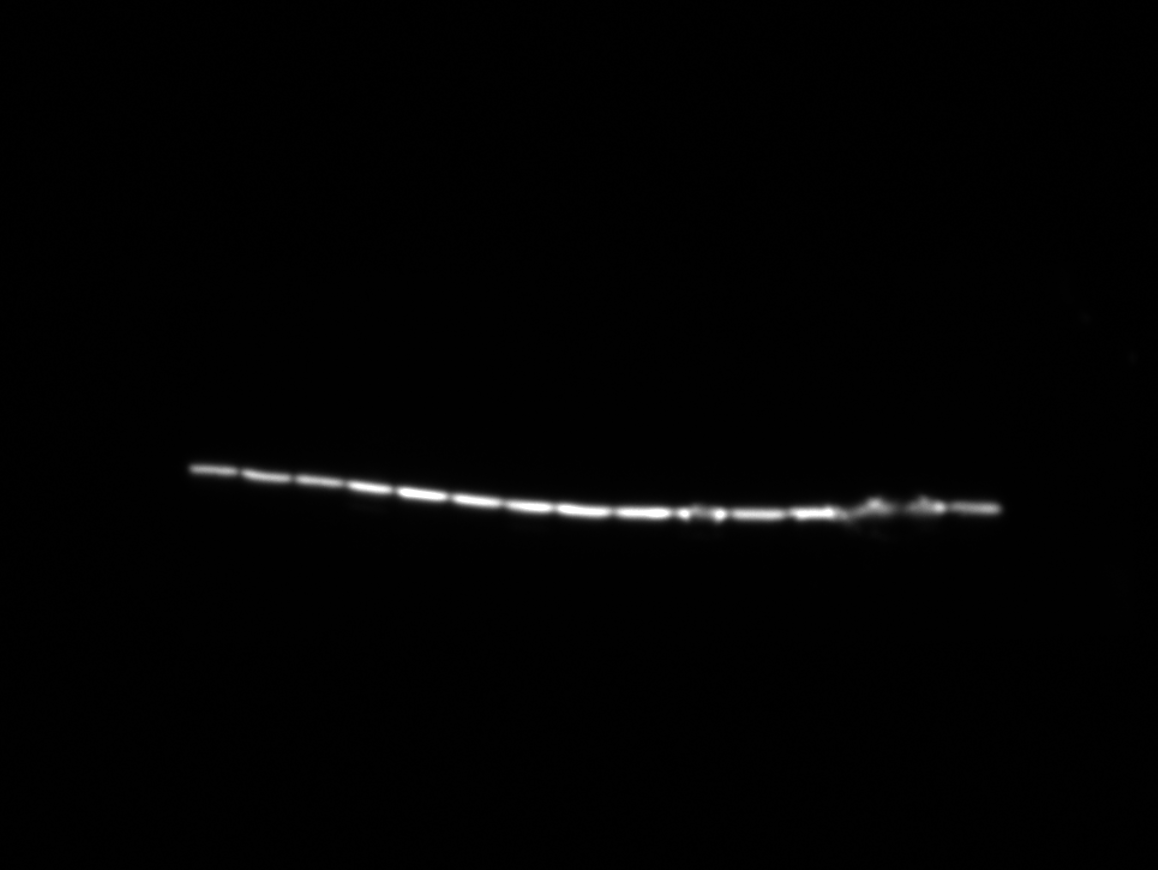

Supplement: Figure 4—source data 1. [file elife-79840-fig4-data1.zip › Figure 4 - source data/ blot Figure 4B/Lane-Control-GAPDH-4B.tif]

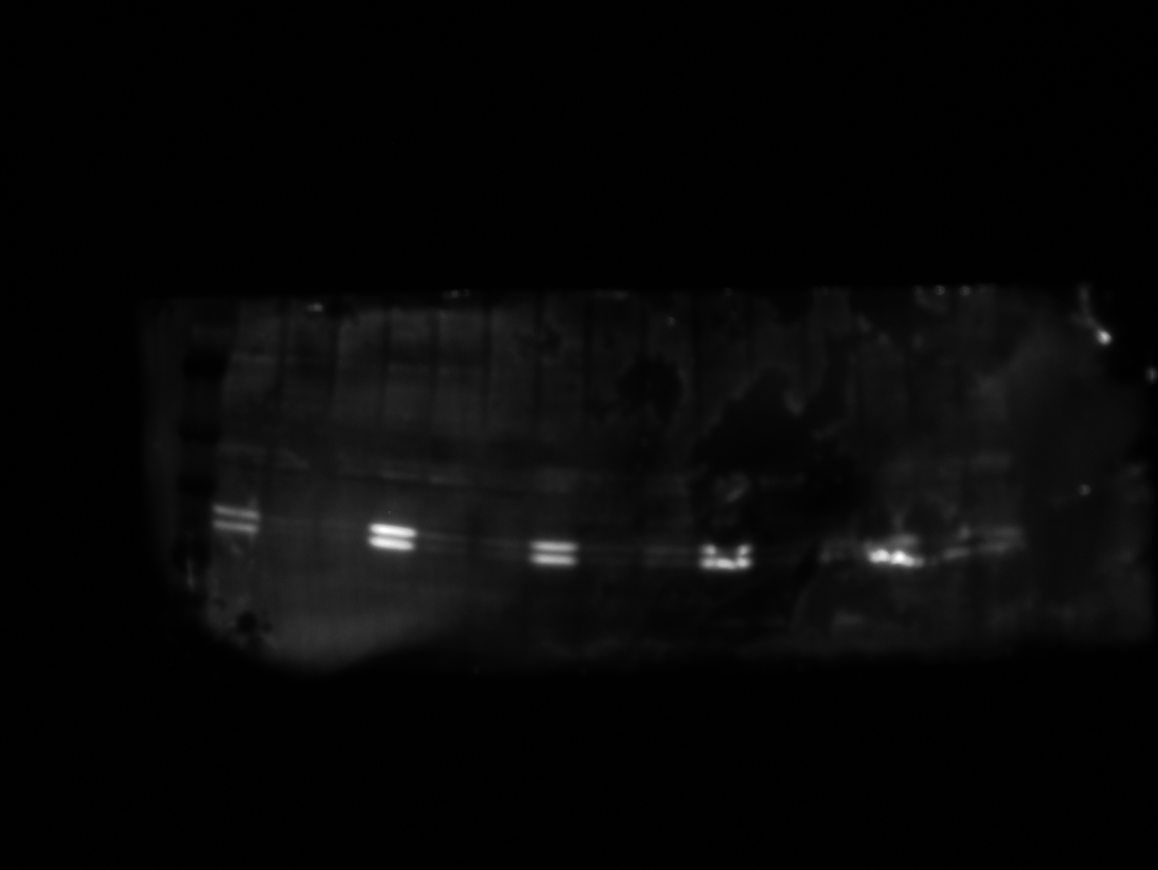

Supplement: Figure 4—source data 1. [file elife-79840-fig4-data1.zip › Figure 4 - source data/ blot Figure 4B/Lane-control-PRRX1-4B.tif]

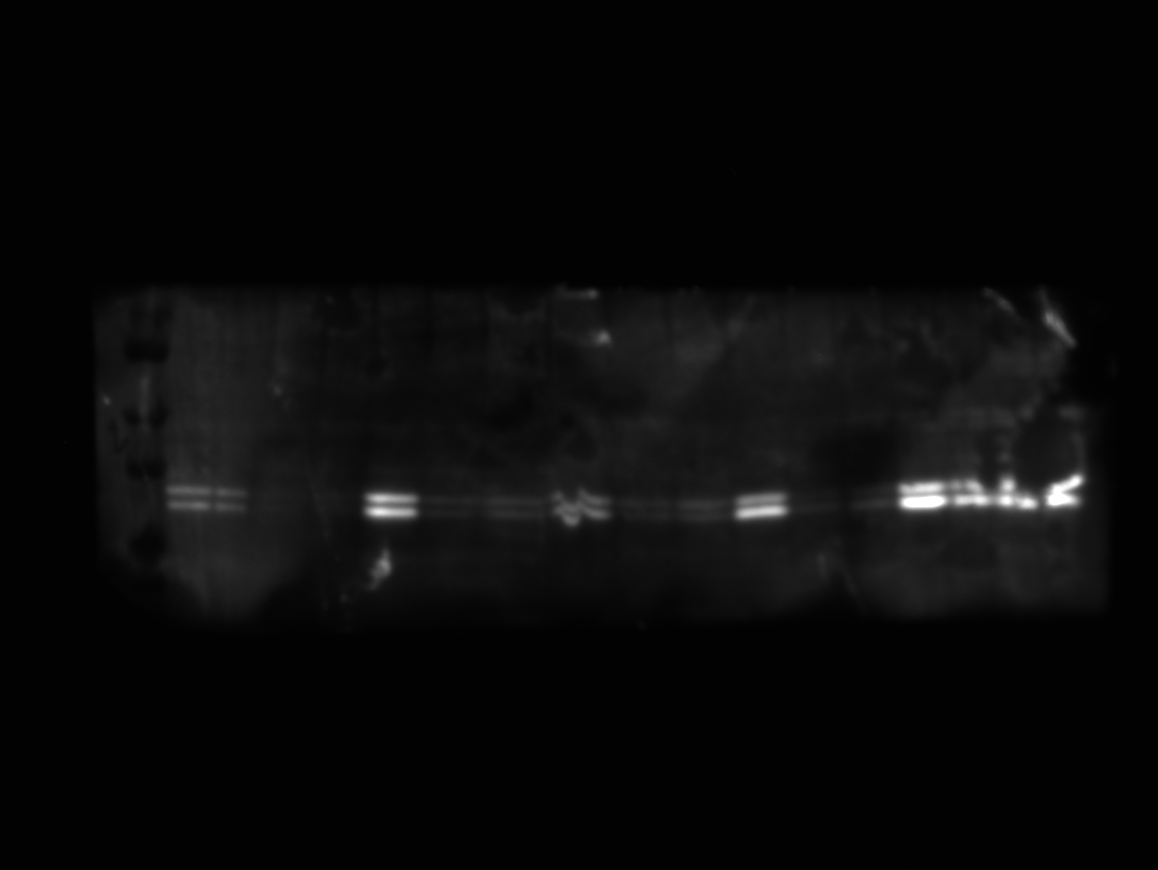

Supplement: Figure 4—source data 1. [file elife-79840-fig4-data1.zip › Figure 4 - source data/ blot Figure 4B/Lane-IPF-PRRX1-4B.tif]

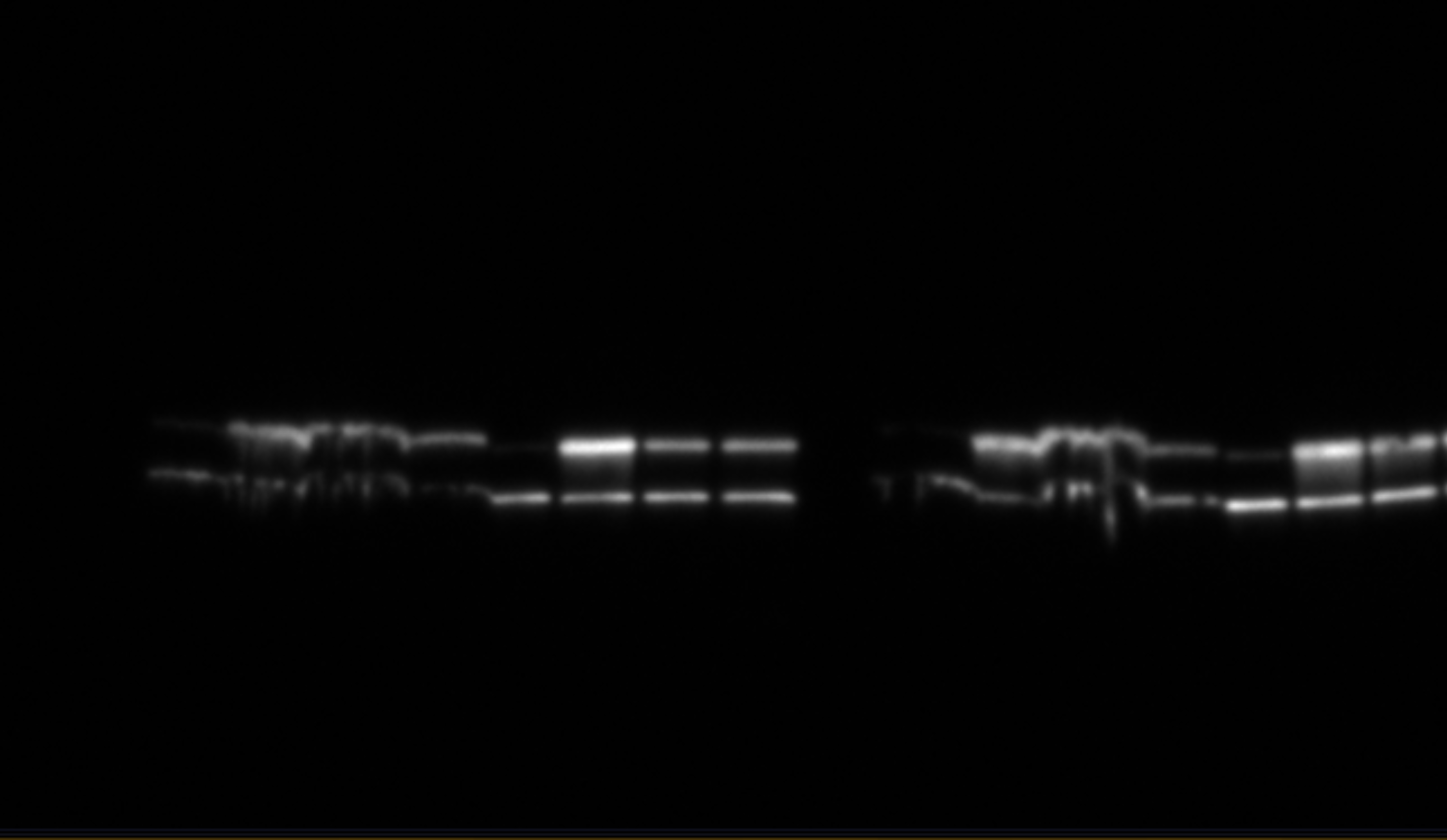

Supplement: Figure 5—source data 1. — Labelled (.pdf) and raw (folder) blot images showed in panels B and E are also included. [file elife-79840-fig5-data1.zip › Figure 5 - source data/ blot Figure 5B/Lane-ACTA2-GAPDH-control-5B.tiff]

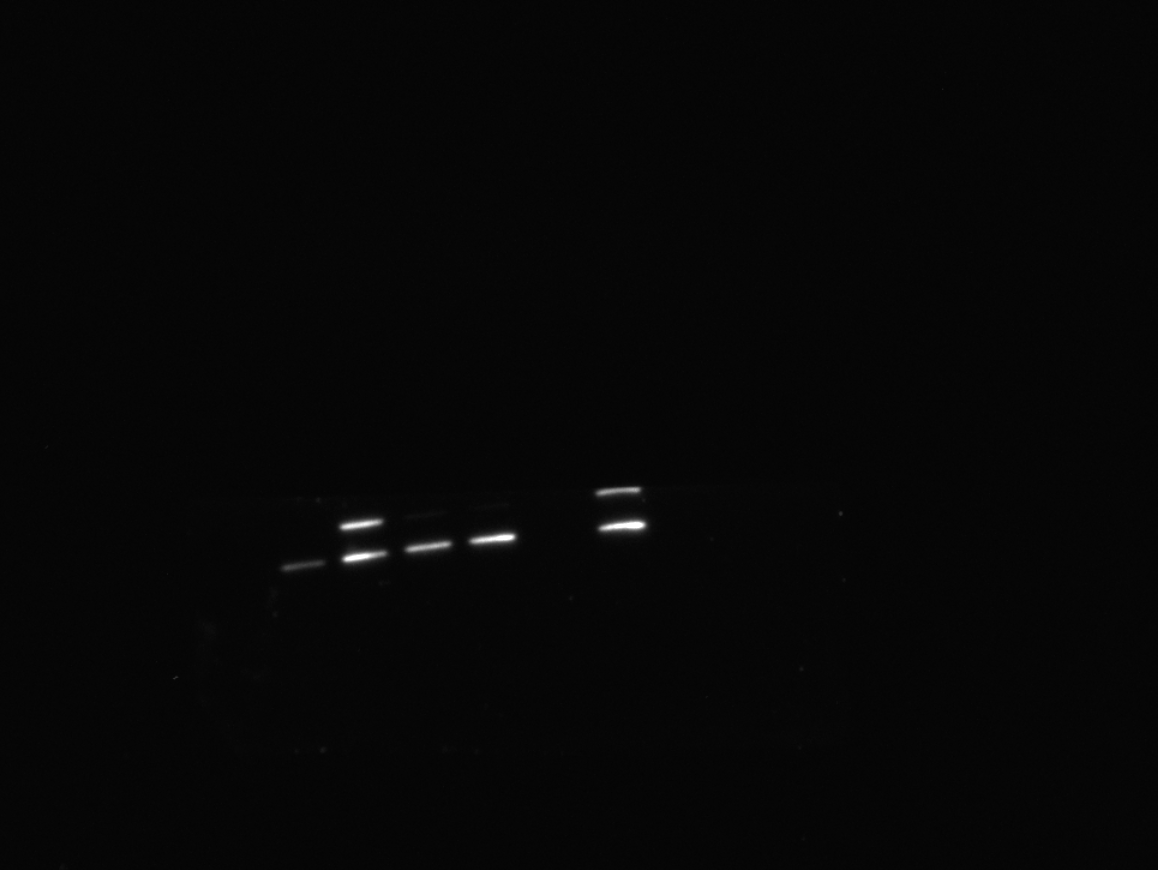

Supplement: Figure 5—source data 1. — Labelled (.pdf) and raw (folder) blot images showed in panels B and E are also included. [file elife-79840-fig5-data1.zip › Figure 5 - source data/ blot Figure 5B/Lane-ACTA2-GAPDH-IPF-5B.tiff]

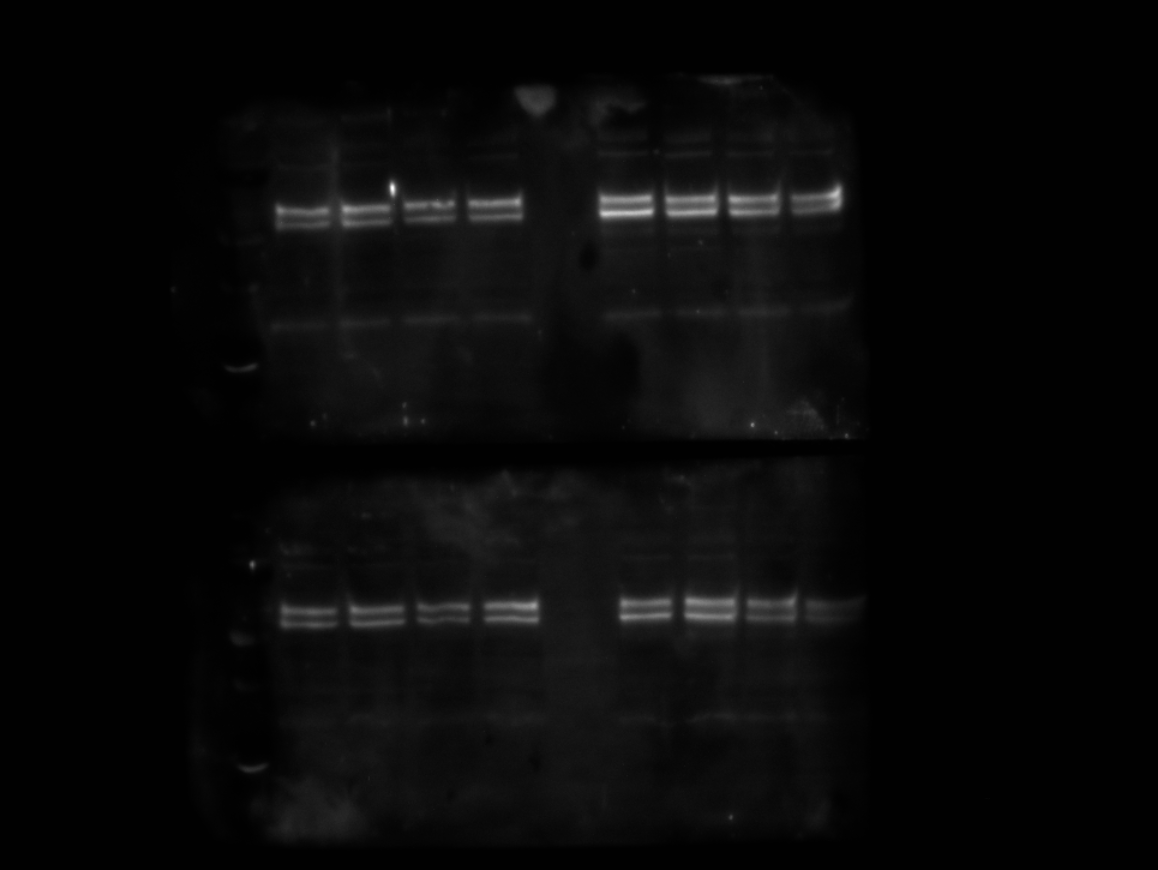

Supplement: Figure 5—source data 1. — Labelled (.pdf) and raw (folder) blot images showed in panels B and E are also included. [file elife-79840-fig5-data1.zip › Figure 5 - source data/ blot Figure 5E/Lane-totSMAD-5E.tiff]

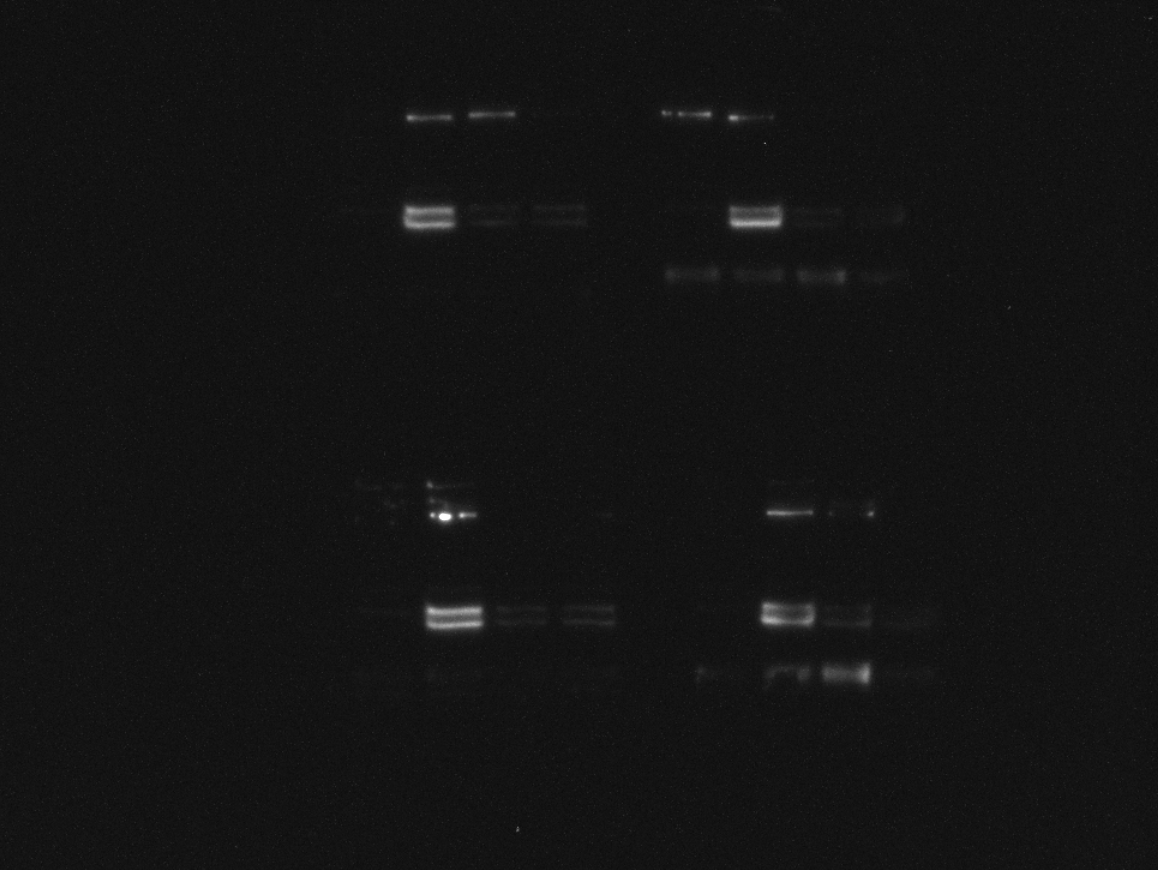

Supplement: Figure 5—source data 1. — Labelled (.pdf) and raw (folder) blot images showed in panels B and E are also included. [file elife-79840-fig5-data1.zip › Figure 5 - source data/ blot Figure 5E/Lane-pSMAD-5E.tiff]

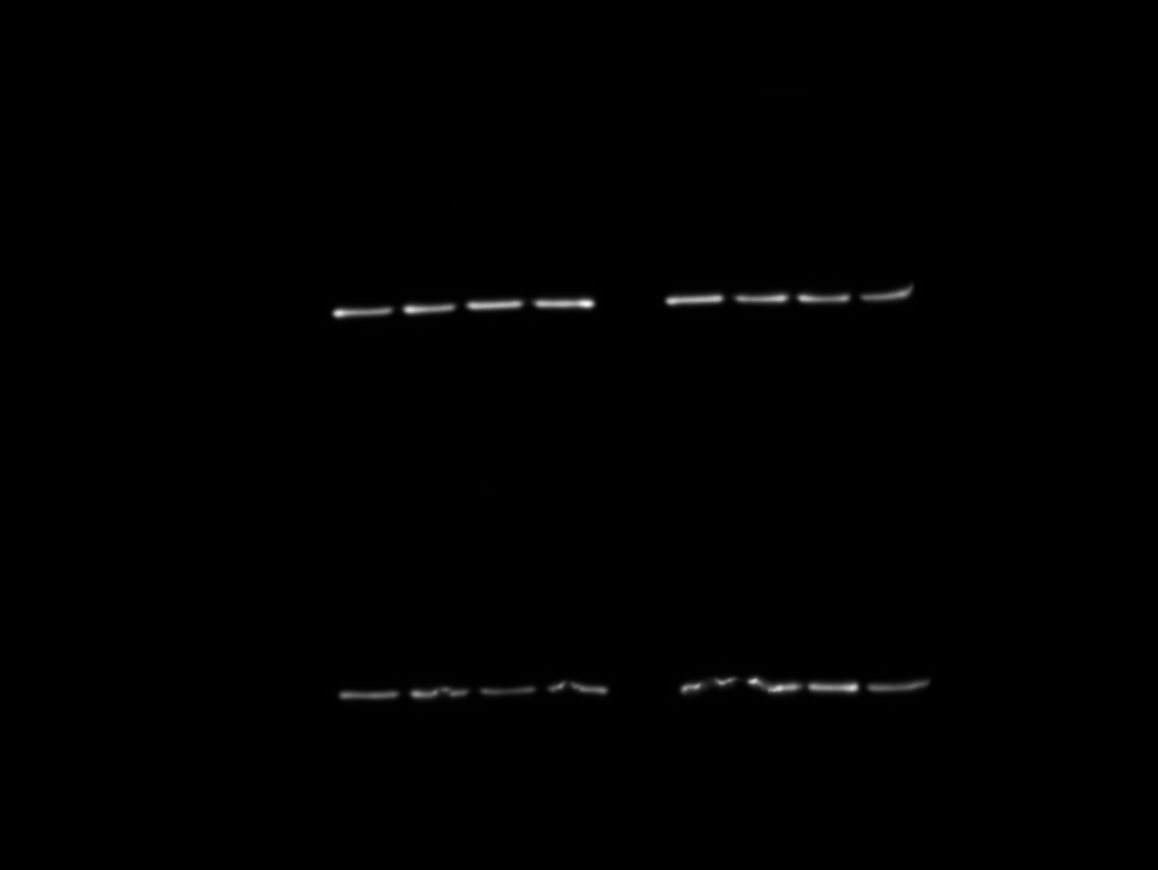

Supplement: Figure 5—source data 1. — Labelled (.pdf) and raw (folder) blot images showed in panels B and E are also included. [file elife-79840-fig5-data1.zip › Figure 5 - source data/ blot Figure 5E/Lane-GAPDH-5E.tiff]

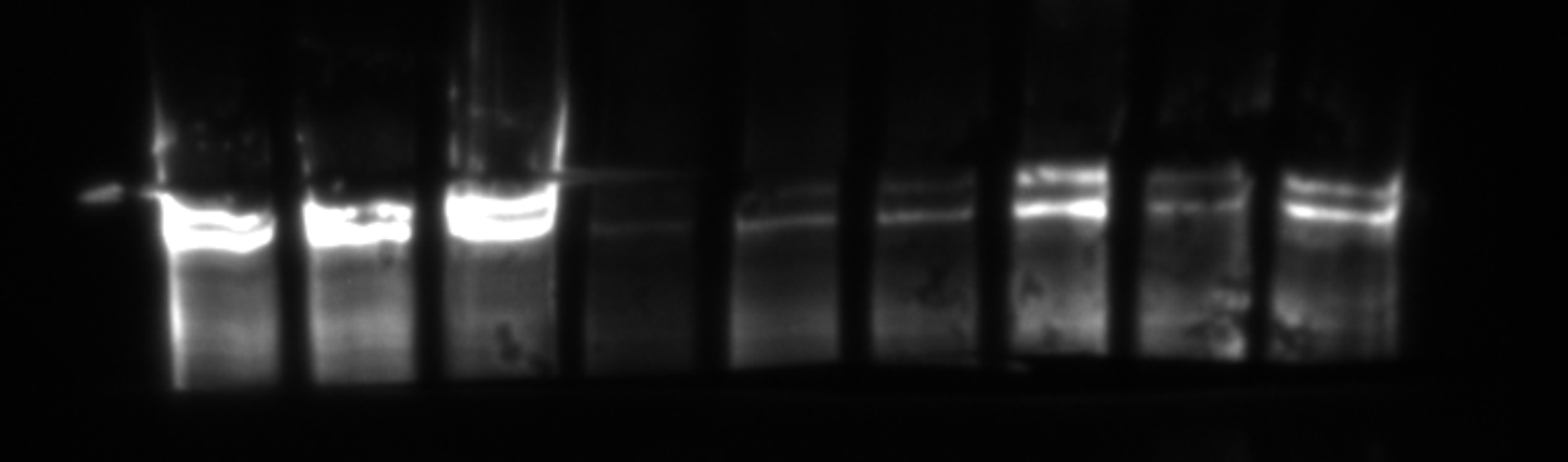

Supplement: Figure 5—figure supplement 1—source data 1. — Labelled (.pdf) and raw (folder) blot images showed in panel B are also included. [file elife-79840-fig5-figsupp1-data1.zip › Figure 5 - supplement Figure 1 - source data/blot Figure 5 - figure supplement S1/Figure 5-S1B/Lane-COL1-Control-5S1B.tiff]

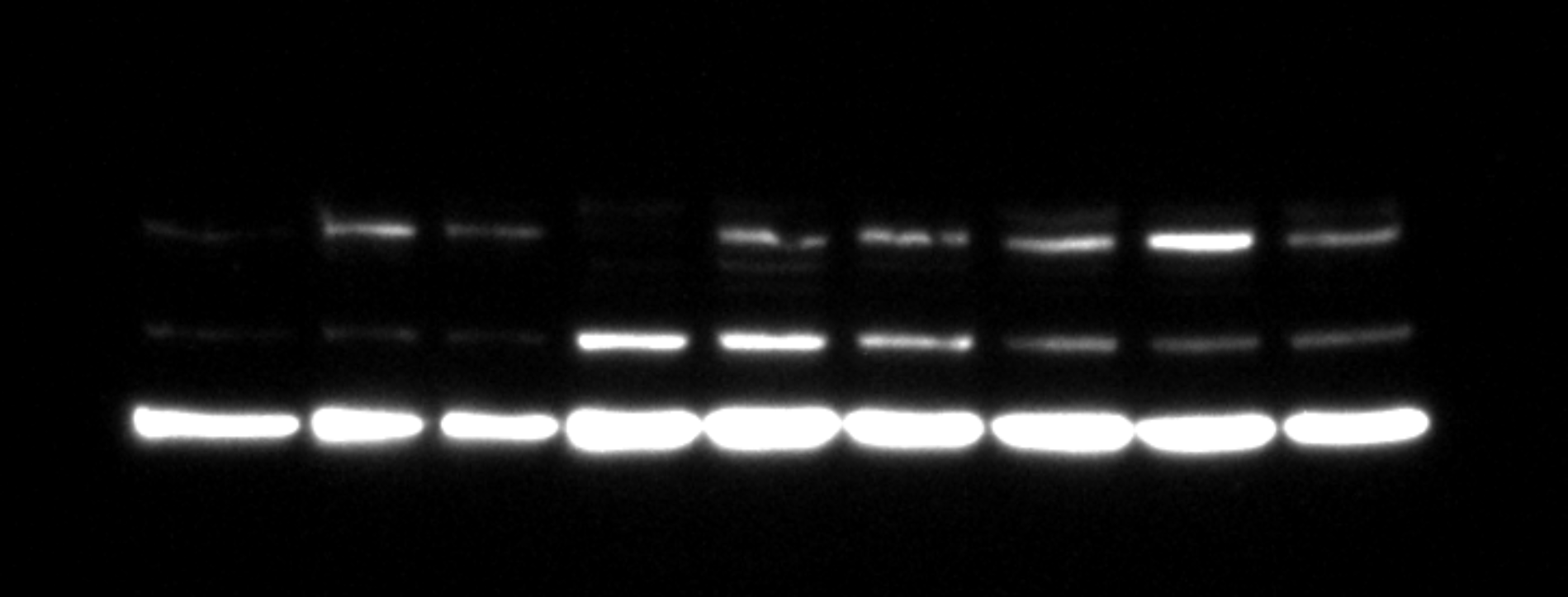

Supplement: Figure 5—figure supplement 1—source data 1. — Labelled (.pdf) and raw (folder) blot images showed in panel B are also included. [file elife-79840-fig5-figsupp1-data1.zip › Figure 5 - supplement Figure 1 - source data/blot Figure 5 - figure supplement S1/Figure 5-S1B/Lane-ACTA2-GAPDH-Control-5S1B.tiff]

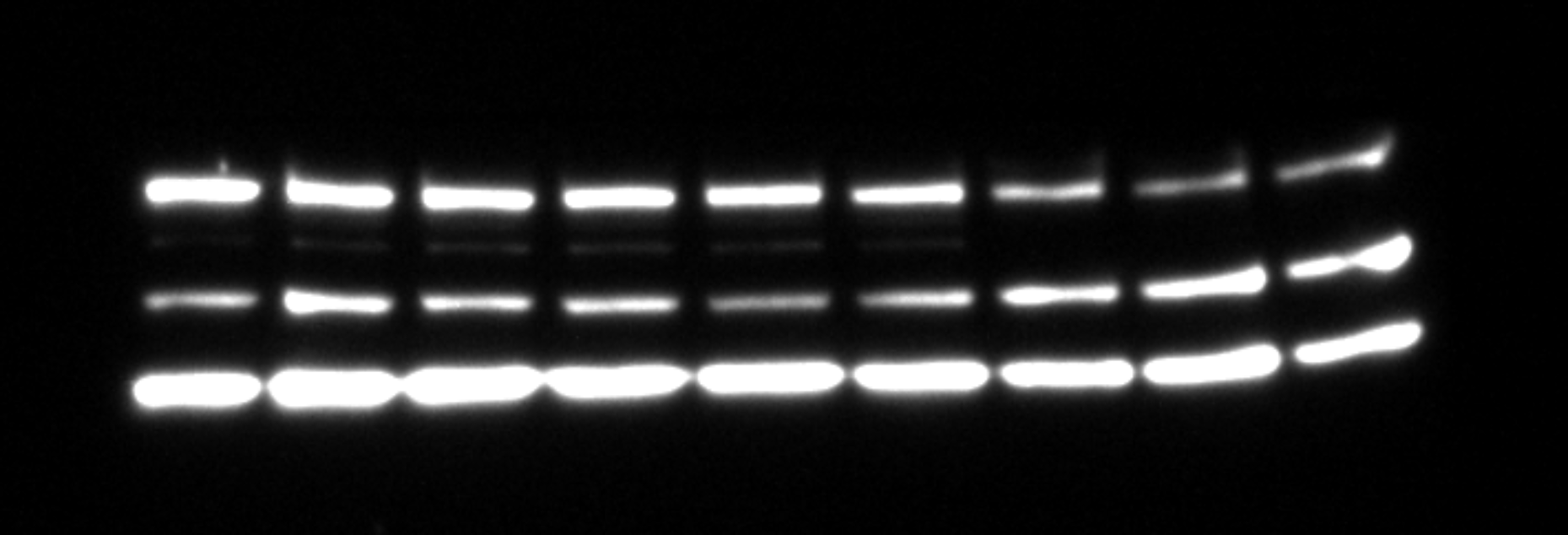

Supplement: Figure 5—figure supplement 1—source data 1. — Labelled (.pdf) and raw (folder) blot images showed in panel B are also included. [file elife-79840-fig5-figsupp1-data1.zip › Figure 5 - supplement Figure 1 - source data/blot Figure 5 - figure supplement S1/Figure 5-S1B/Lane-ACTA2-GAPDH-IPF-5S1B.tiff]

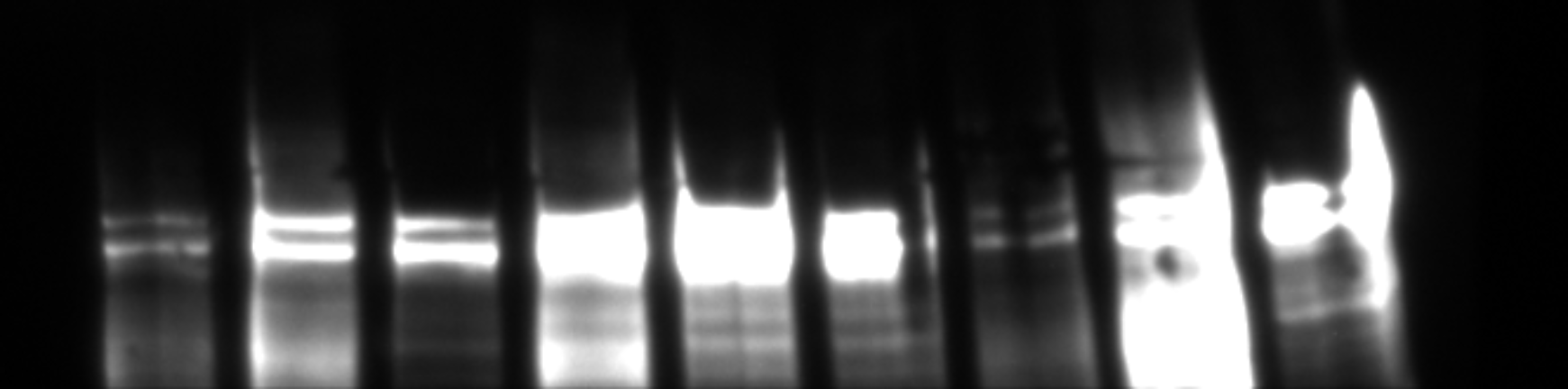

Supplement: Figure 5—figure supplement 1—source data 1. — Labelled (.pdf) and raw (folder) blot images showed in panel B are also included. [file elife-79840-fig5-figsupp1-data1.zip › Figure 5 - supplement Figure 1 - source data/blot Figure 5 - figure supplement S1/Figure 5-S1B/Lane-COL1-IPF-5S1B.tiff]

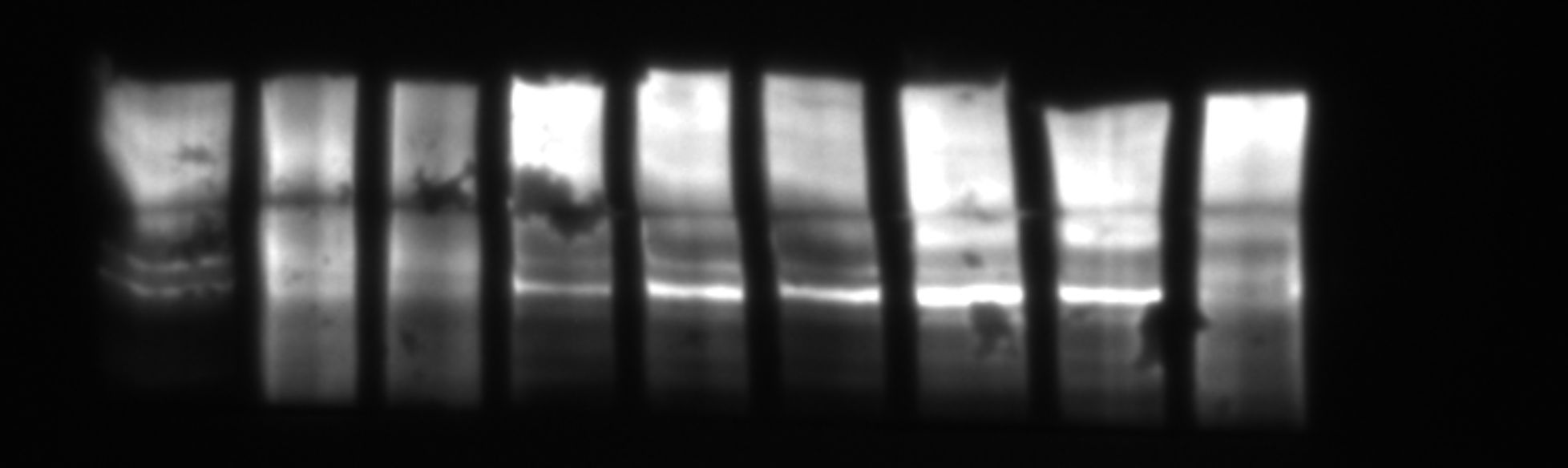

Supplement: Figure 5—figure supplement 1—source data 1. — Labelled (.pdf) and raw (folder) blot images showed in panel B are also included. [file elife-79840-fig5-figsupp1-data1.zip › Figure 5 - supplement Figure 1 - source data/blot Figure 5 - figure supplement S1/Figure 5-S1B/Lane-FN1-Control-5S1B.tiff]

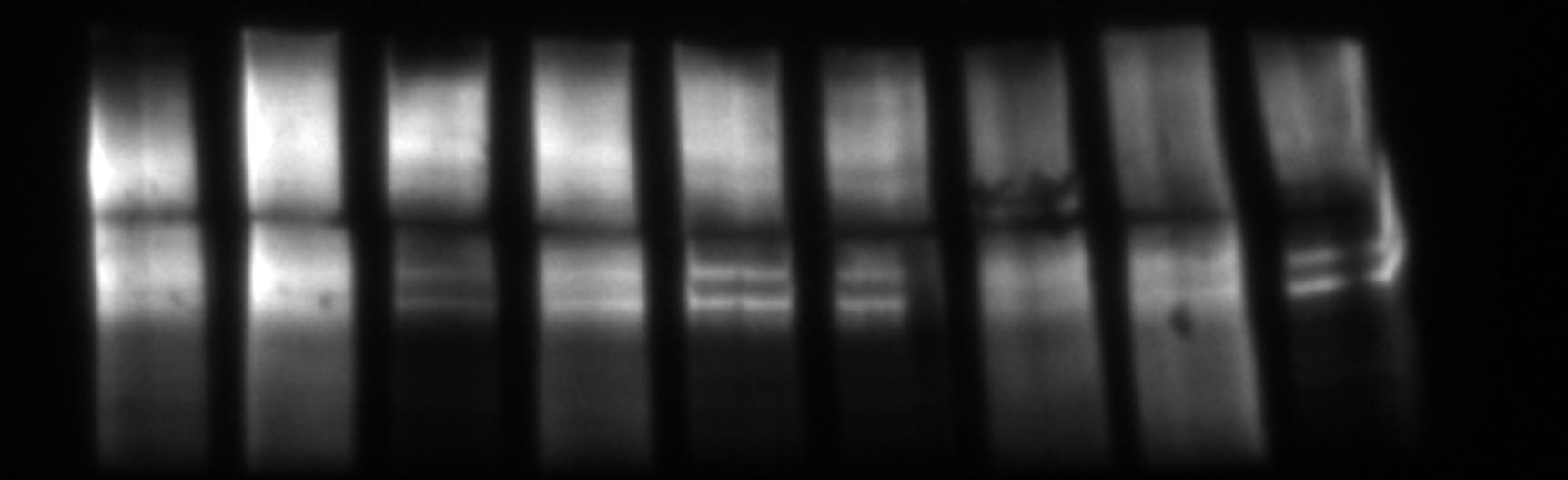

Supplement: Figure 5—figure supplement 1—source data 1. — Labelled (.pdf) and raw (folder) blot images showed in panel B are also included. [file elife-79840-fig5-figsupp1-data1.zip › Figure 5 - supplement Figure 1 - source data/blot Figure 5 - figure supplement S1/Figure 5-S1B/Lane-FN1-IPF-5S1B.tiff]

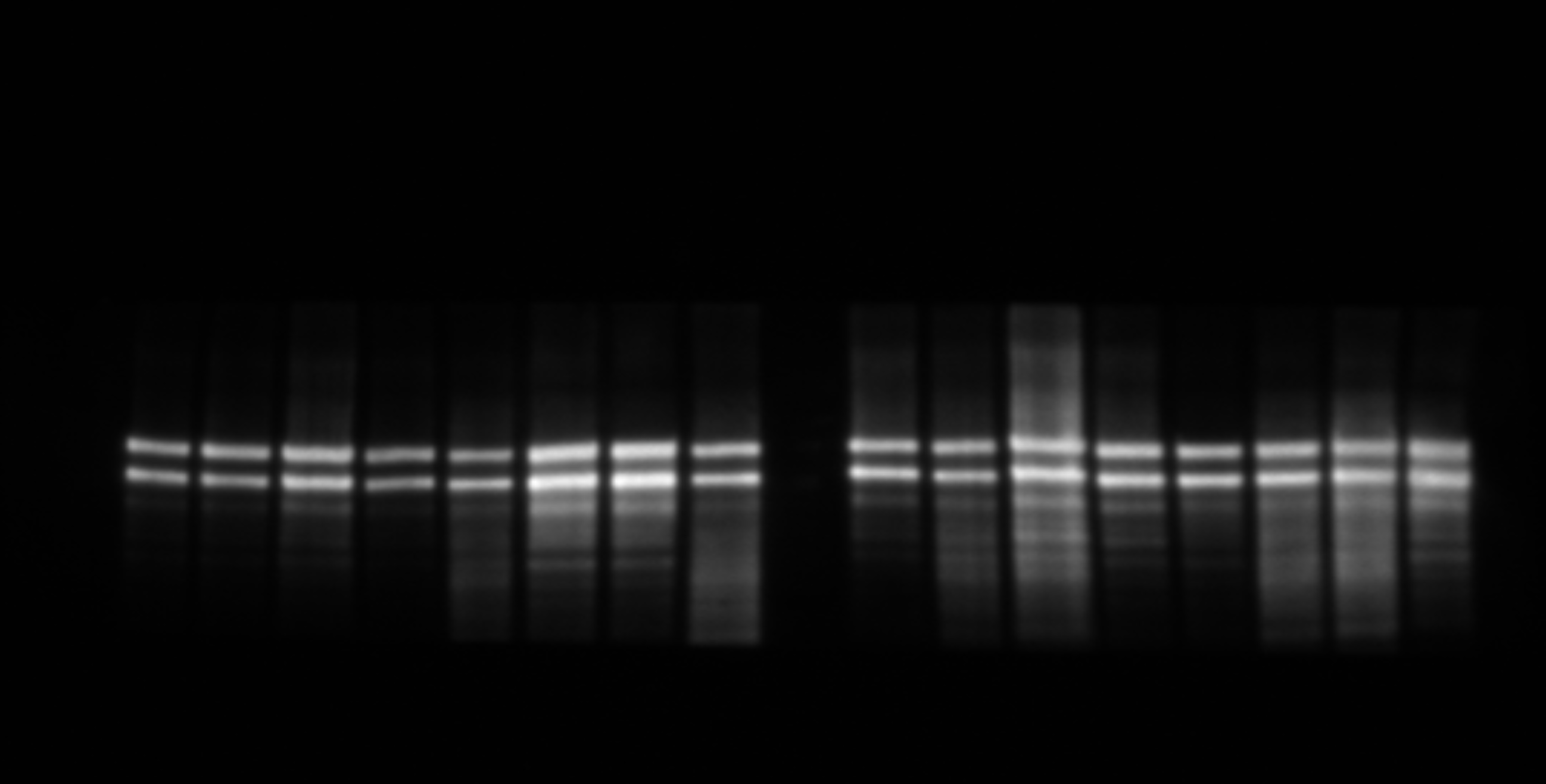

Supplement: Figure 5—figure supplement 2—source data 1. — Labelled (.pdf) and raw (folder) blot images showed in panel C are also included. [file elife-79840-fig5-figsupp2-data1.zip › Figure 5 - supplement Figure 2 - source data/blot Figure 5 - figure supplement S2/Figure 5-S2C/Lane-COL1-IPF-5S2C.tiff]

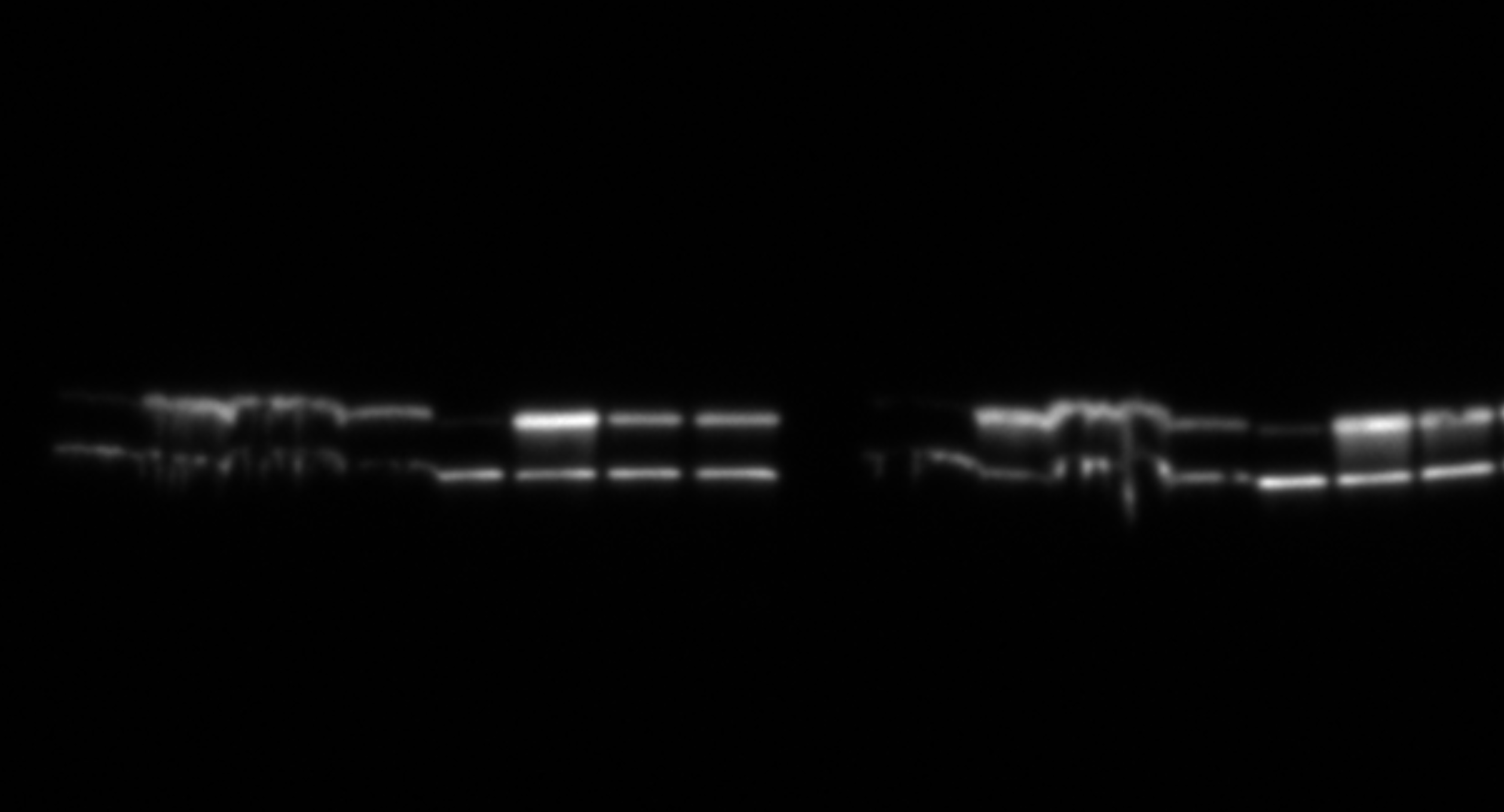

Supplement: Figure 5—figure supplement 2—source data 1. — Labelled (.pdf) and raw (folder) blot images showed in panel C are also included. [file elife-79840-fig5-figsupp2-data1.zip › Figure 5 - supplement Figure 2 - source data/blot Figure 5 - figure supplement S2/Figure 5-S2C/Lane-GAPDH-Control-5S2C.tiff]

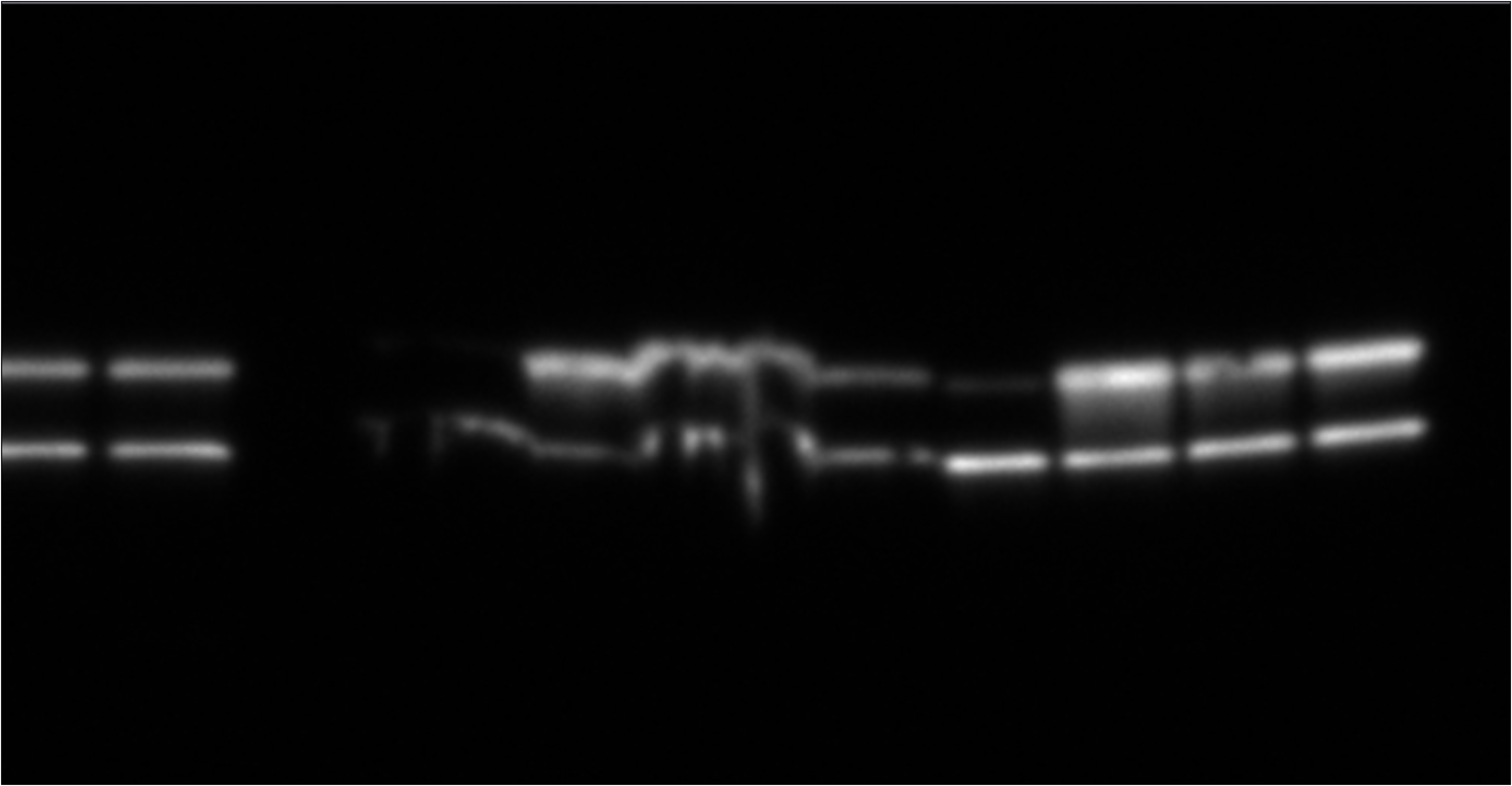

Supplement: Figure 5—figure supplement 2—source data 1. — Labelled (.pdf) and raw (folder) blot images showed in panel C are also included. [file elife-79840-fig5-figsupp2-data1.zip › Figure 5 - supplement Figure 2 - source data/blot Figure 5 - figure supplement S2/Figure 5-S2C/Lane-GAPDH-IPF-5S2C.tiff]

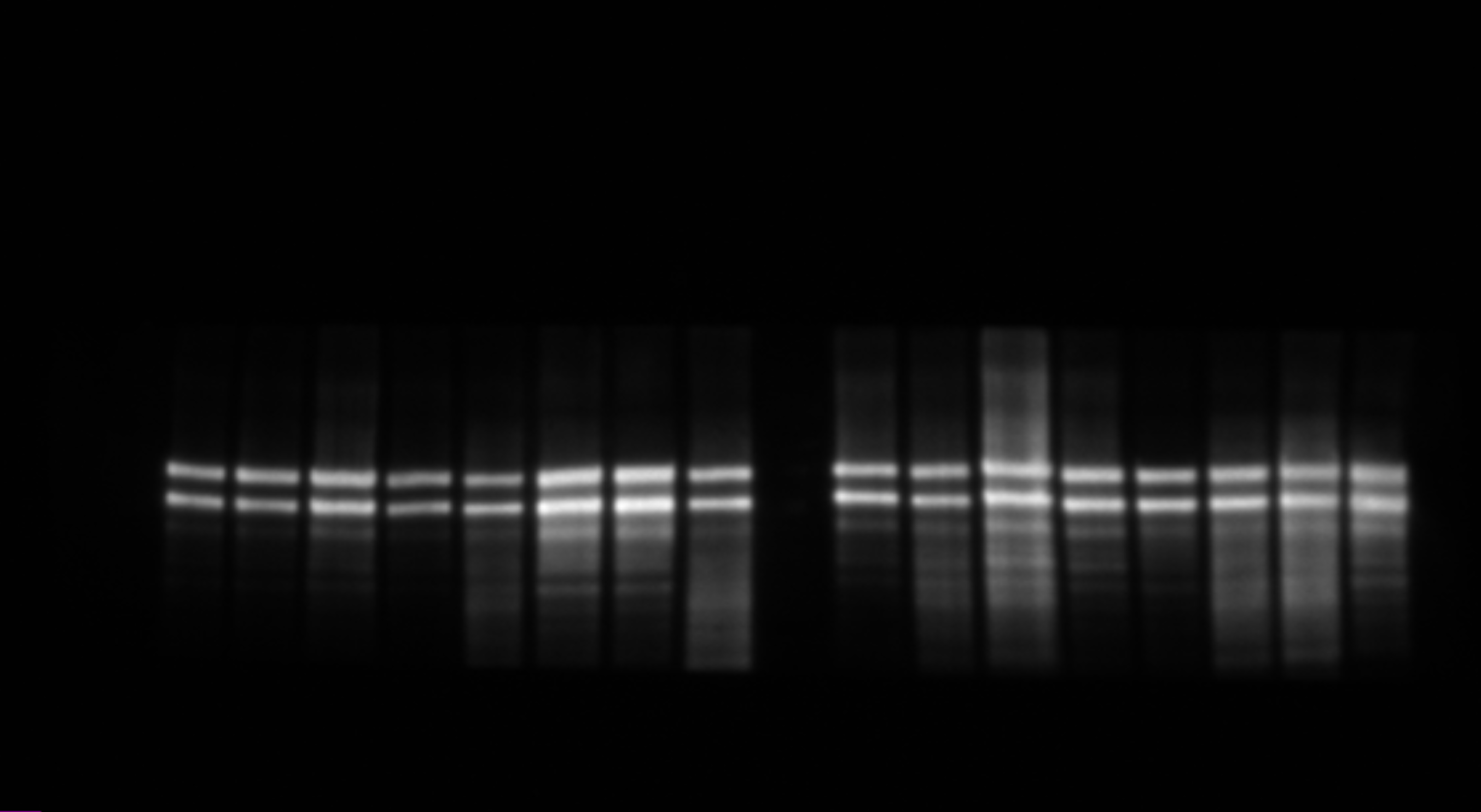

Supplement: Figure 5—figure supplement 2—source data 1. — Labelled (.pdf) and raw (folder) blot images showed in panel C are also included. [file elife-79840-fig5-figsupp2-data1.zip › Figure 5 - supplement Figure 2 - source data/blot Figure 5 - figure supplement S2/Figure 5-S2C/Lane-COL1-Control-5S2C.tiff]

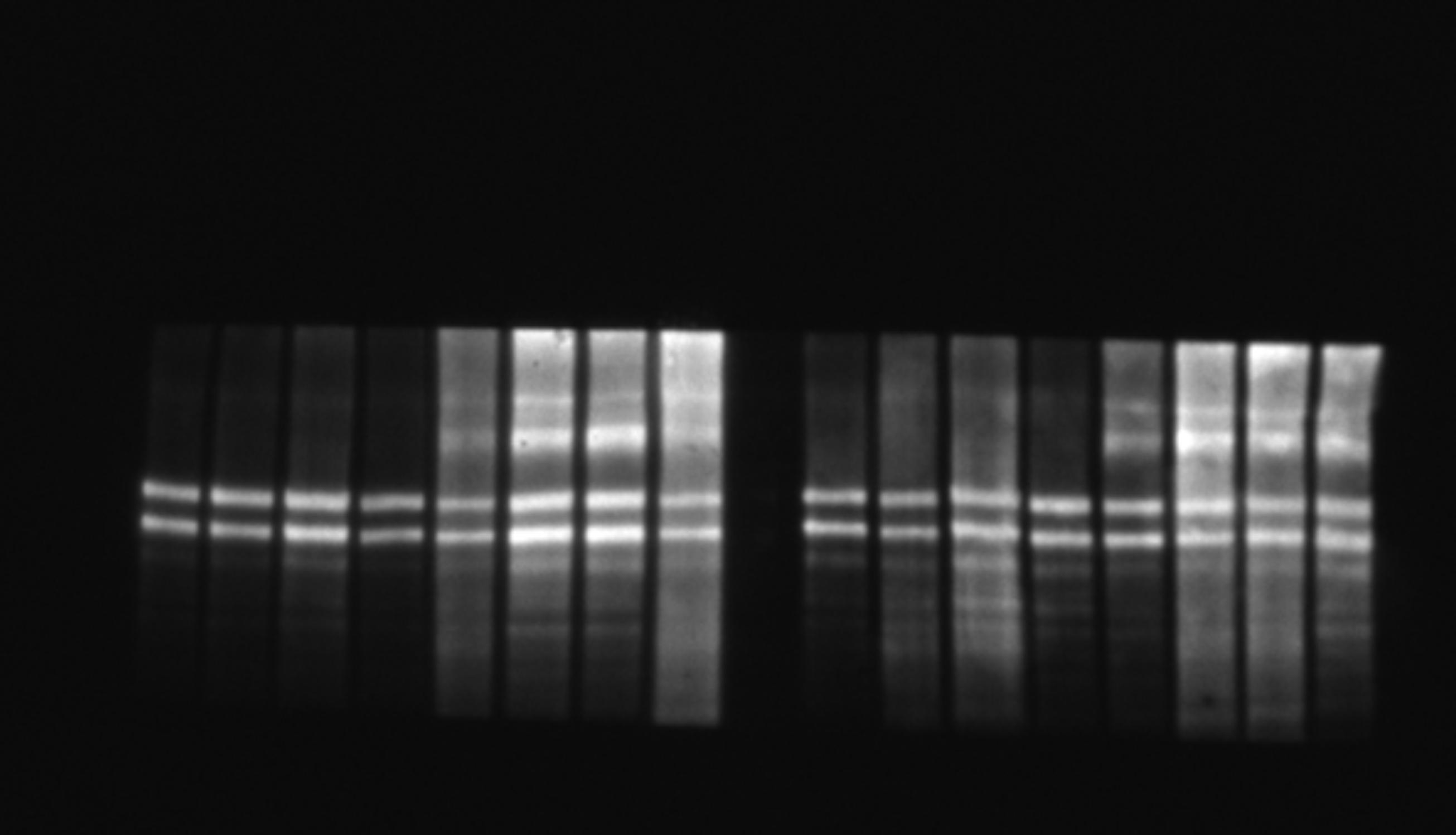

Supplement: Figure 5—figure supplement 2—source data 1. — Labelled (.pdf) and raw (folder) blot images showed in panel C are also included. [file elife-79840-fig5-figsupp2-data1.zip › Figure 5 - supplement Figure 2 - source data/blot Figure 5 - figure supplement S2/Figure 5-S2C/Lane-FN1-IPF-5S2C.tiff]

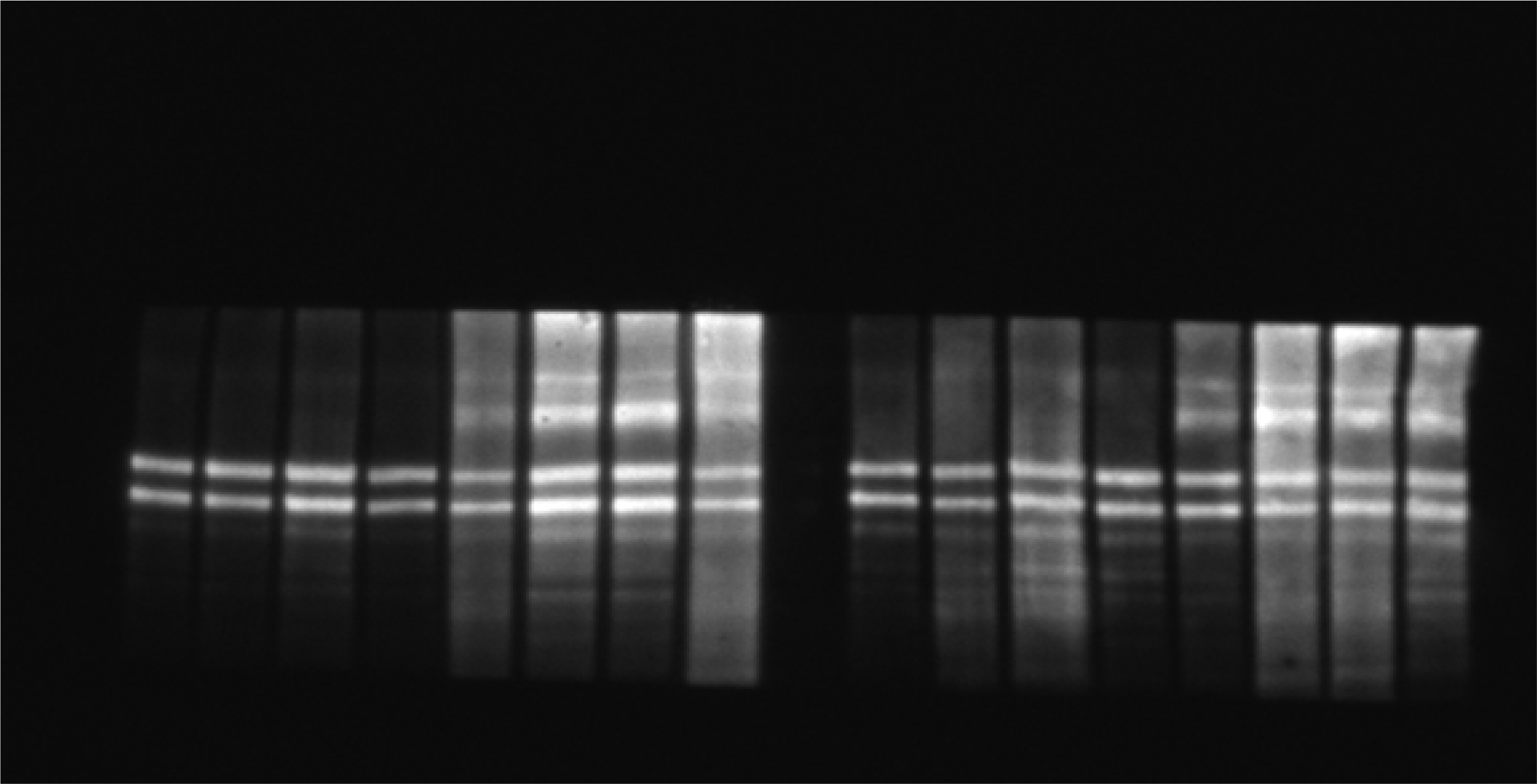

Supplement: Figure 5—figure supplement 2—source data 1. — Labelled (.pdf) and raw (folder) blot images showed in panel C are also included. [file elife-79840-fig5-figsupp2-data1.zip › Figure 5 - supplement Figure 2 - source data/blot Figure 5 - figure supplement S2/Figure 5-S2C/Lane-FN1-Control-5S2C.tiff]

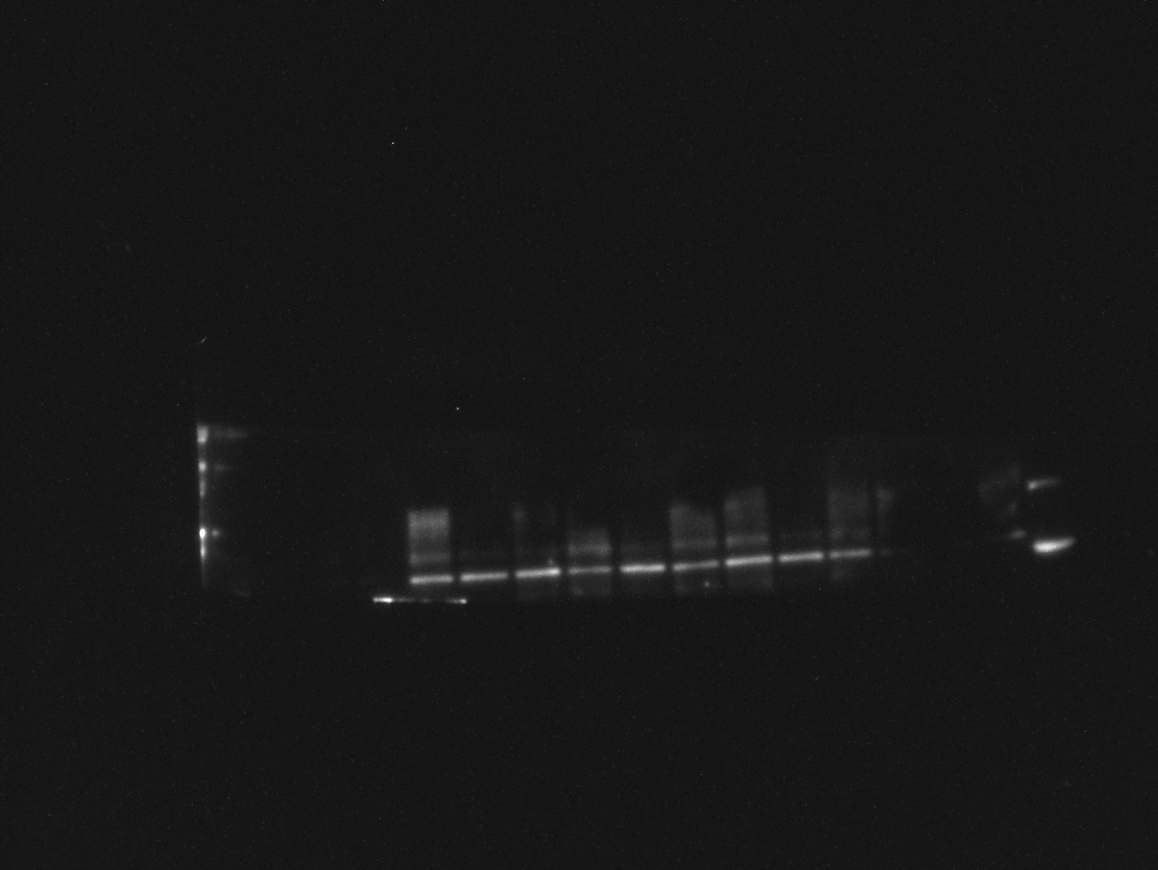

Supplement: Figure 5—figure supplement 4—source data 1. — Labelled (.pdf) and raw (folder) blot images showed in panels B and D are also included. [file elife-79840-fig5-figsupp4-data1.zip › Figure 5 - supplement Figure 4 - source data/blot Figure 5 - figure supplement S4/Figure 5-S4A/Lane-Control-TGFBR2-5S4A.tif]

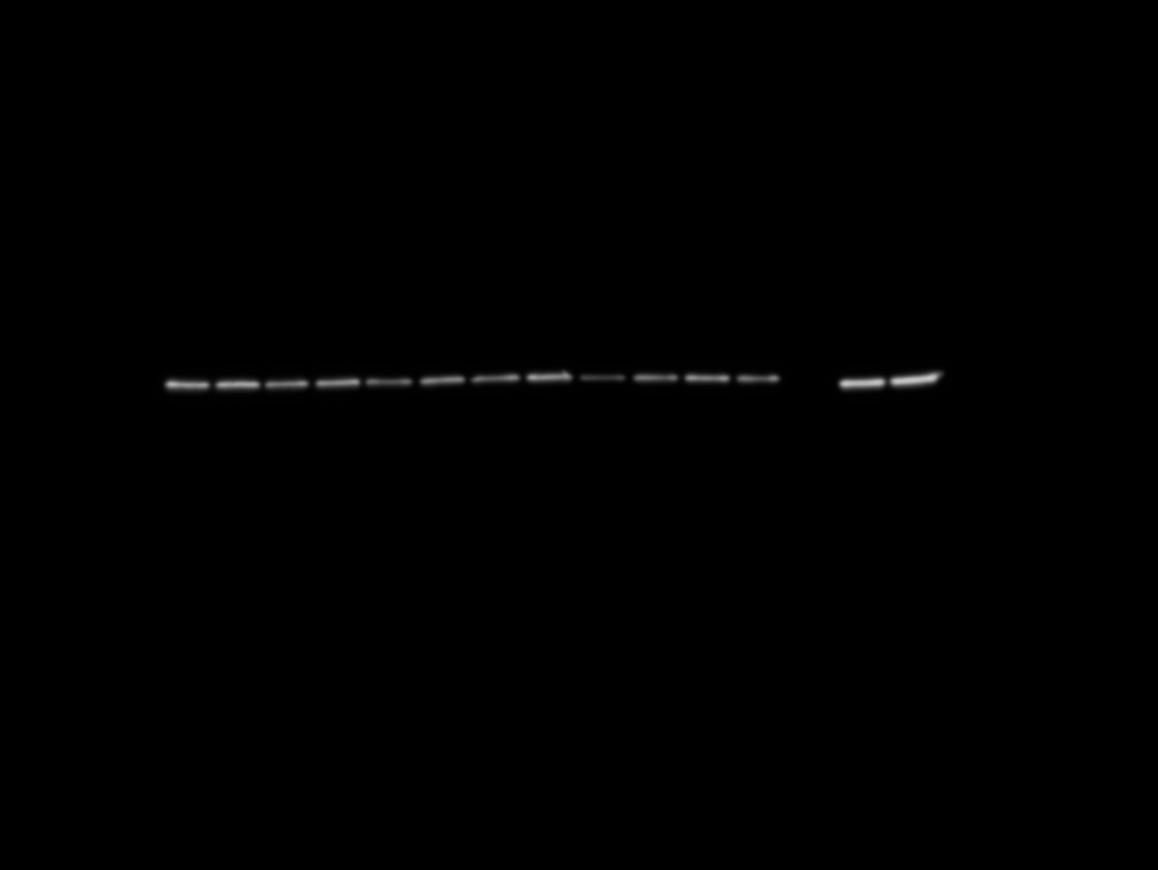

Supplement: Figure 5—figure supplement 4—source data 1. — Labelled (.pdf) and raw (folder) blot images showed in panels B and D are also included. [file elife-79840-fig5-figsupp4-data1.zip › Figure 5 - supplement Figure 4 - source data/blot Figure 5 - figure supplement S4/Figure 5-S4A/Lane_GAPDH_IPF-5S4A.tiff]

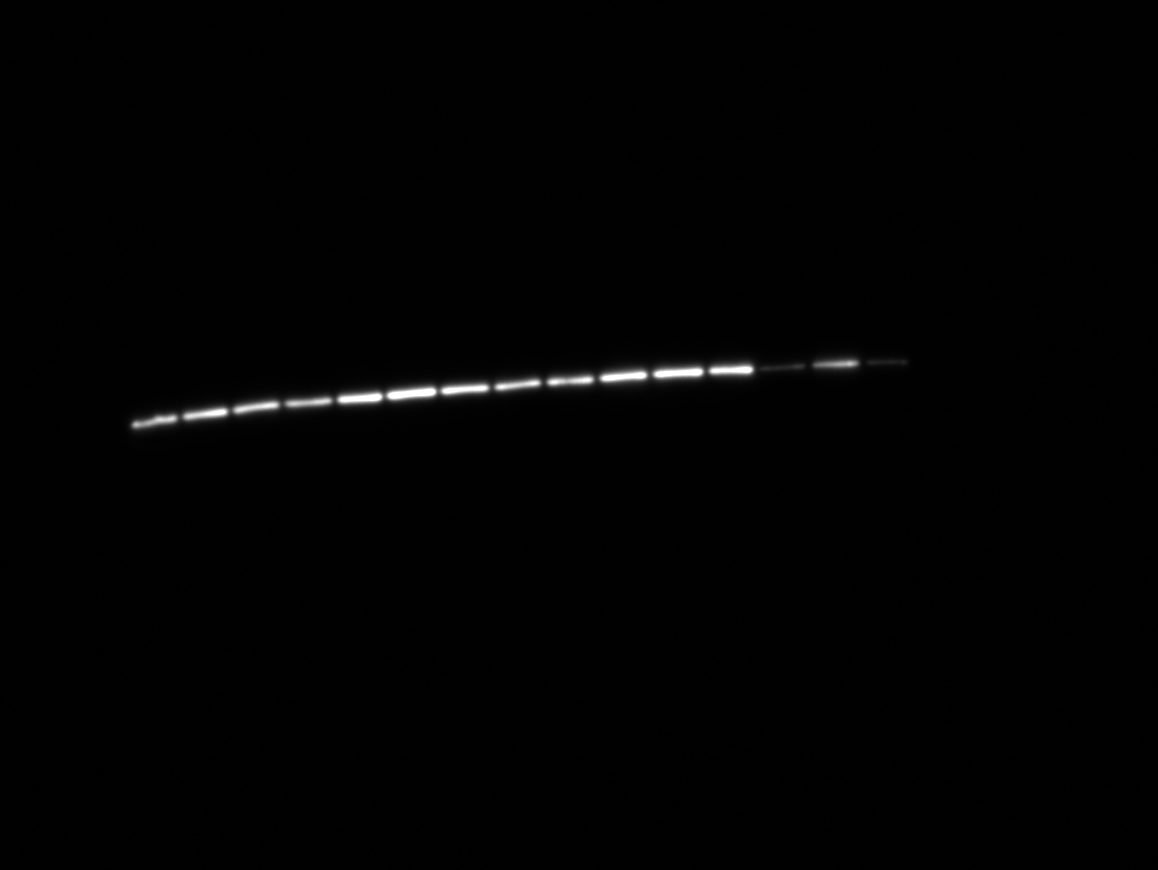

Supplement: Figure 5—figure supplement 4—source data 1. — Labelled (.pdf) and raw (folder) blot images showed in panels B and D are also included. [file elife-79840-fig5-figsupp4-data1.zip › Figure 5 - supplement Figure 4 - source data/blot Figure 5 - figure supplement S4/Figure 5-S4A/Lane-Control-GAPDH-5S4A.tif]

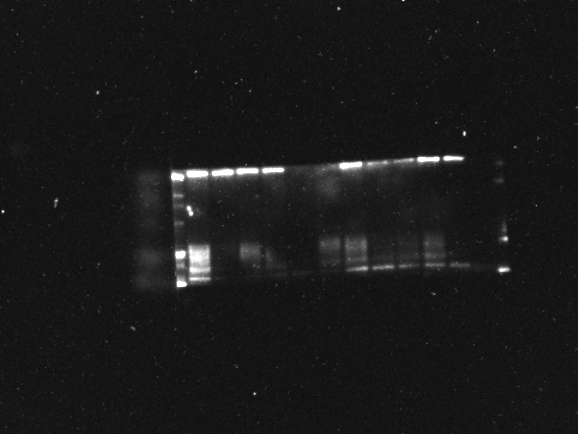

Supplement: Figure 5—figure supplement 4—source data 1. — Labelled (.pdf) and raw (folder) blot images showed in panels B and D are also included. [file elife-79840-fig5-figsupp4-data1.zip › Figure 5 - supplement Figure 4 - source data/blot Figure 5 - figure supplement S4/Figure 5-S4A/Lane_TGFBR2_IPF-5S4A.tiff]

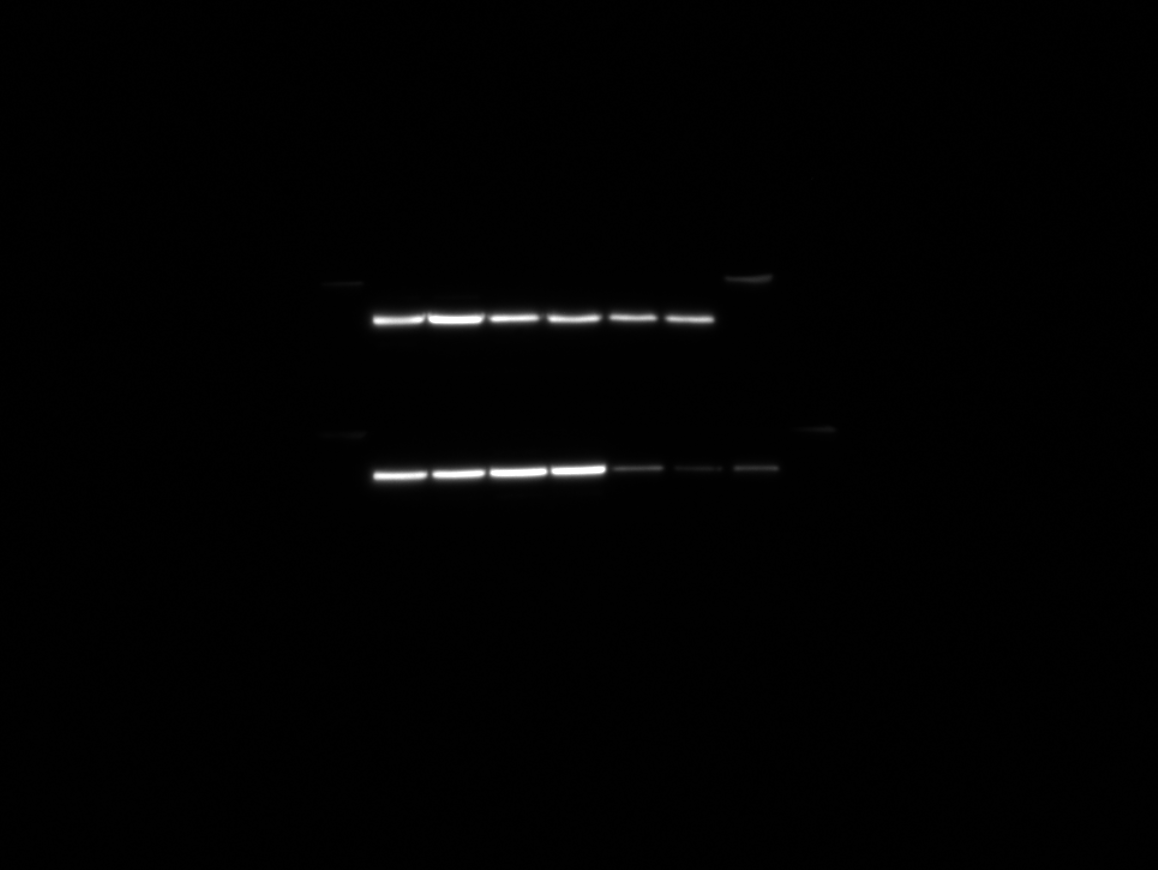

Supplement: Figure 5—figure supplement 4—source data 1. — Labelled (.pdf) and raw (folder) blot images showed in panels B and D are also included. [file elife-79840-fig5-figsupp4-data1.zip › Figure 5 - supplement Figure 4 - source data/blot Figure 5 - figure supplement S4/Figure 5-S4C/Lane-GAPDH-5S4C.tif]

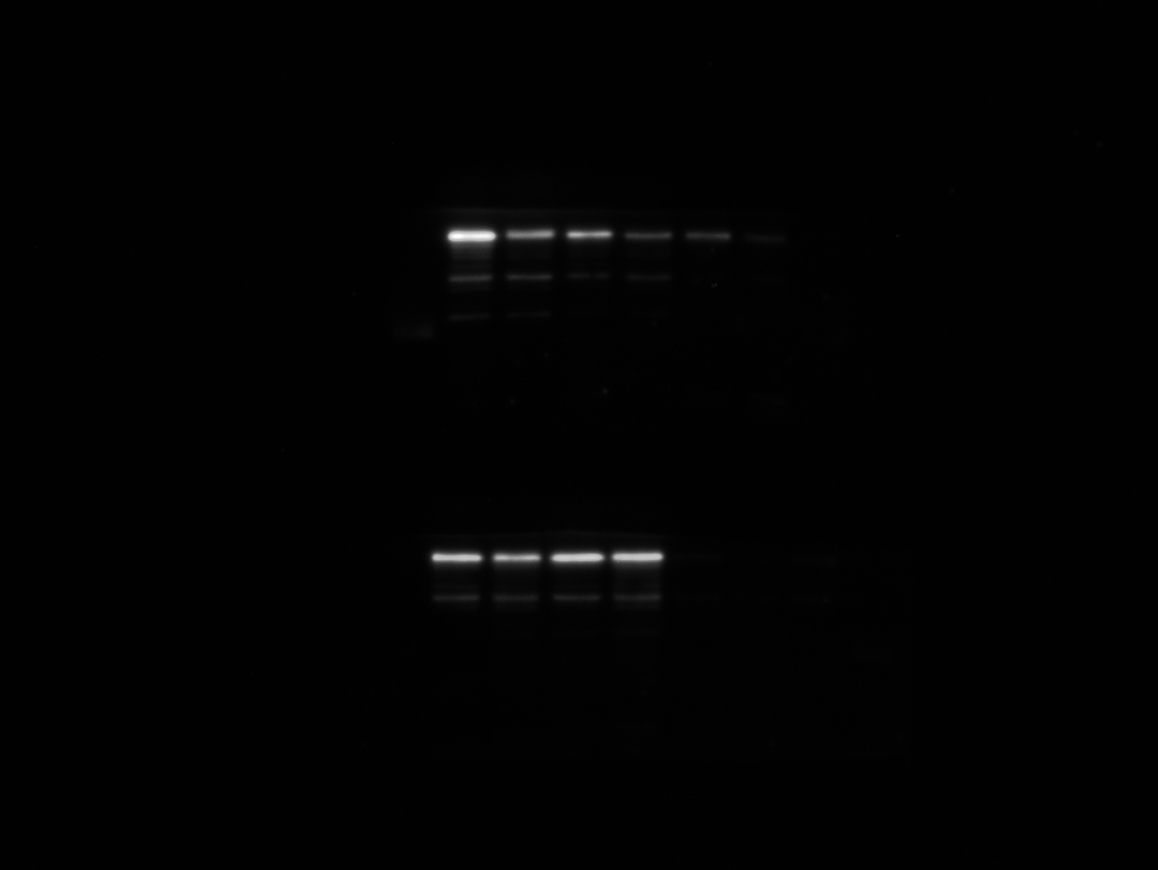

Supplement: Figure 5—figure supplement 4—source data 1. — Labelled (.pdf) and raw (folder) blot images showed in panels B and D are also included. [file elife-79840-fig5-figsupp4-data1.zip › Figure 5 - supplement Figure 4 - source data/blot Figure 5 - figure supplement S4/Figure 5-S4C/Lane-PPM1A-5S4C.tif]

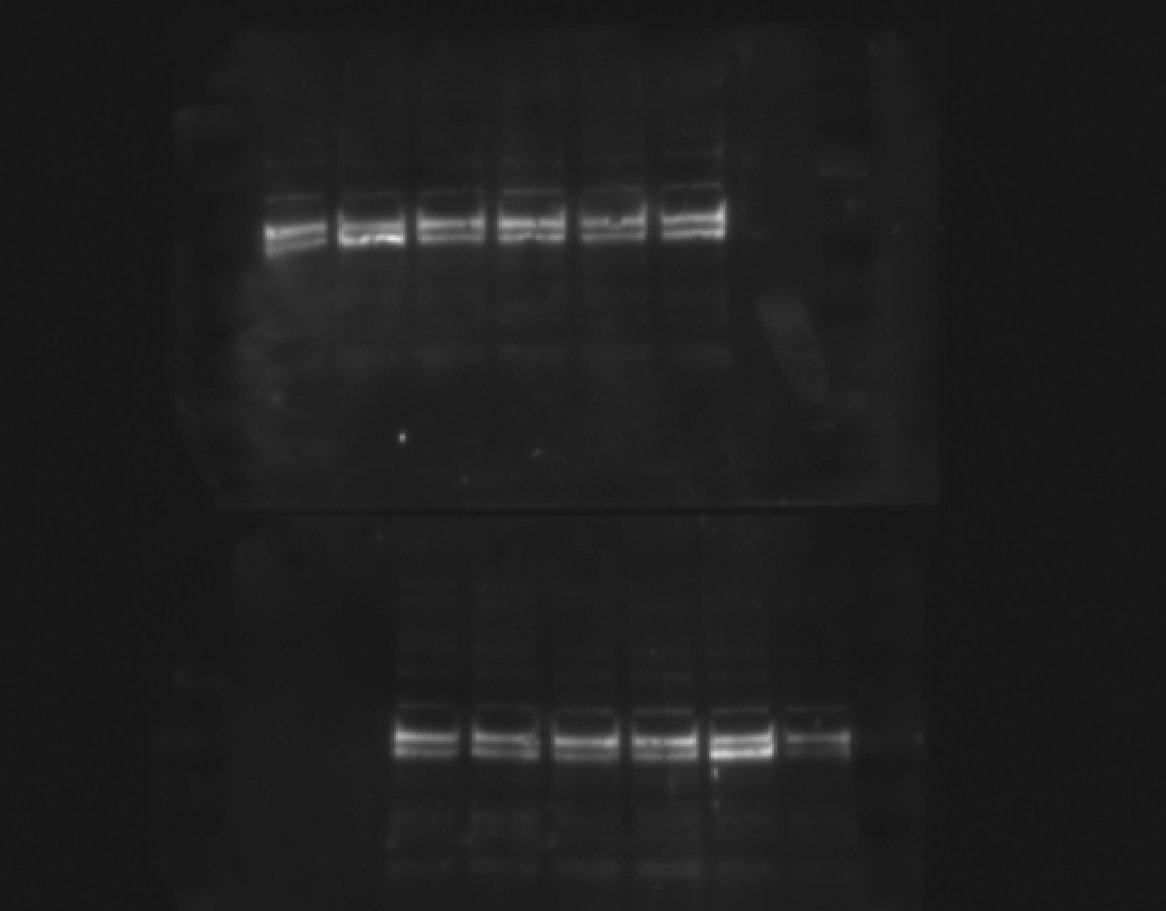

Supplement: Figure 5—figure supplement 4—source data 1. — Labelled (.pdf) and raw (folder) blot images showed in panels B and D are also included. [file elife-79840-fig5-figsupp4-data1.zip › Figure 5 - supplement Figure 4 - source data/blot Figure 5 - figure supplement S4/Figure 5-S4D/Lane-totSMAD2:3-5S4D.tiff]

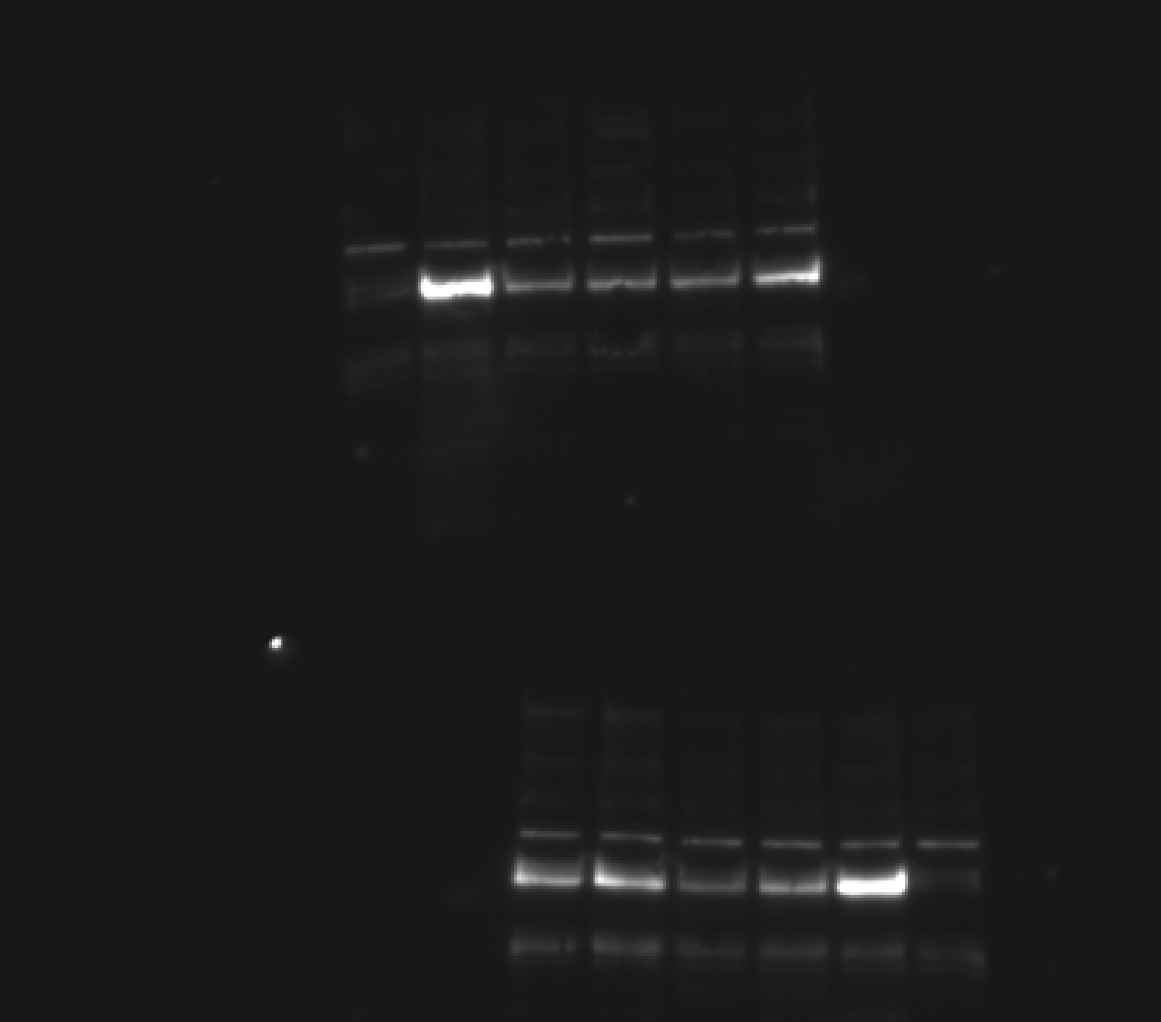

Supplement: Figure 5—figure supplement 4—source data 1. — Labelled (.pdf) and raw (folder) blot images showed in panels B and D are also included. [file elife-79840-fig5-figsupp4-data1.zip › Figure 5 - supplement Figure 4 - source data/blot Figure 5 - figure supplement S4/Figure 5-S4D/Lane-PSMAD3-5S4D.tiff]

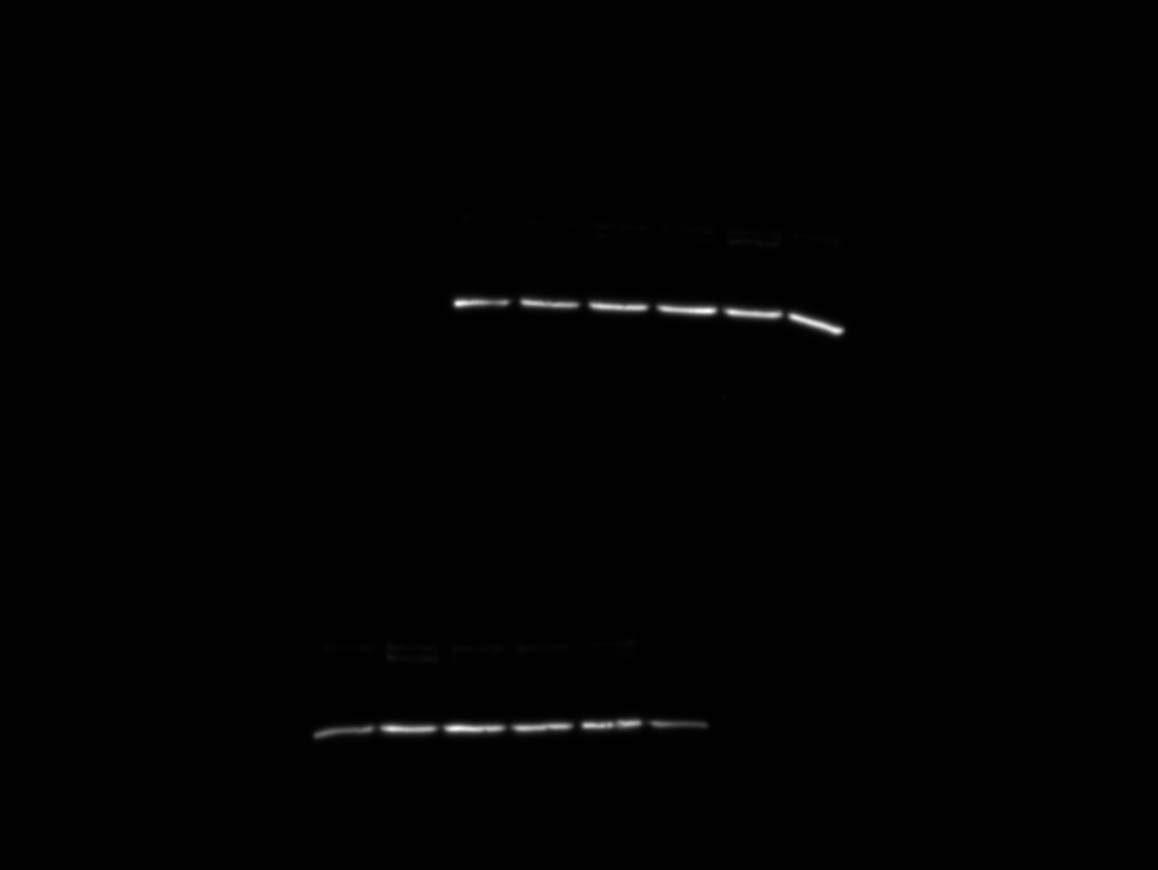

Supplement: Figure 5—figure supplement 4—source data 1. — Labelled (.pdf) and raw (folder) blot images showed in panels B and D are also included. [file elife-79840-fig5-figsupp4-data1.zip › Figure 5 - supplement Figure 4 - source data/blot Figure 5 - figure supplement S4/Figure 5-S4D/Lane-GAPDH-5S4D.tiff]

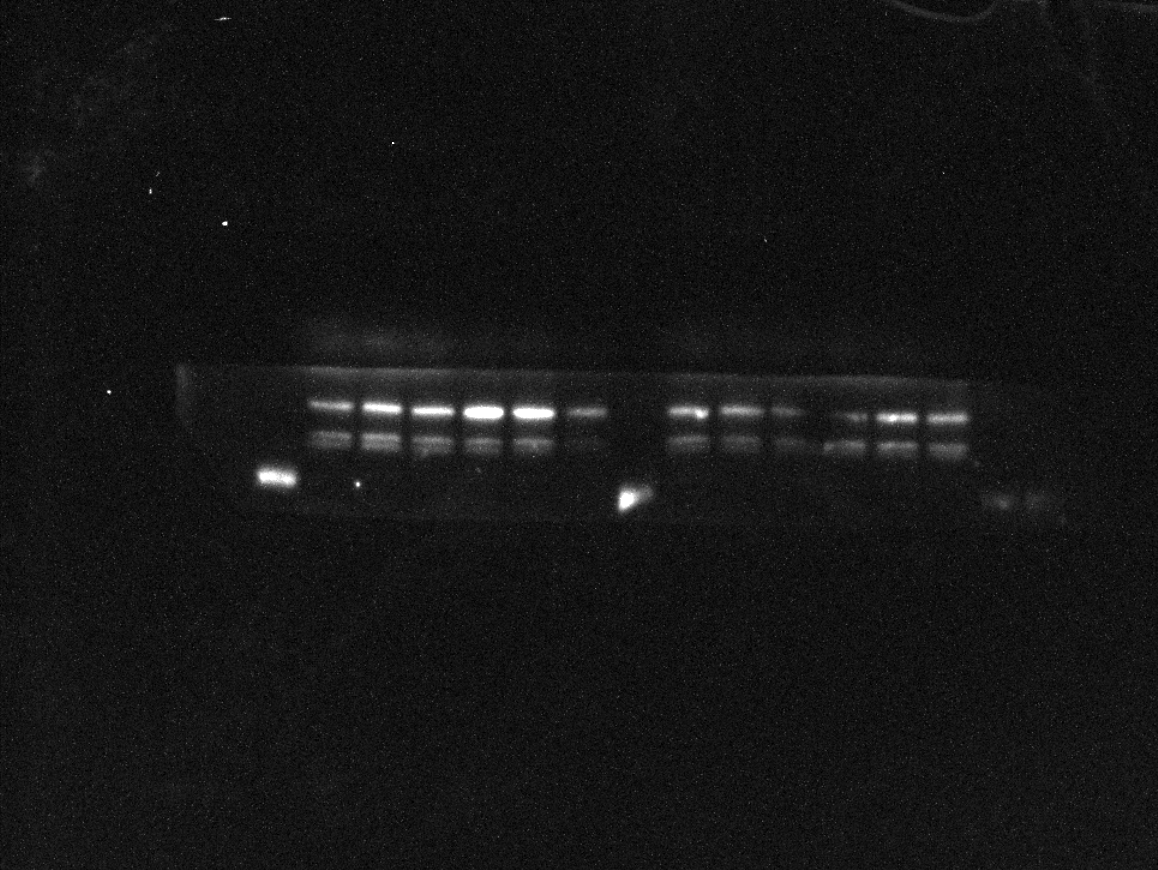

Supplement: Figure 5—figure supplement 4—source data 1. — Labelled (.pdf) and raw (folder) blot images showed in panels B and D are also included. [file elife-79840-fig5-figsupp4-data1.zip › Figure 5 - supplement Figure 4 - source data/blot Figure 5 - figure supplement S4/Figure 5-S4B/Lane-PPM1A-5S4B.tiff]

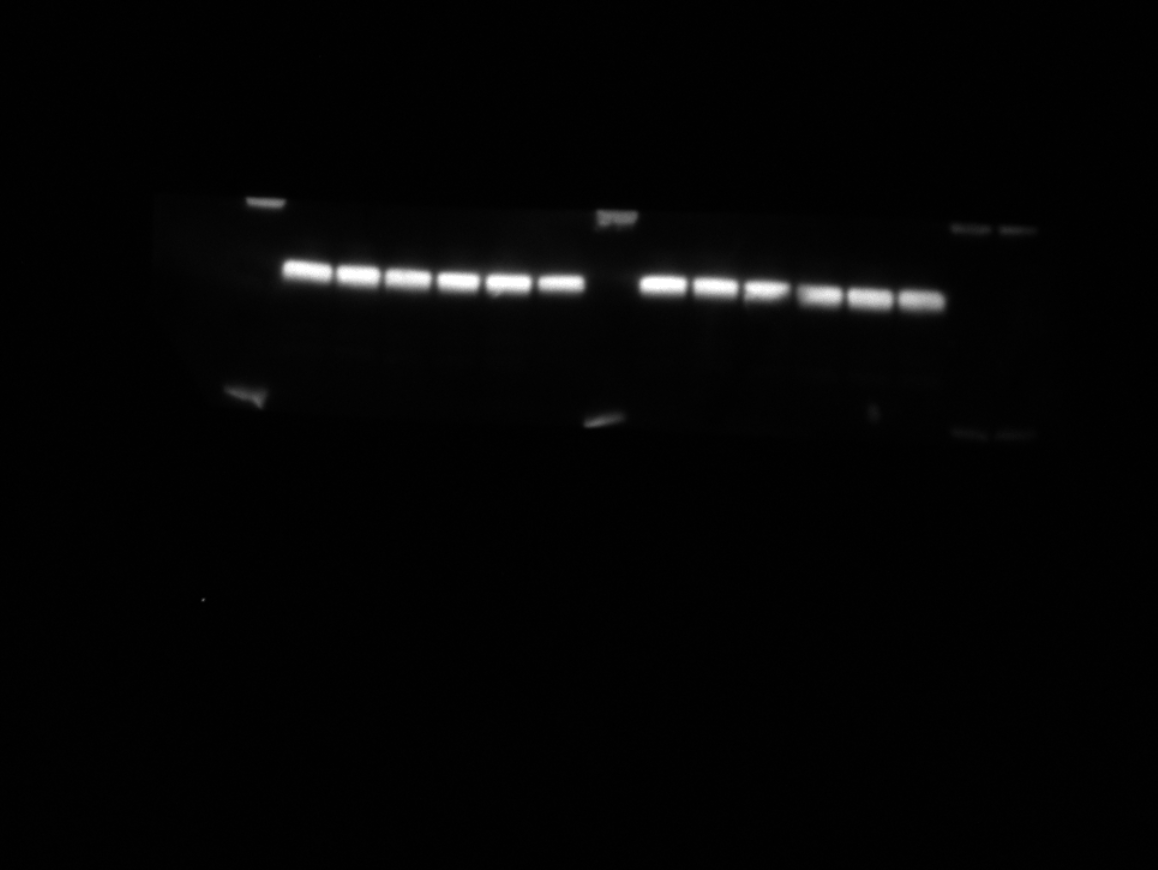

Supplement: Figure 5—figure supplement 4—source data 1. — Labelled (.pdf) and raw (folder) blot images showed in panels B and D are also included. [file elife-79840-fig5-figsupp4-data1.zip › Figure 5 - supplement Figure 4 - source data/blot Figure 5 - figure supplement S4/Figure 5-S4B/Lane-PPM1A-5S4B.tif]

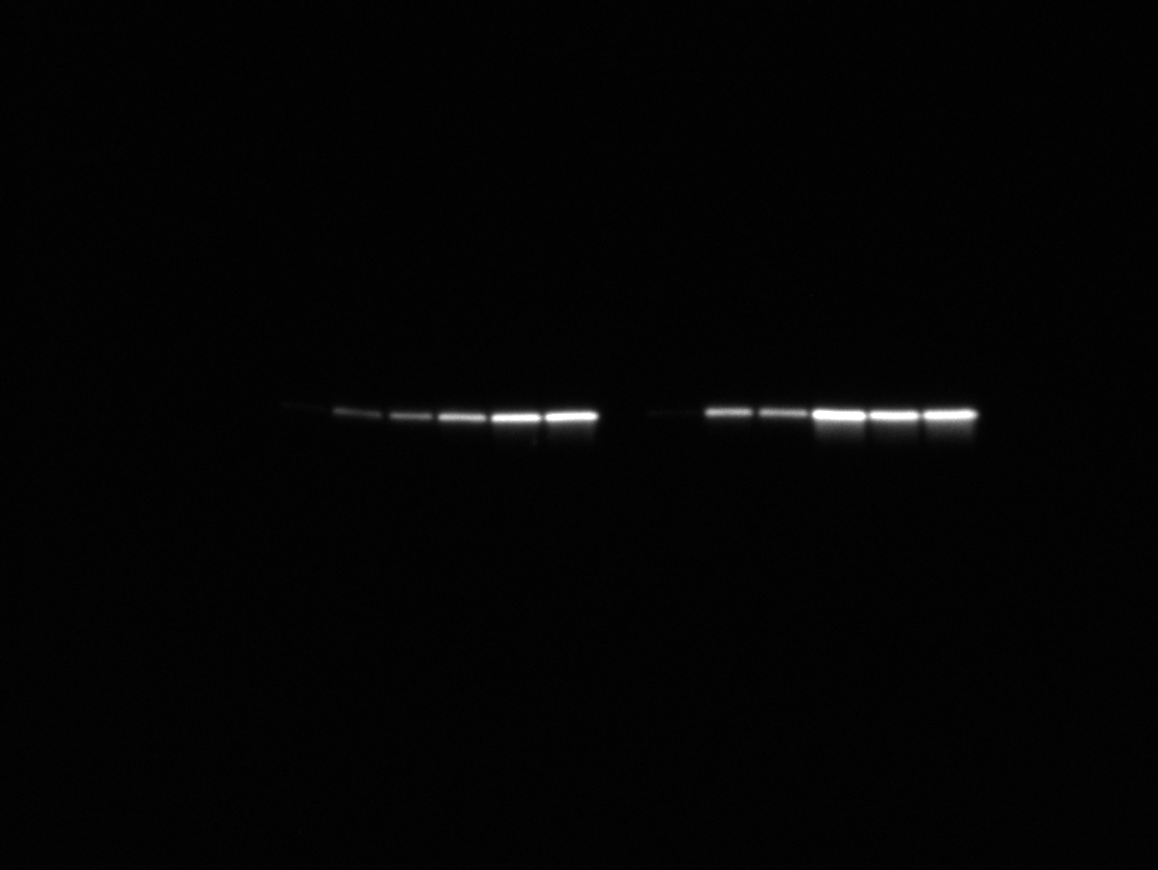

Supplement: Figure 5—figure supplement 5—source data 1. — Labelled (.pdf) and raw (folder) blot images showed in panels A and C are also included. [file elife-79840-fig5-figsupp5-data1.zip › Figure 5 - supplement Figure 5 - source data/blot Figure 5 - figure supplement S5/Figure 5-S5C/Lane-ACTA2-5S5C.tif]

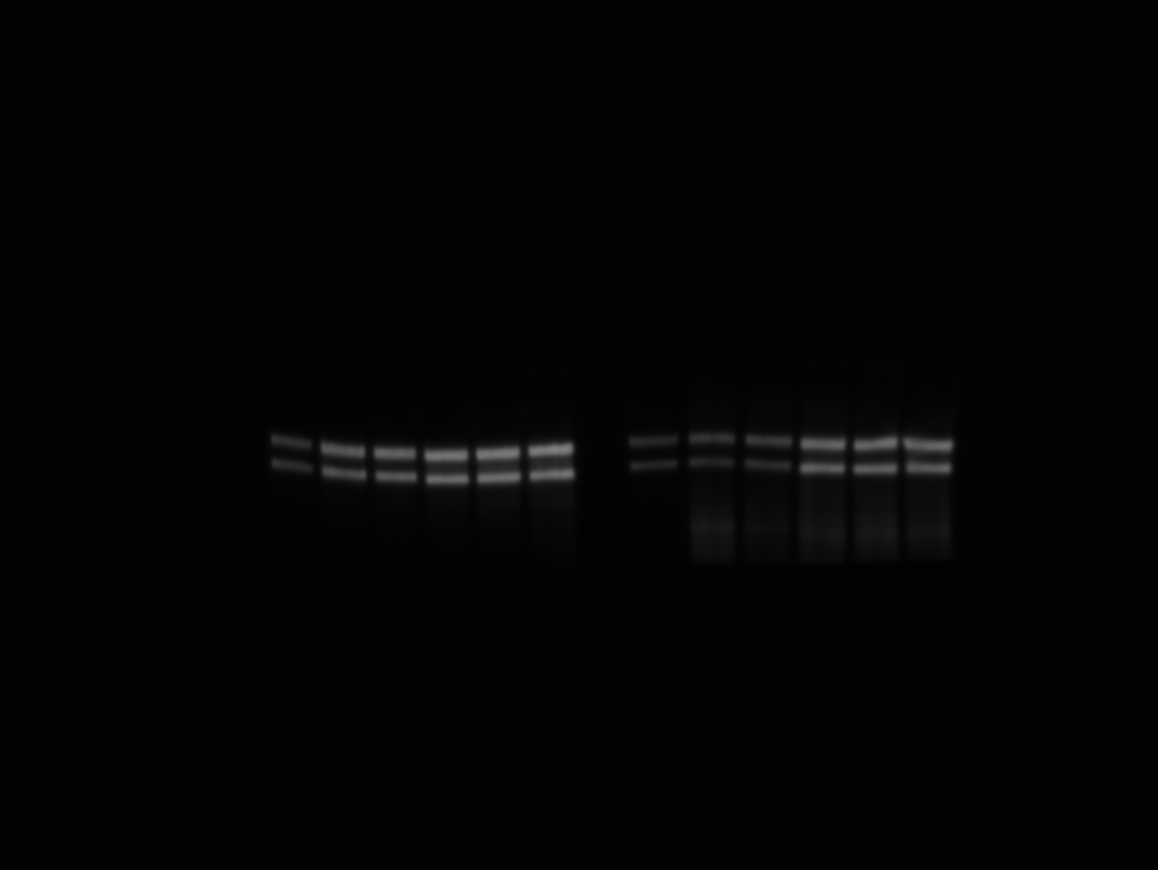

Supplement: Figure 5—figure supplement 5—source data 1. — Labelled (.pdf) and raw (folder) blot images showed in panels A and C are also included. [file elife-79840-fig5-figsupp5-data1.zip › Figure 5 - supplement Figure 5 - source data/blot Figure 5 - figure supplement S5/Figure 5-S5C/Lane-COL1-5S5C.tif]

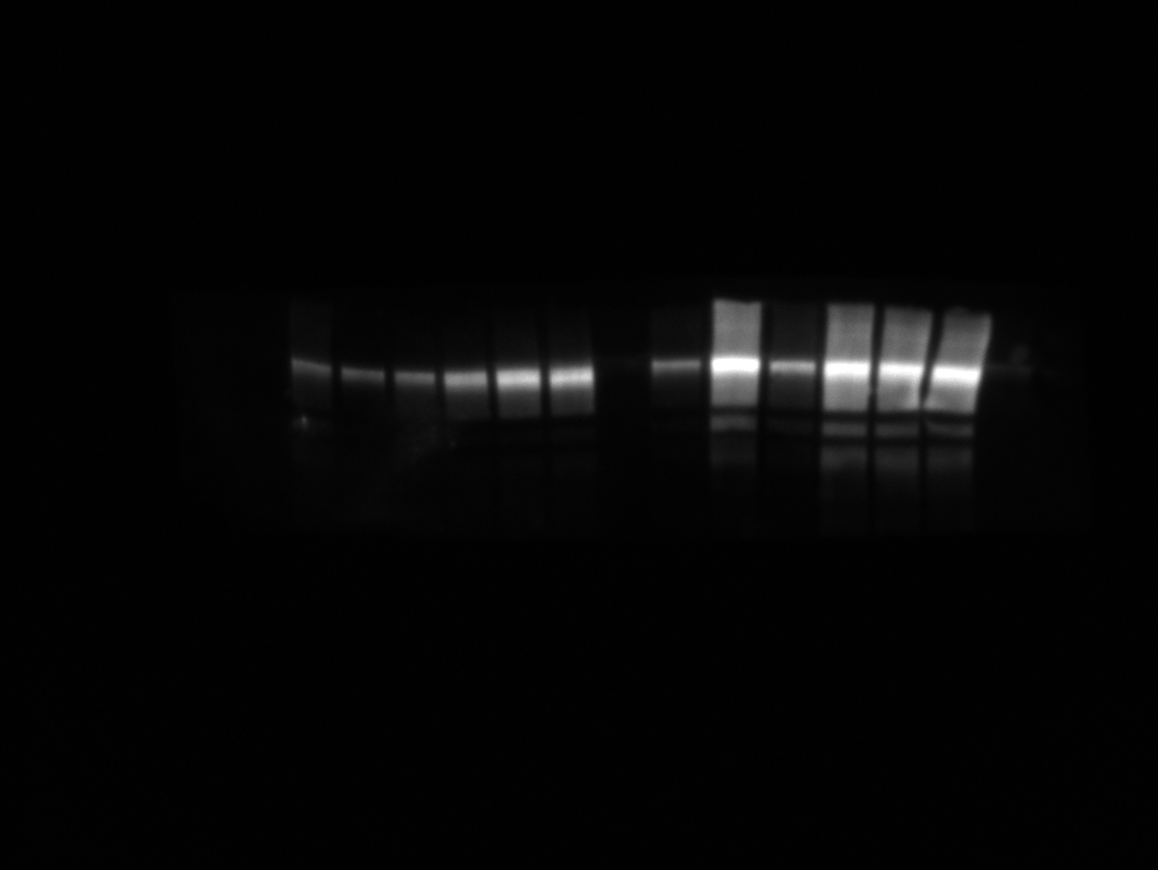

Supplement: Figure 5—figure supplement 5—source data 1. — Labelled (.pdf) and raw (folder) blot images showed in panels A and C are also included. [file elife-79840-fig5-figsupp5-data1.zip › Figure 5 - supplement Figure 5 - source data/blot Figure 5 - figure supplement S5/Figure 5-S5C/Lane-FN1-5S5C.tif]

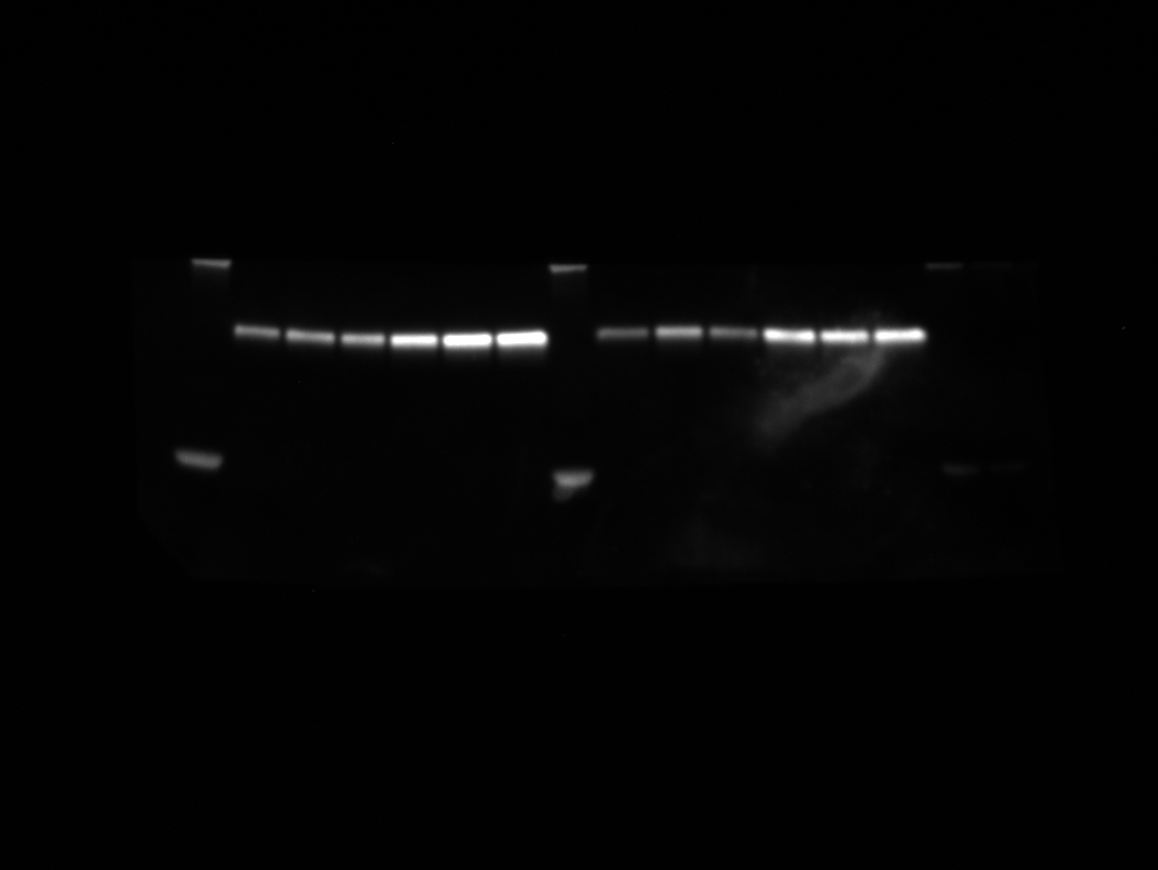

Supplement: Figure 5—figure supplement 5—source data 1. — Labelled (.pdf) and raw (folder) blot images showed in panels A and C are also included. [file elife-79840-fig5-figsupp5-data1.zip › Figure 5 - supplement Figure 5 - source data/blot Figure 5 - figure supplement S5/Figure 5-S5C/Lane-bTUB-5S5C.tif]

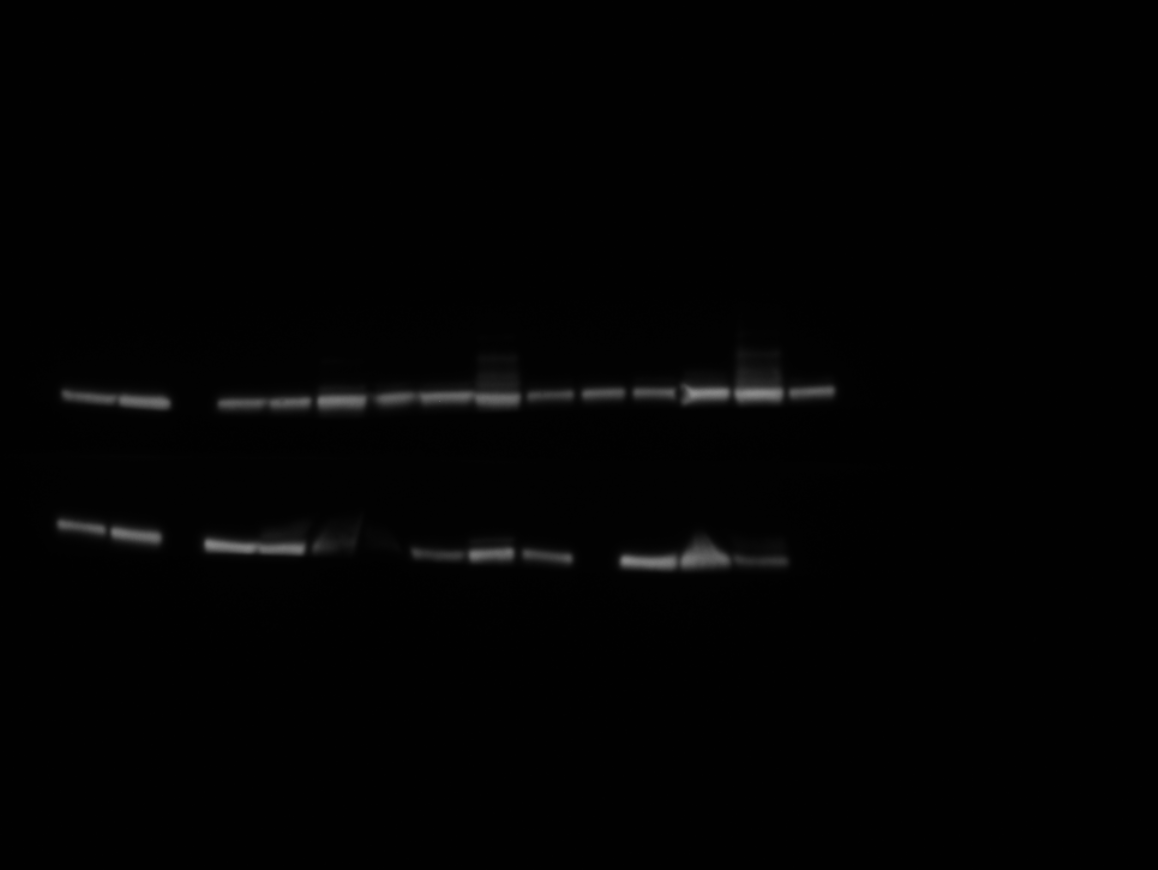

Supplement: Figure 5—figure supplement 5—source data 1. — Labelled (.pdf) and raw (folder) blot images showed in panels A and C are also included. [file elife-79840-fig5-figsupp5-data1.zip › Figure 5 - supplement Figure 5 - source data/blot Figure 5 - figure supplement S5/Figure 5-S5A/Lane-TUB-5S5A.tif]

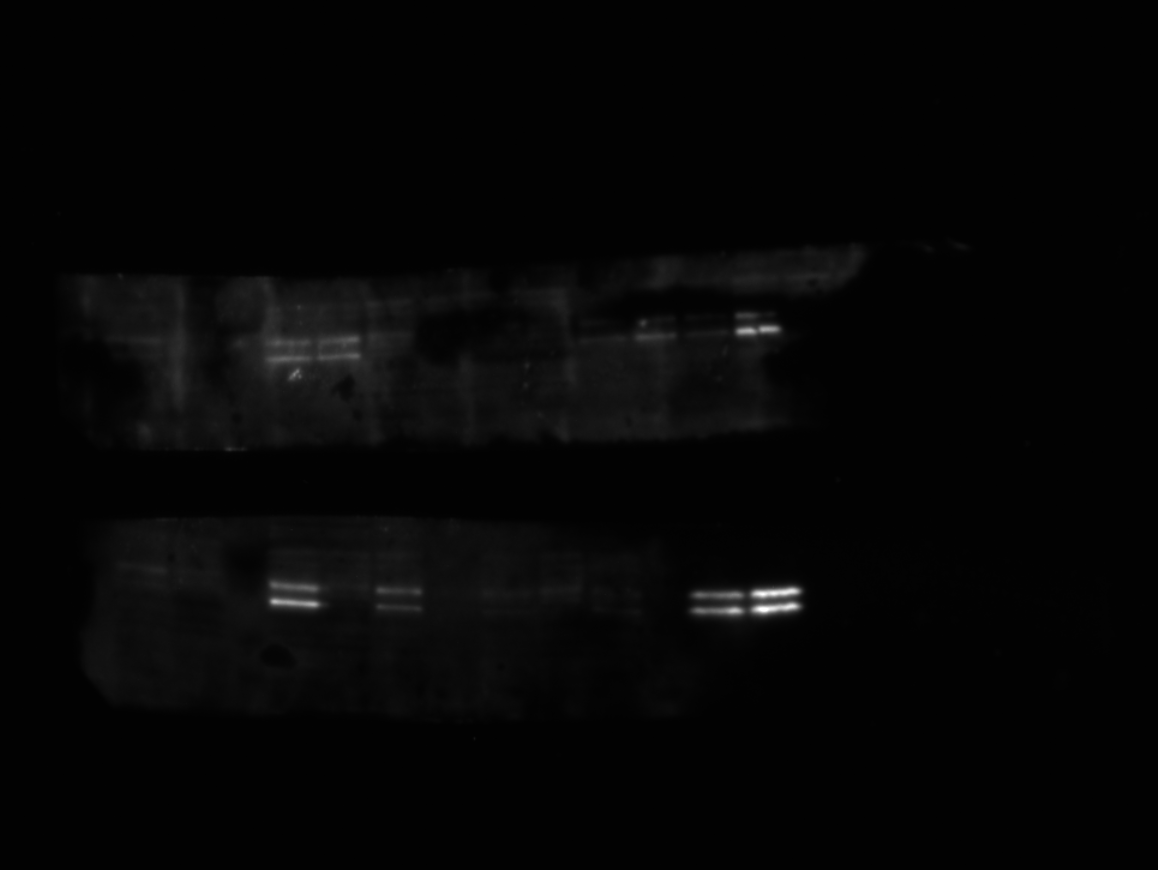

Supplement: Figure 5—figure supplement 5—source data 1. — Labelled (.pdf) and raw (folder) blot images showed in panels A and C are also included. [file elife-79840-fig5-figsupp5-data1.zip › Figure 5 - supplement Figure 5 - source data/blot Figure 5 - figure supplement S5/Figure 5-S5A/Lane-PRRX1-5S5A.tif]

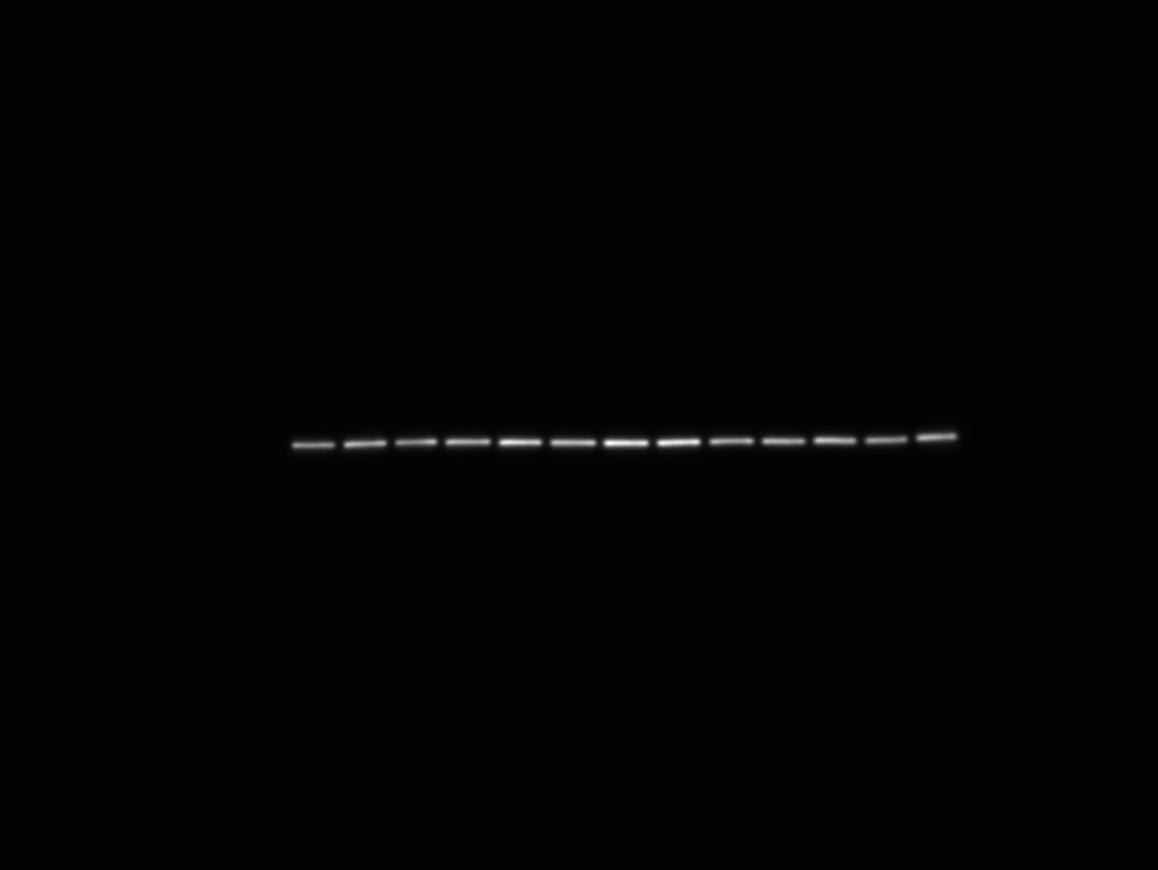

Supplement: Figure 6—source data 1. — Labelled (.pdf) and raw (folder) blot images showed in panel B are also included. [file elife-79840-fig6-data1.zip › Figure 6 - source data/ Blot Figure 6B/Lane-TUB-6B.tif]

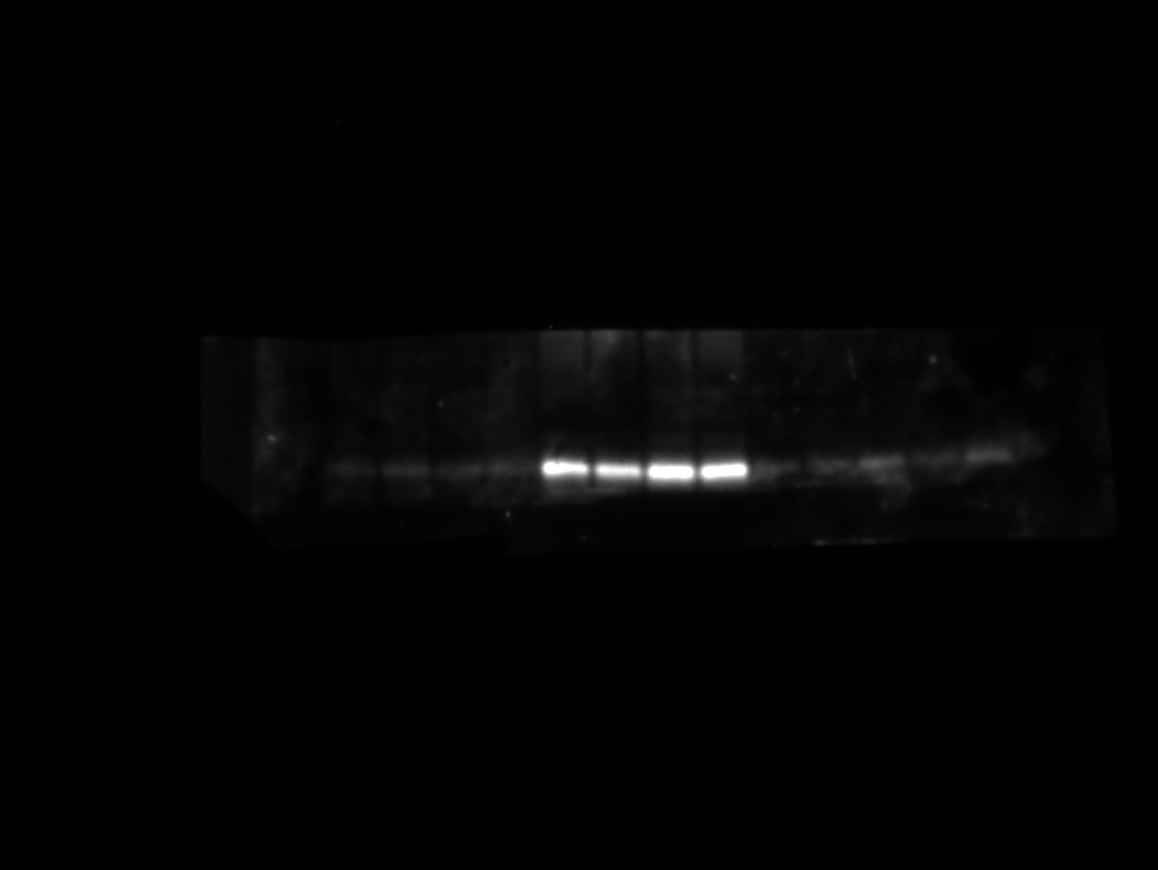

Supplement: Figure 6—source data 1. — Labelled (.pdf) and raw (folder) blot images showed in panel B are also included. [file elife-79840-fig6-data1.zip › Figure 6 - source data/ Blot Figure 6B/Lane-PRRX1-6B.tif]

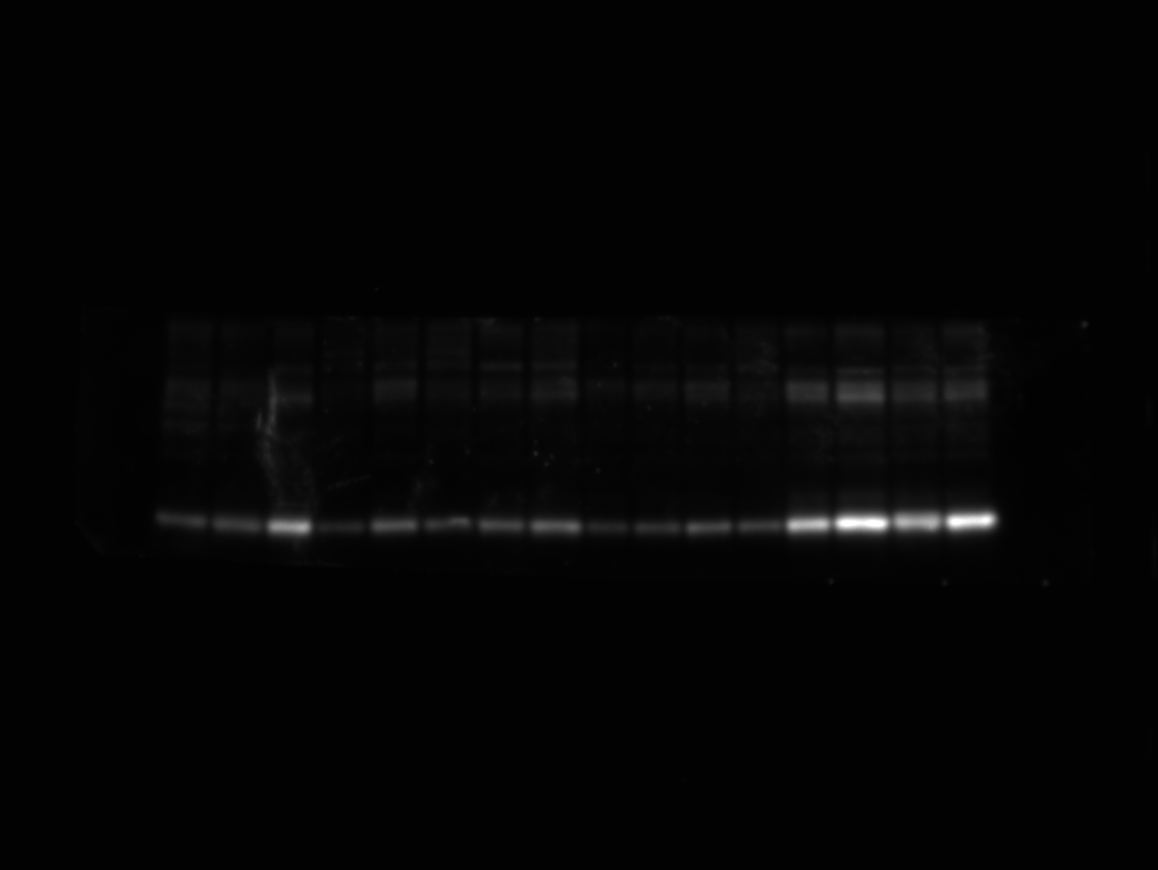

Supplement: Figure 6—figure supplement 1—source data 1. — Labelled (.pdf) and raw (folder) blot images showed in panel B are also included. [file elife-79840-fig6-figsupp1-data1.zip › Figure 6 - supplement figure 1 - data source/Blot Figure 6 - figure supplement S1/Figure 6-S1B/Lane-PRRX1-6S1B-up.tif]

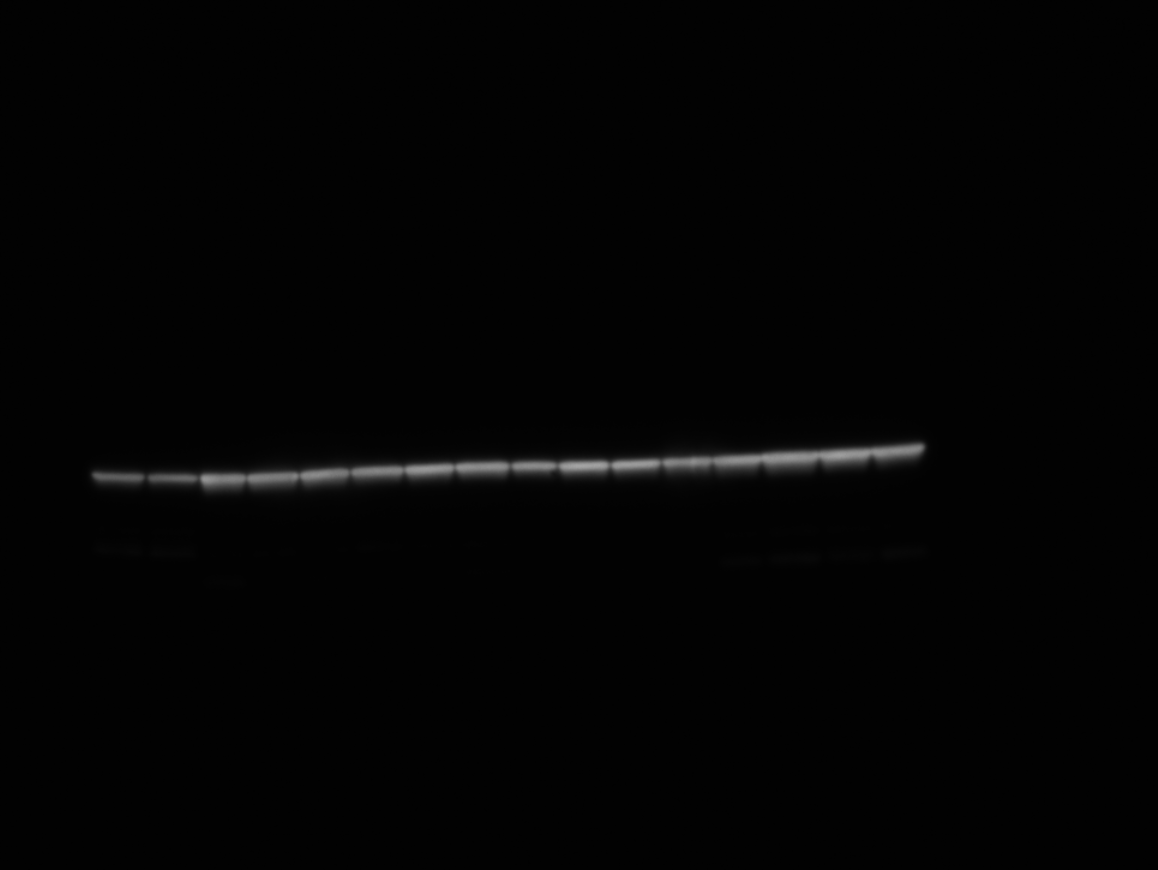

Supplement: Figure 6—figure supplement 1—source data 1. — Labelled (.pdf) and raw (folder) blot images showed in panel B are also included. [file elife-79840-fig6-figsupp1-data1.zip › Figure 6 - supplement figure 1 - data source/Blot Figure 6 - figure supplement S1/Figure 6-S1B/Lane-ACTB-6S1B-up.tif]

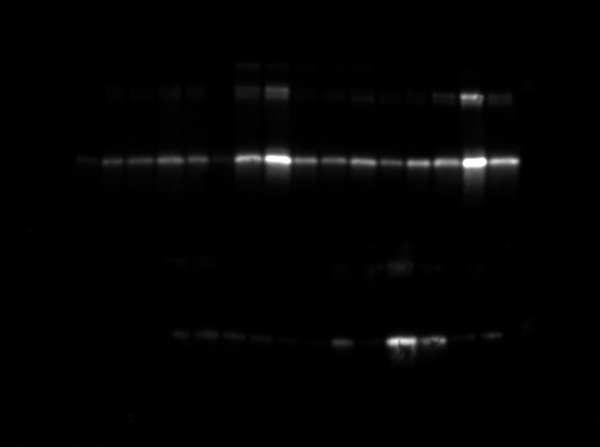

Supplement: Figure 6—figure supplement 1—source data 1. — Labelled (.pdf) and raw (folder) blot images showed in panel B are also included. [file elife-79840-fig6-figsupp1-data1.zip › Figure 6 - supplement figure 1 - data source/Blot Figure 6 - figure supplement S1/Figure 6-S1B/Lane-PRRX1-6S1B-bottom.tiff]

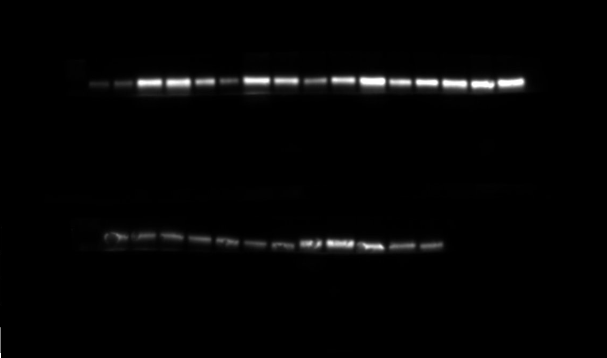

Supplement: Figure 6—figure supplement 1—source data 1. — Labelled (.pdf) and raw (folder) blot images showed in panel B are also included. [file elife-79840-fig6-figsupp1-data1.zip › Figure 6 - supplement figure 1 - data source/Blot Figure 6 - figure supplement S1/Figure 6-S1B/Lane-ACTB-6S1B-bottom.tiff]

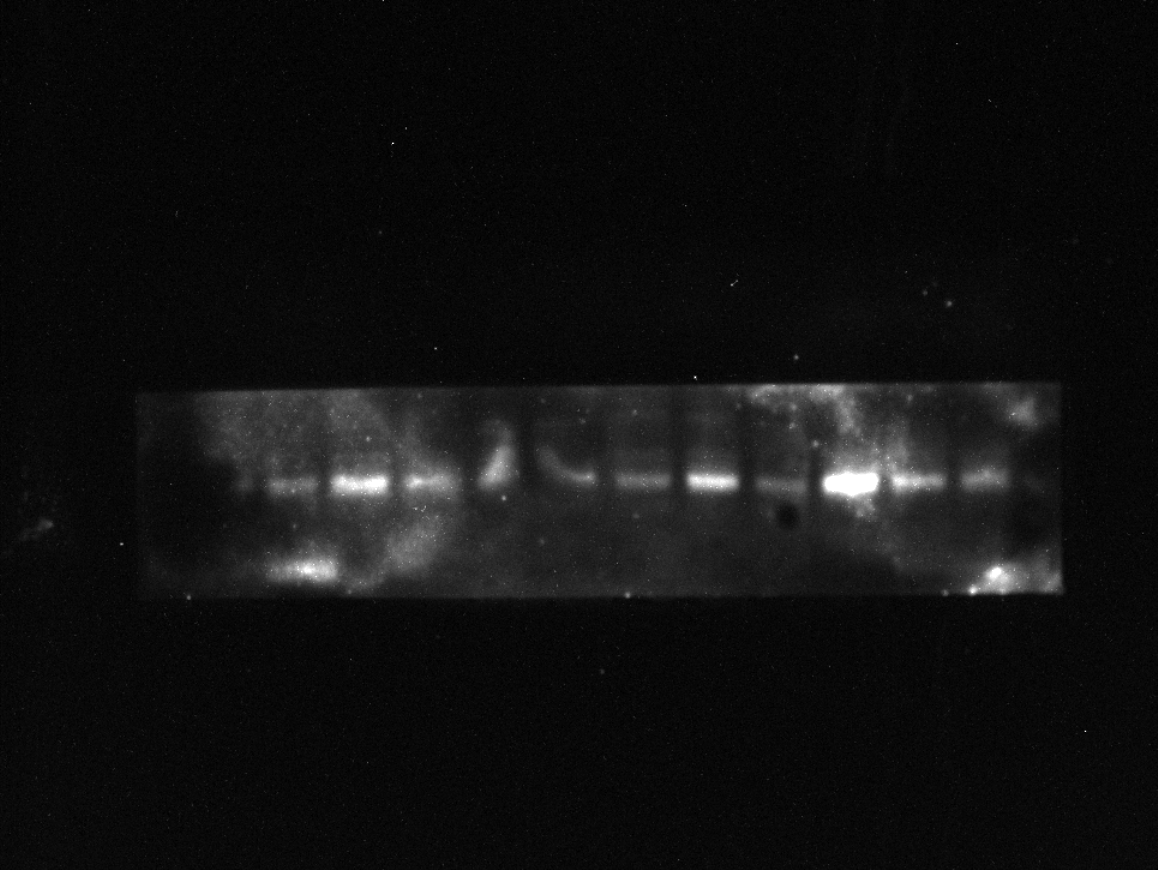

Supplement: Figure 6—figure supplement 2—source data 1. — Labelled (.pdf) and raw (folder) blot images showed in panels B and G are also included. [file elife-79840-fig6-figsupp2-data1.zip › Figure 6 - supplement figure 2 - data source/Blot Figure 6 - figure supplement S2/Figure 6-S2B/Lane-PRRX1-6S2B.tif]

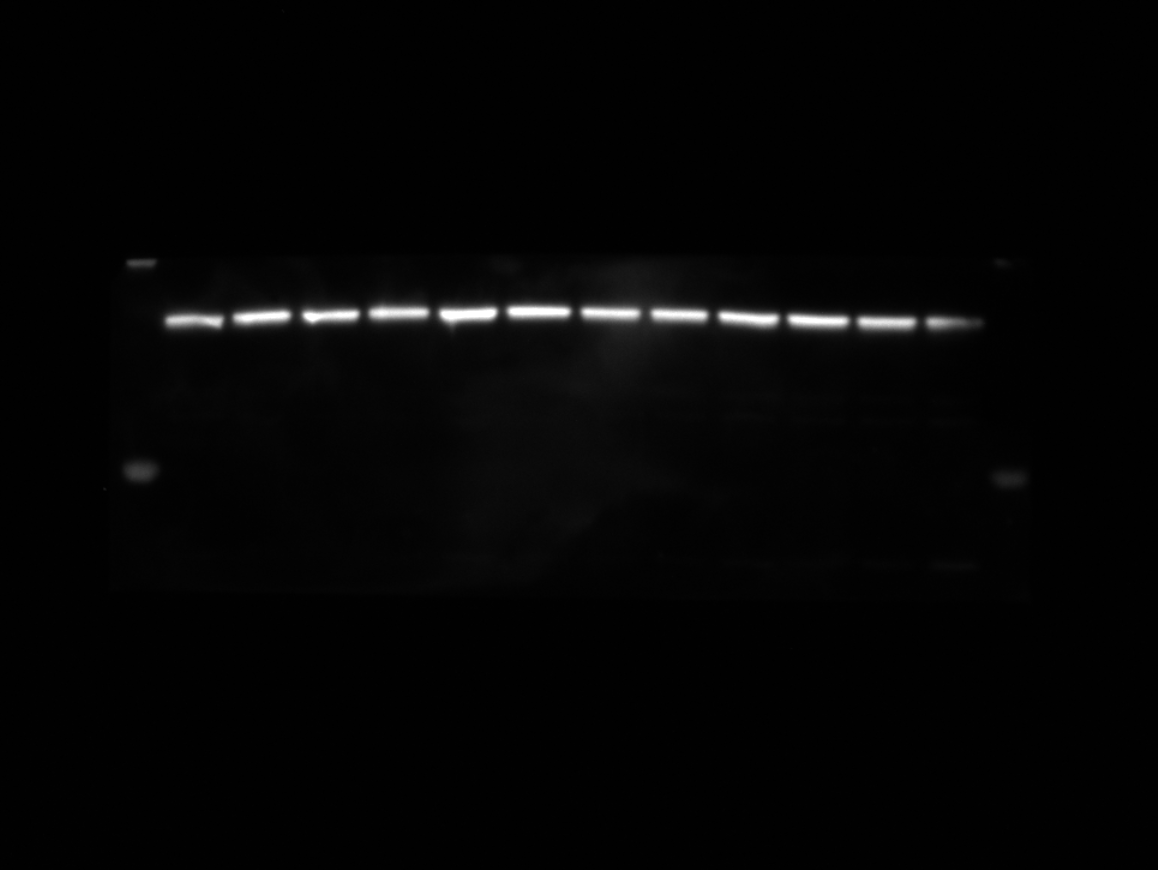

Supplement: Figure 6—figure supplement 2—source data 1. — Labelled (.pdf) and raw (folder) blot images showed in panels B and G are also included. [file elife-79840-fig6-figsupp2-data1.zip › Figure 6 - supplement figure 2 - data source/Blot Figure 6 - figure supplement S2/Figure 6-S2B/Lane-TUB-6S2B.tif]

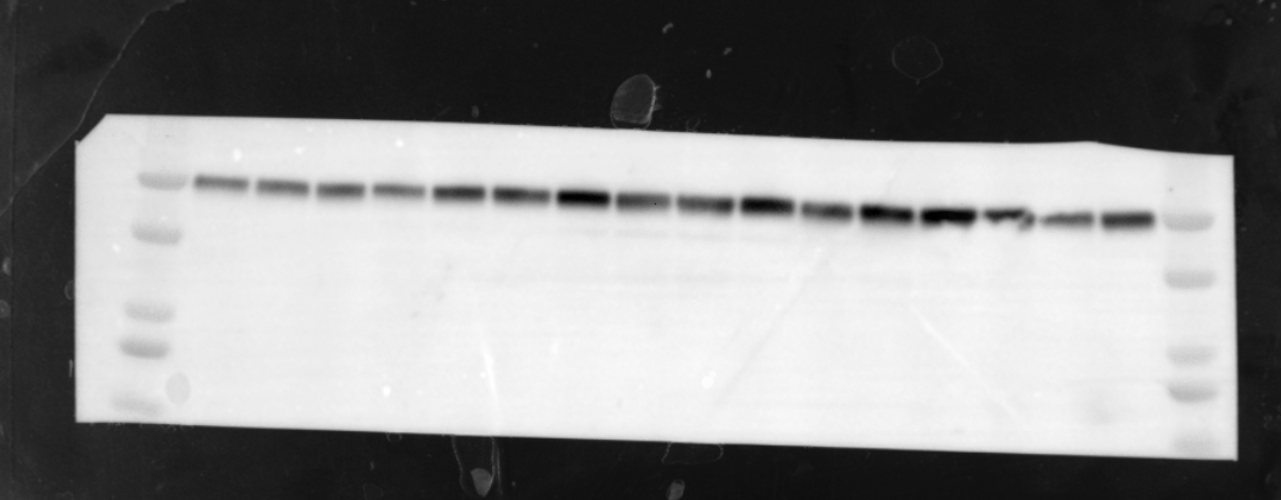

Supplement: Figure 6—figure supplement 2—source data 1. — Labelled (.pdf) and raw (folder) blot images showed in panels B and G are also included. [file elife-79840-fig6-figsupp2-data1.zip › Figure 6 - supplement figure 2 - data source/Blot Figure 6 - figure supplement S2/Figure 6-S2G/Lane-TUB-6S2G.tiff]

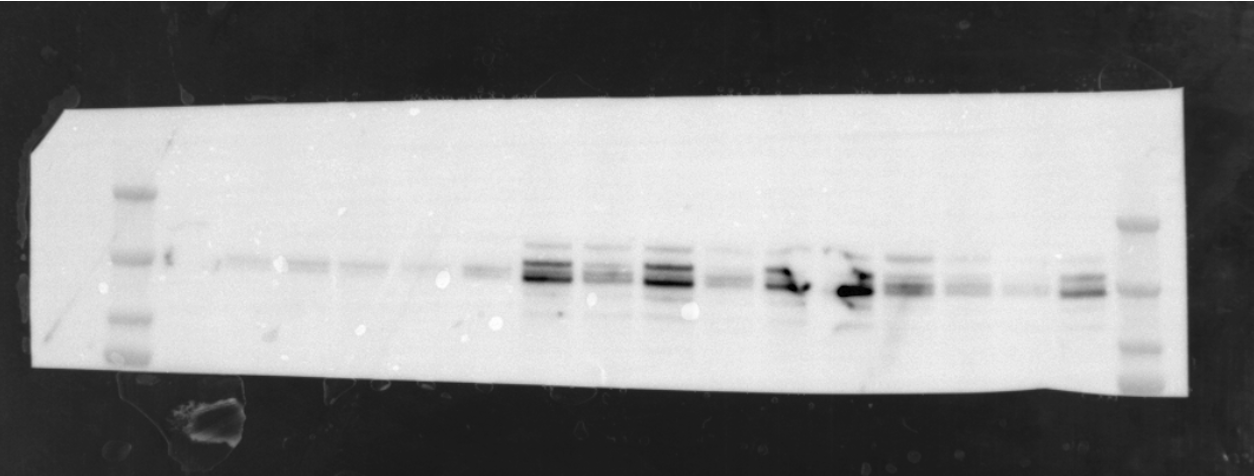

Supplement: Figure 6—figure supplement 2—source data 1. — Labelled (.pdf) and raw (folder) blot images showed in panels B and G are also included. [file elife-79840-fig6-figsupp2-data1.zip › Figure 6 - supplement figure 2 - data source/Blot Figure 6 - figure supplement S2/Figure 6-S2G/Lane-COL1-6S2G.tiff]

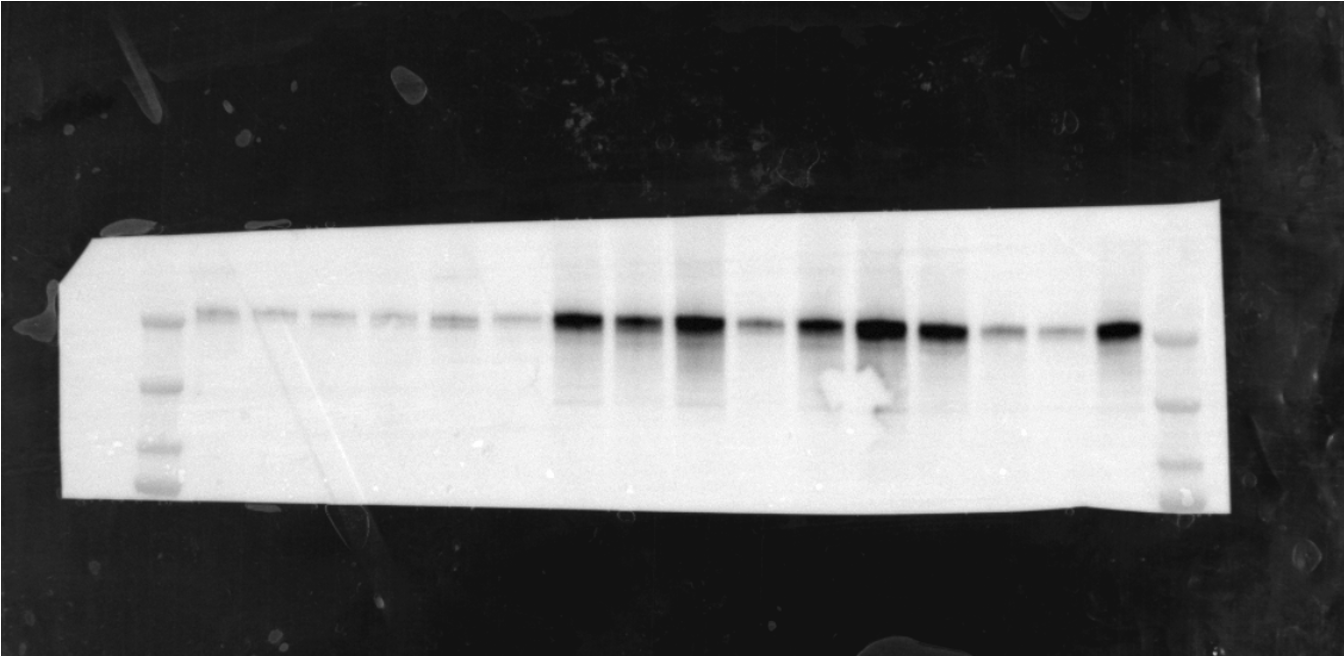

Supplement: Figure 6—figure supplement 2—source data 1. — Labelled (.pdf) and raw (folder) blot images showed in panels B and G are also included. [file elife-79840-fig6-figsupp2-data1.zip › Figure 6 - supplement figure 2 - data source/Blot Figure 6 - figure supplement S2/Figure 6-S2G/Lane-FN1-6S2G.tiff]

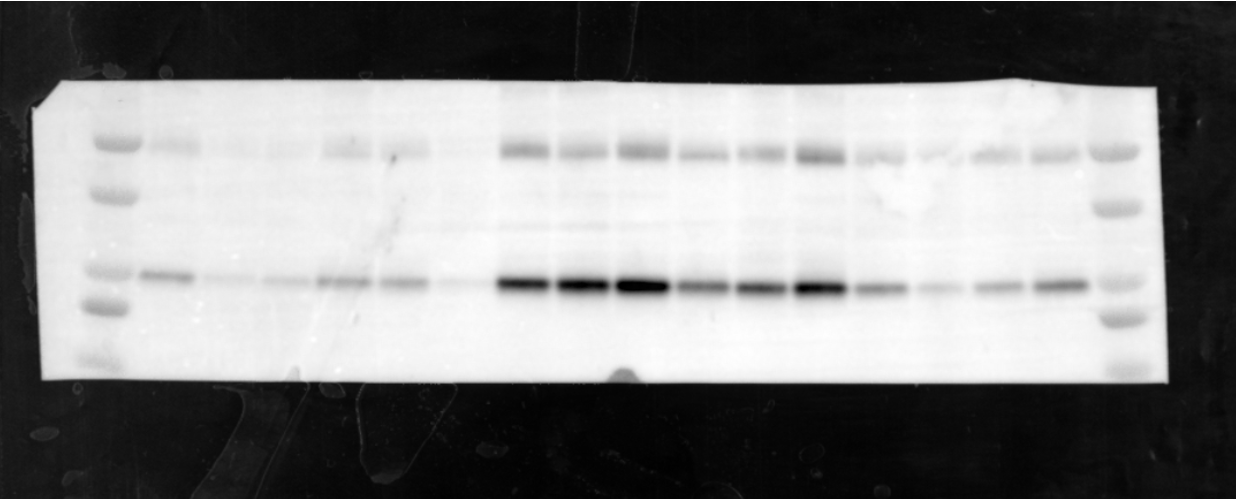

Supplement: Figure 6—figure supplement 2—source data 1. — Labelled (.pdf) and raw (folder) blot images showed in panels B and G are also included. [file elife-79840-fig6-figsupp2-data1.zip › Figure 6 - supplement figure 2 - data source/Blot Figure 6 - figure supplement S2/Figure 6-S2G/Lane-PRRX1-6S2G.tiff]

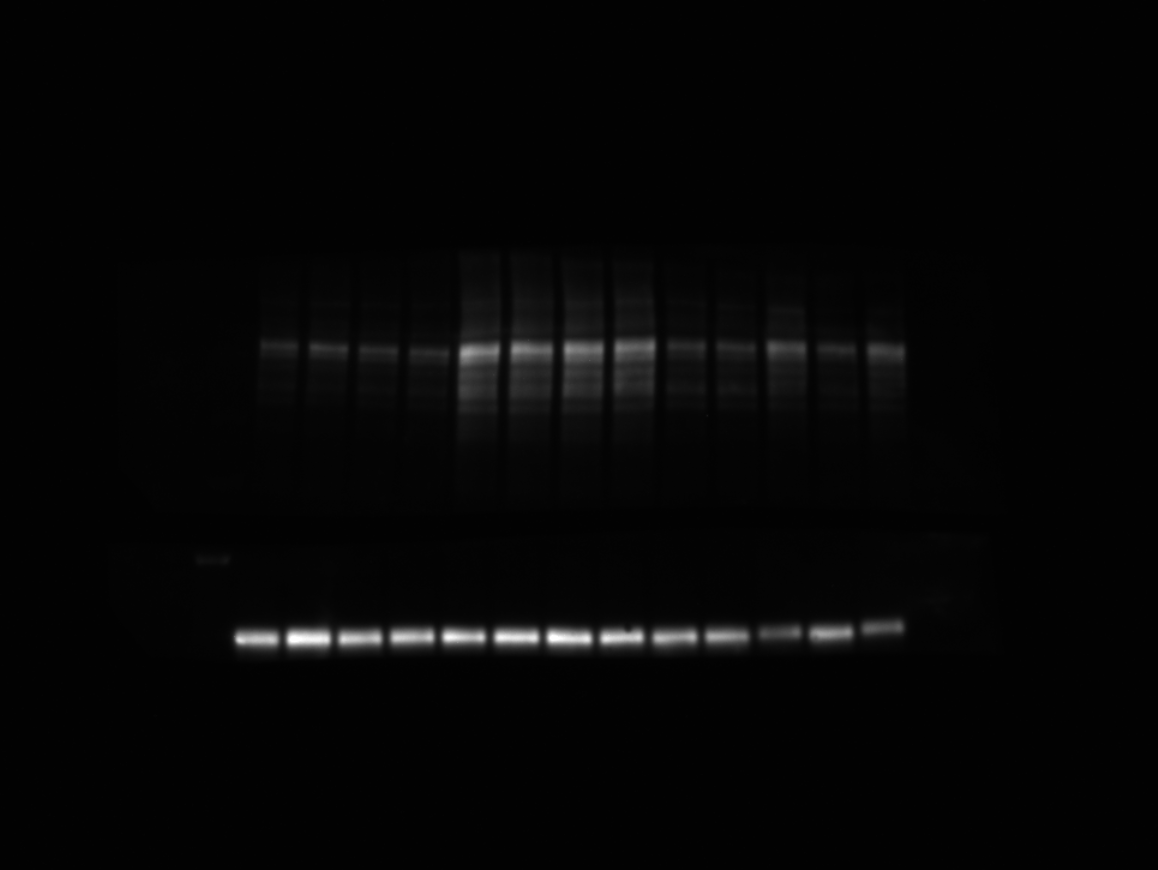

Supplement: Figure 7—source data 1. — Labelled (.pdf) and raw (folder) blot images showed in panel B are also included. [file elife-79840-fig7-data1.zip › Figure 7 - source data/ Blot Figure 7B/Lane-FN1-7B.tif]

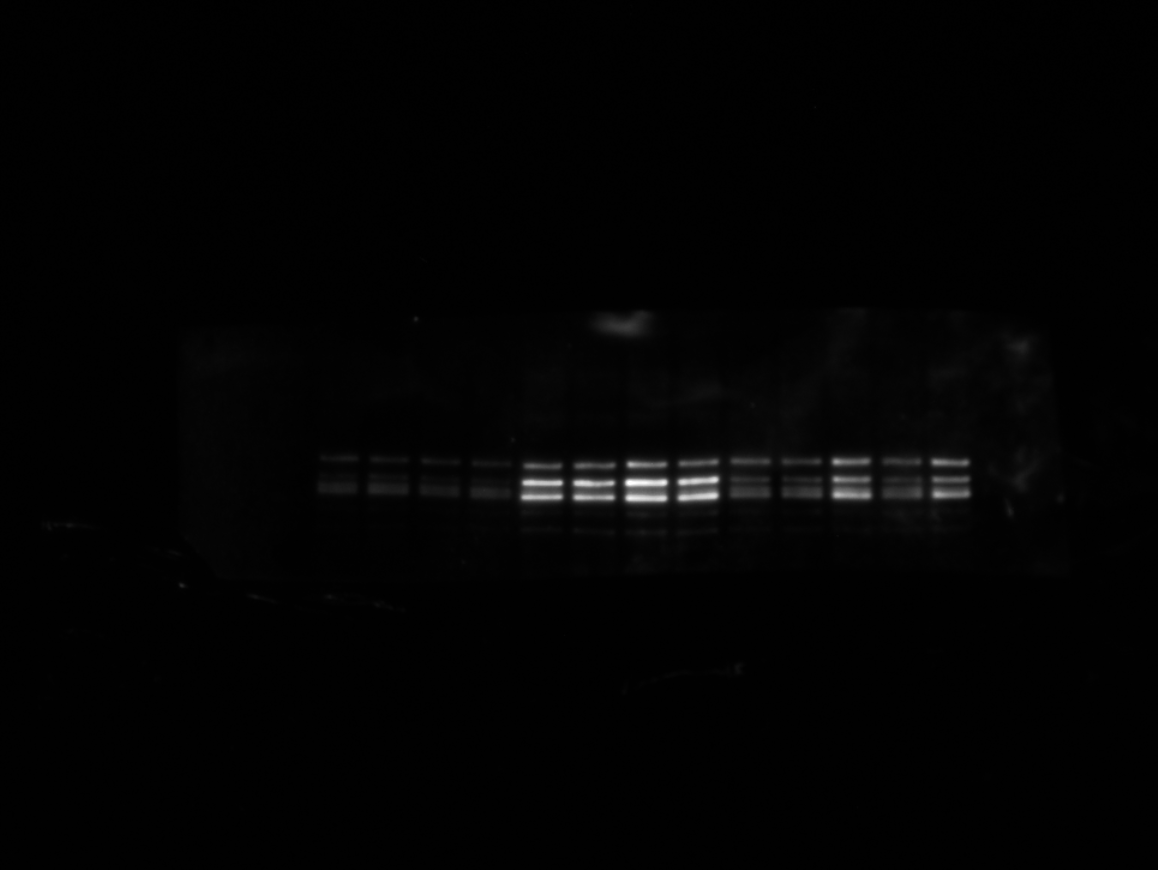

Supplement: Figure 7—source data 1. — Labelled (.pdf) and raw (folder) blot images showed in panel B are also included. [file elife-79840-fig7-data1.zip › Figure 7 - source data/ Blot Figure 7B/Lane-COL1-7B.tif]

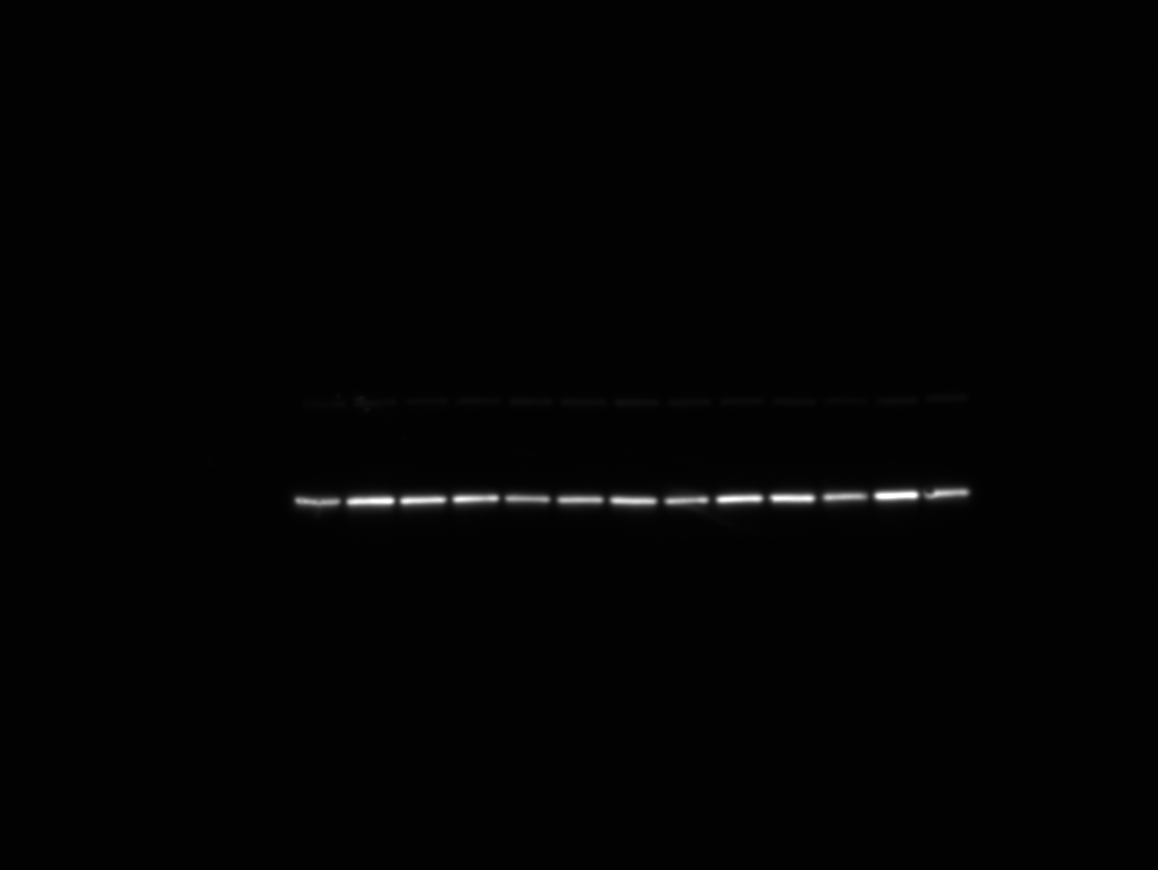

Supplement: Figure 7—source data 1. — Labelled (.pdf) and raw (folder) blot images showed in panel B are also included. [file elife-79840-fig7-data1.zip › Figure 7 - source data/ Blot Figure 7B/Lane-ACTA2-7B.tif]

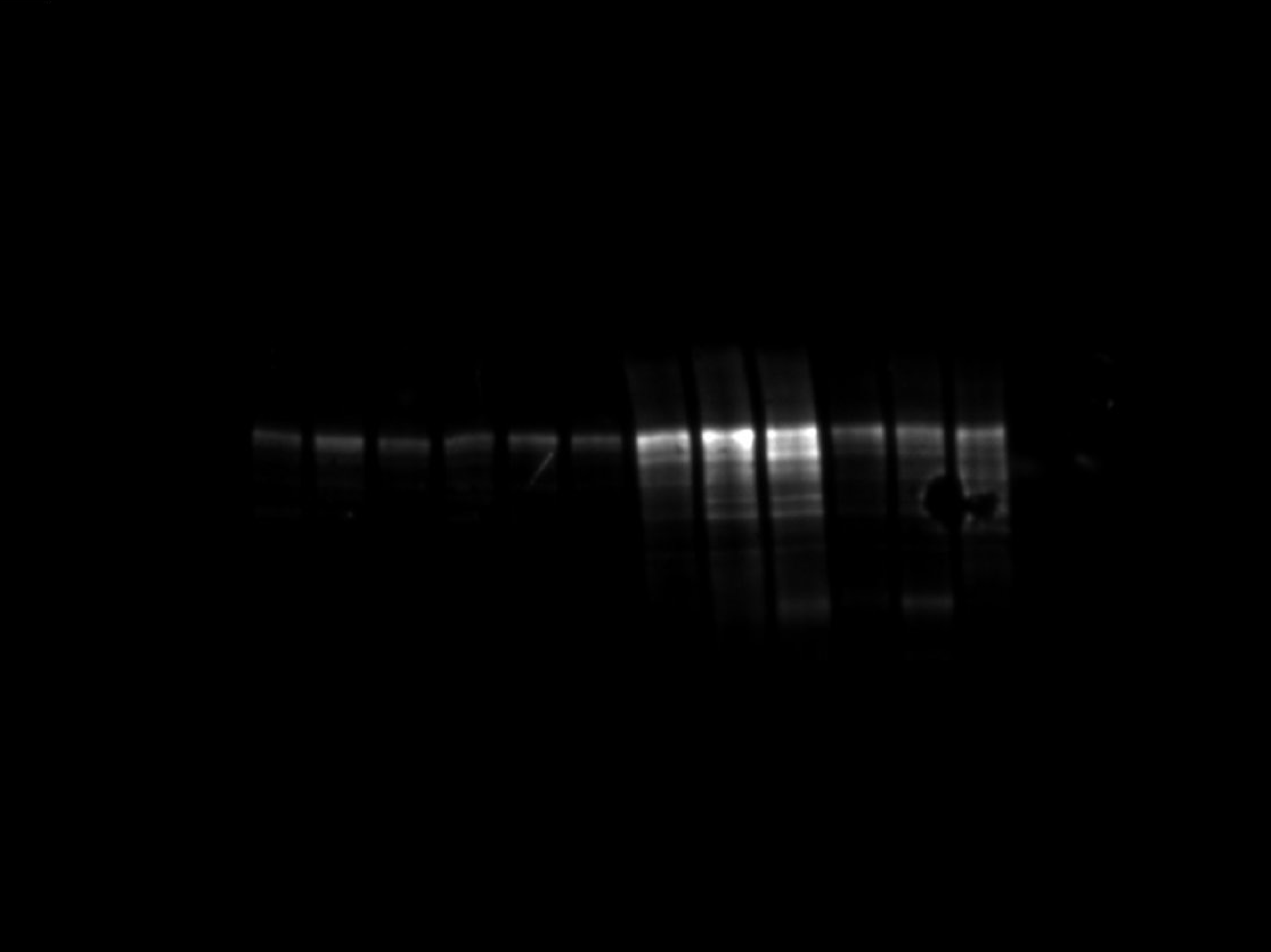

Supplement: Figure 7—figure supplement 1—source data 1. — Labelled (.pdf) and raw (folder) blot images showed in panel F are also included. [file elife-79840-fig7-figsupp1-data1.zip › Figure 7 - supplement figure 1 - data source/Blot Figure 7 - figure supplement S1/Figure 7-S1F/Lane-FN1-panel-7S1F.tiff]

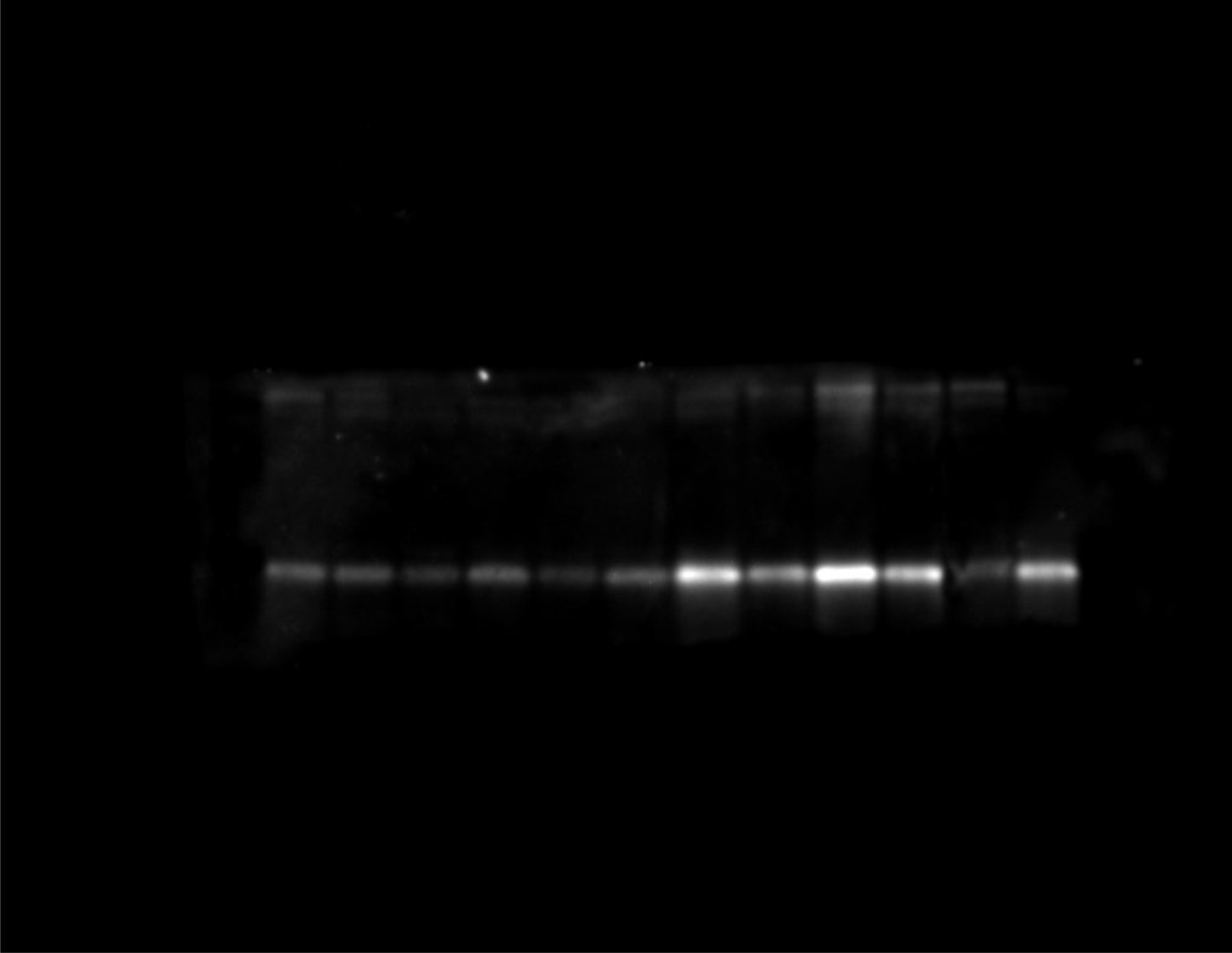

Supplement: Figure 7—figure supplement 1—source data 1. — Labelled (.pdf) and raw (folder) blot images showed in panel F are also included. [file elife-79840-fig7-figsupp1-data1.zip › Figure 7 - supplement figure 1 - data source/Blot Figure 7 - figure supplement S1/Figure 7-S1F/Lane-PRRX1-panel-7S1F.tiff]

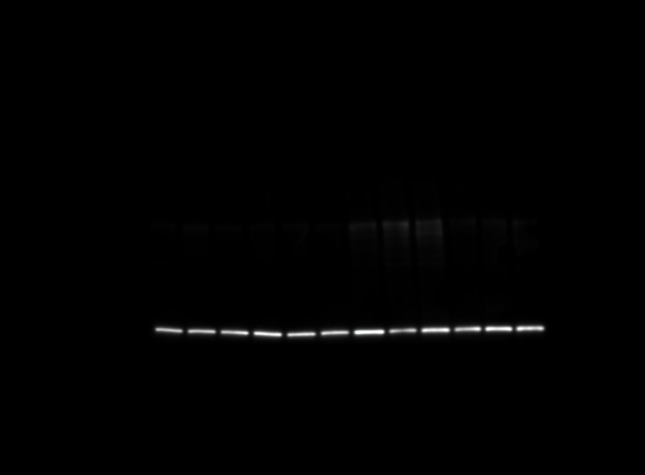

Supplement: Figure 7—figure supplement 1—source data 1. — Labelled (.pdf) and raw (folder) blot images showed in panel F are also included. [file elife-79840-fig7-figsupp1-data1.zip › Figure 7 - supplement figure 1 - data source/Blot Figure 7 - figure supplement S1/Figure 7-S1F/Lane-HSC70-panel-7S1F.tiff]

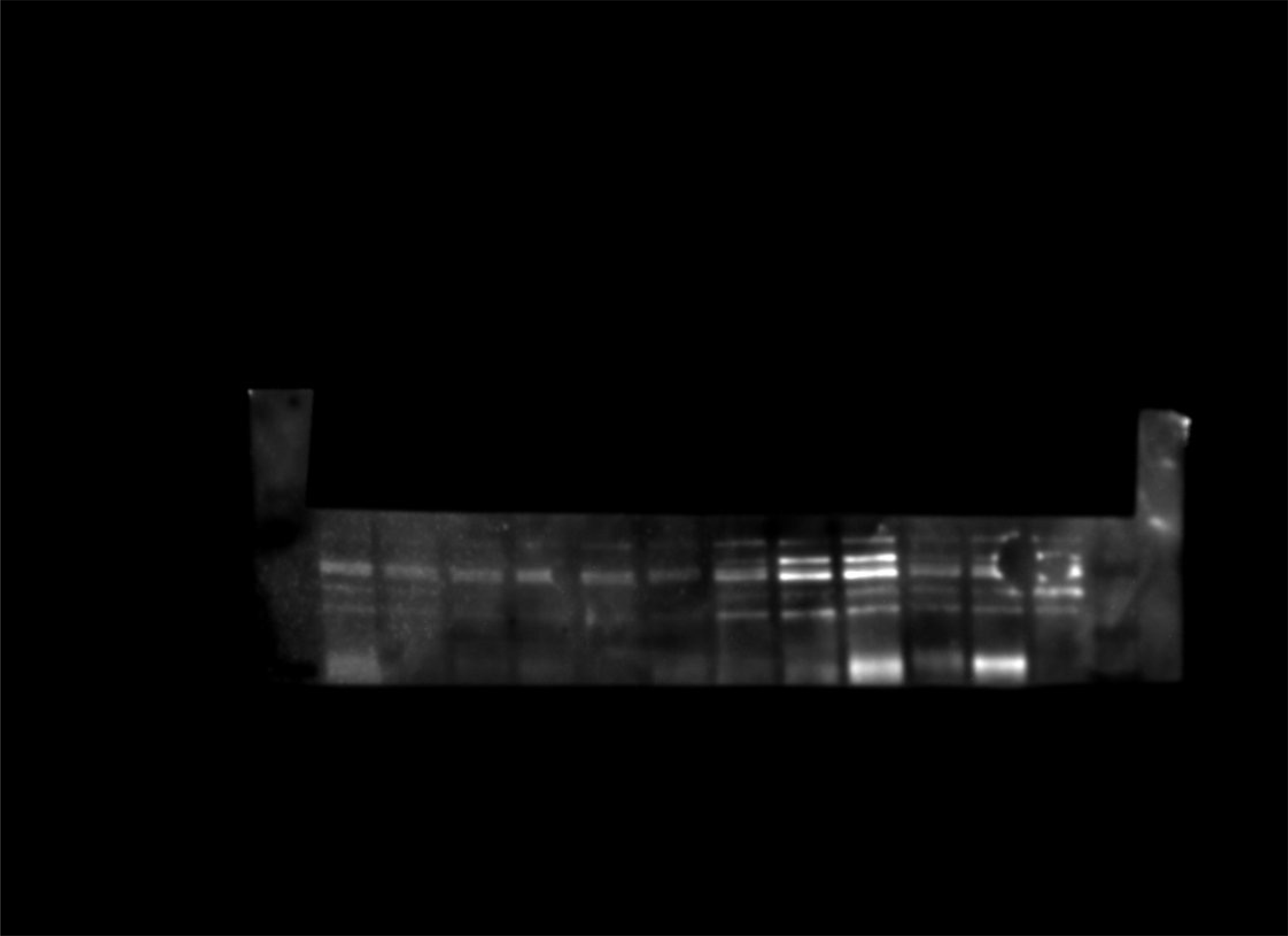

Supplement: Figure 7—figure supplement 1—source data 1. — Labelled (.pdf) and raw (folder) blot images showed in panel F are also included. [file elife-79840-fig7-figsupp1-data1.zip › Figure 7 - supplement figure 1 - data source/Blot Figure 7 - figure supplement S1/Figure 7-S1F/Lane-COL1-panel-7S1F.tiff]

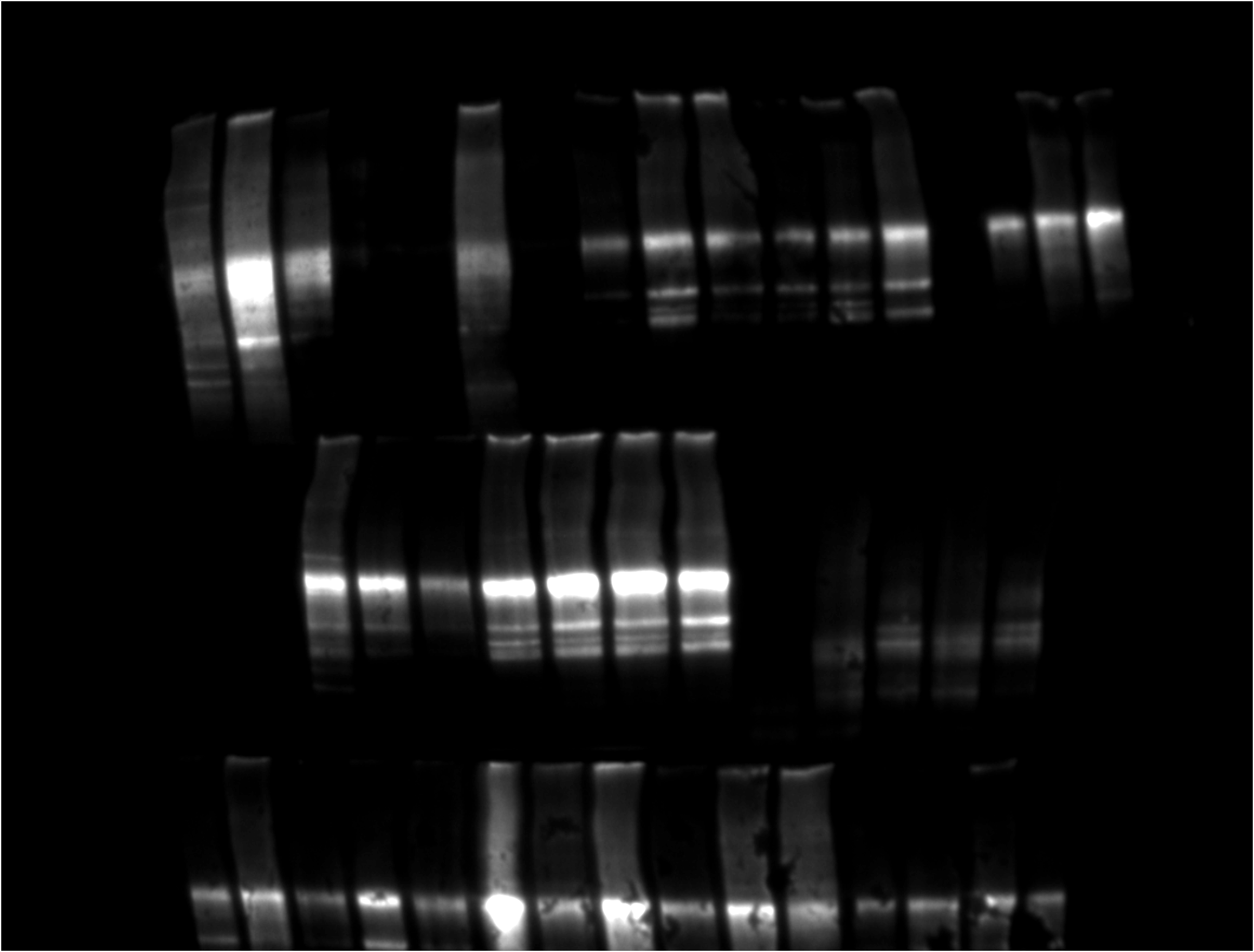

Supplement: Figure 8—source data 1. — Labelled (.pdf) and raw (folder) blot images showed in panel B are also included. [file elife-79840-fig8-data1.zip › Figure 8 - source data/ Blot Figure 8B/Lane_FN1-B.tiff]

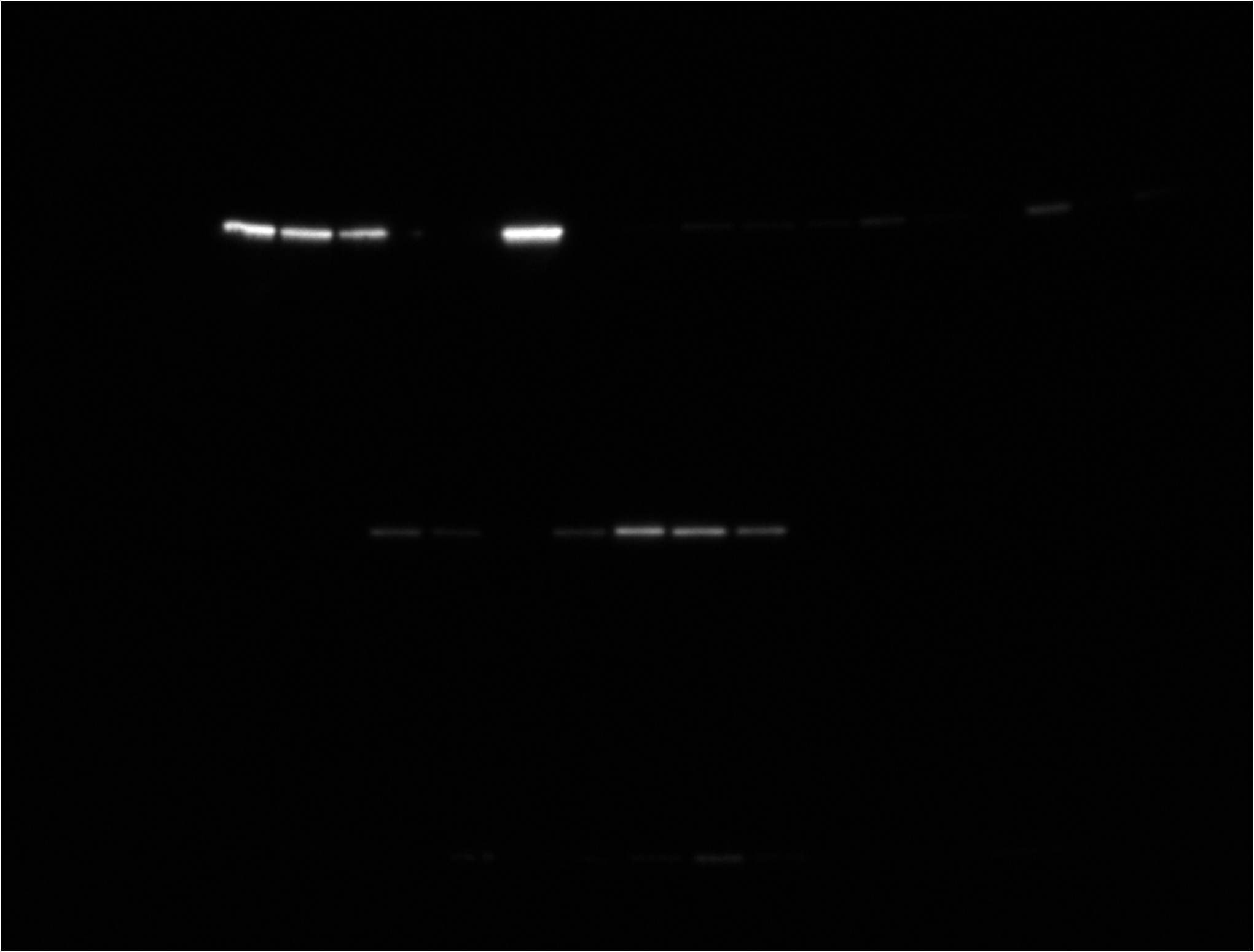

Supplement: Figure 8—source data 1. — Labelled (.pdf) and raw (folder) blot images showed in panel B are also included. [file elife-79840-fig8-data1.zip › Figure 8 - source data/ Blot Figure 8B/Lane_ACTA2-B.tiff]

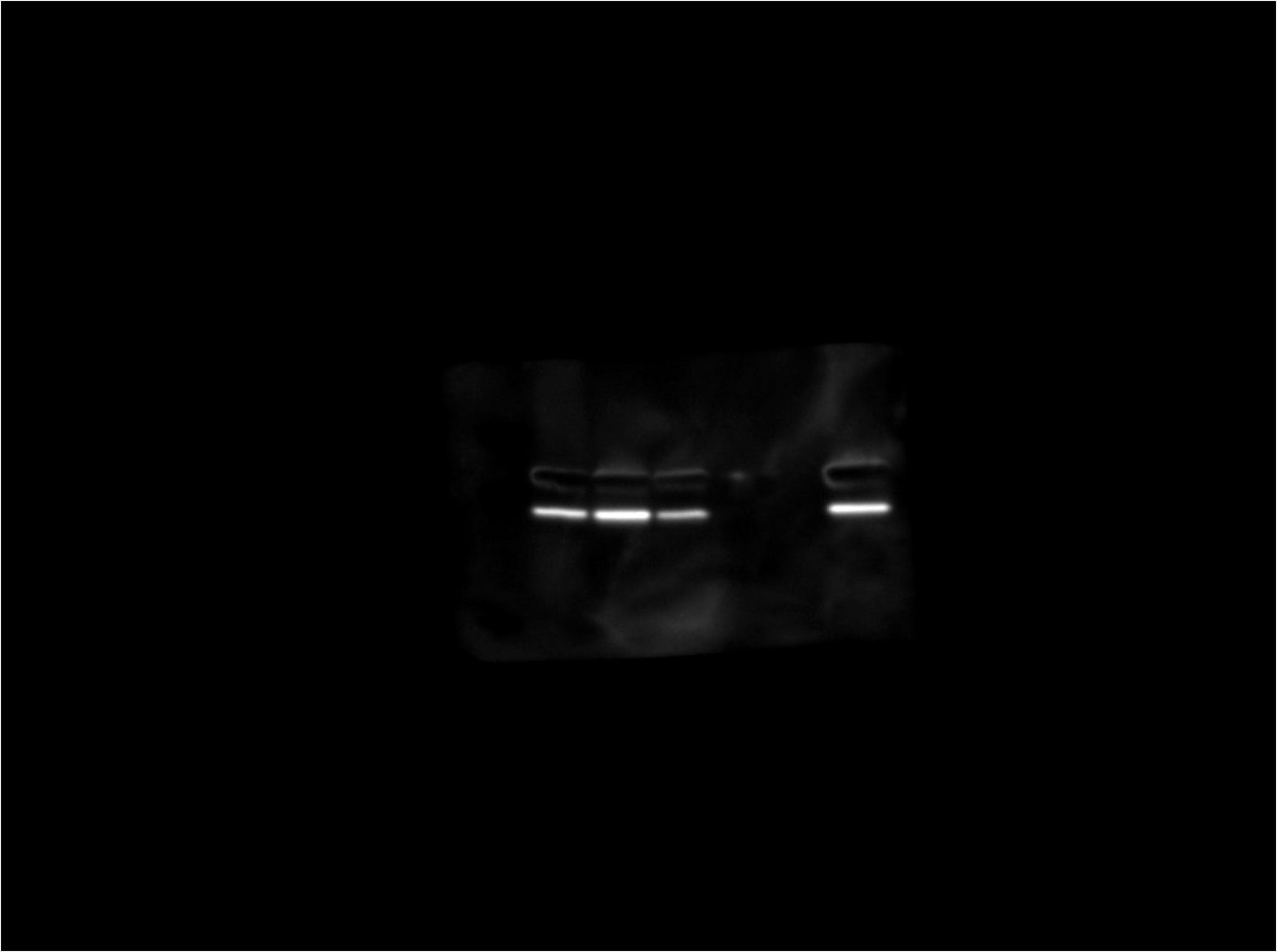

Supplement: Figure 8—source data 1. — Labelled (.pdf) and raw (folder) blot images showed in panel B are also included. [file elife-79840-fig8-data1.zip › Figure 8 - source data/ Blot Figure 8B/Lane_TUB-B.tiff]

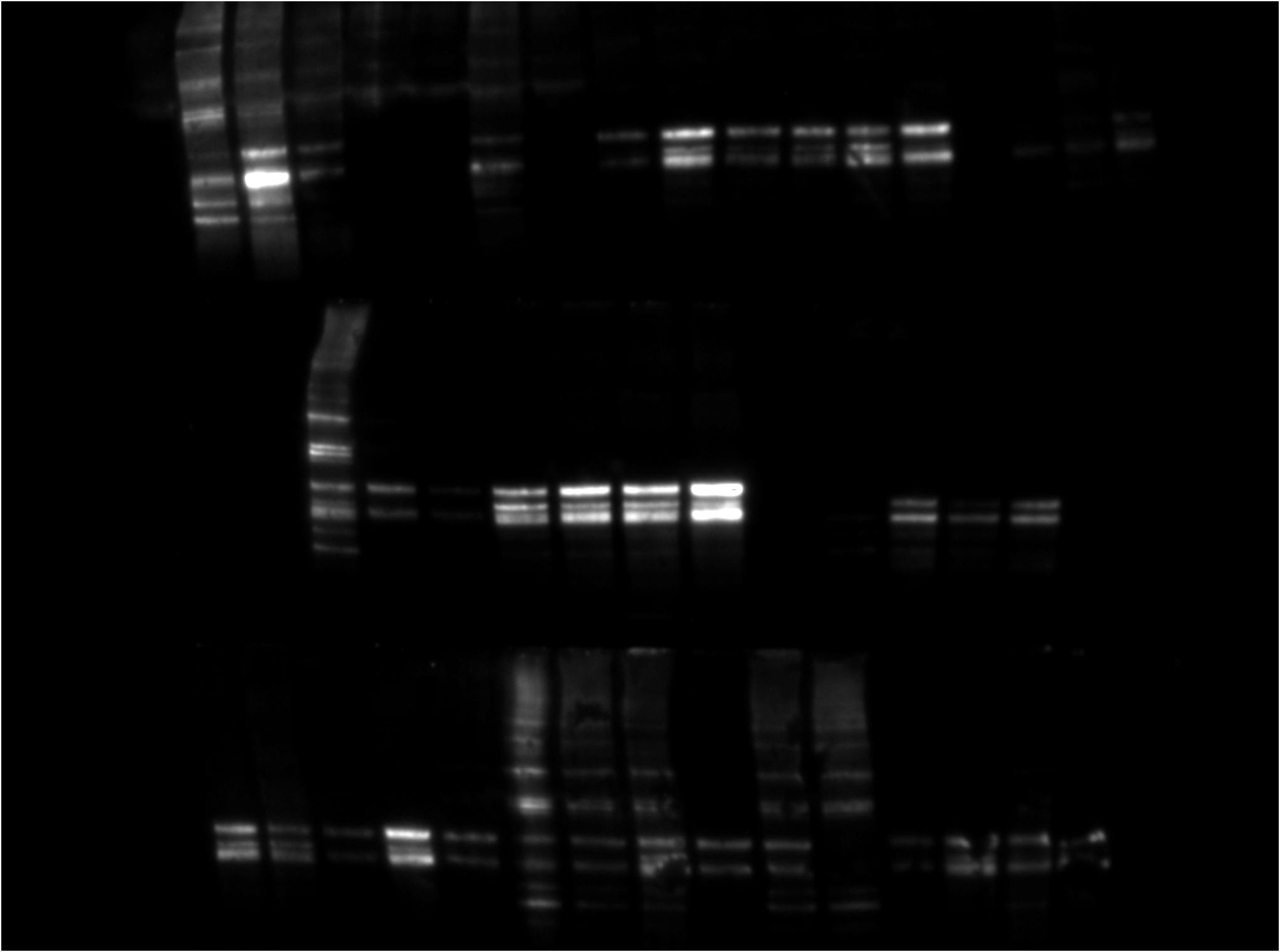

Supplement: Figure 8—source data 1. — Labelled (.pdf) and raw (folder) blot images showed in panel B are also included. [file elife-79840-fig8-data1.zip › Figure 8 - source data/ Blot Figure 8B/Lane_COL1-B.tiff]

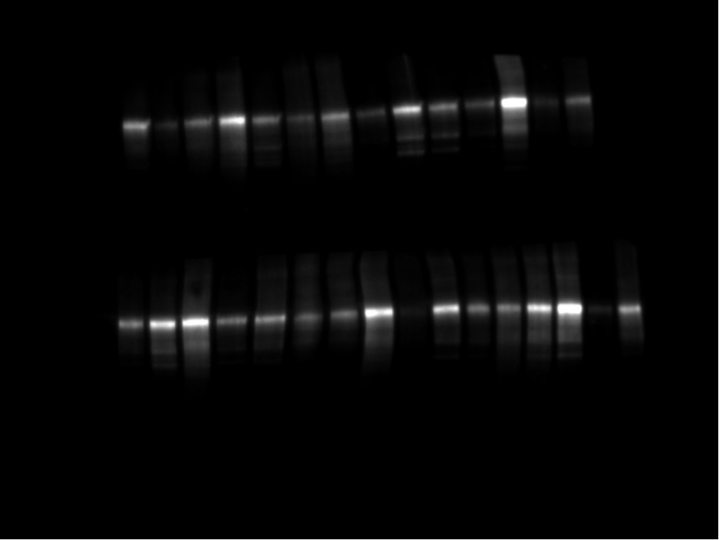

Supplement: Figure 8—figure supplement 1—source data 1. — Labelled (.pdf) and raw (folder) blot images showed in panel B are also included. [file elife-79840-fig8-figsupp1-data1.zip › Figure 8 - supplement figure 1 - source data/Figure 8 - figure supplement S1/Figure 8-S1B/Lane-FN1-8S1B.tiff]

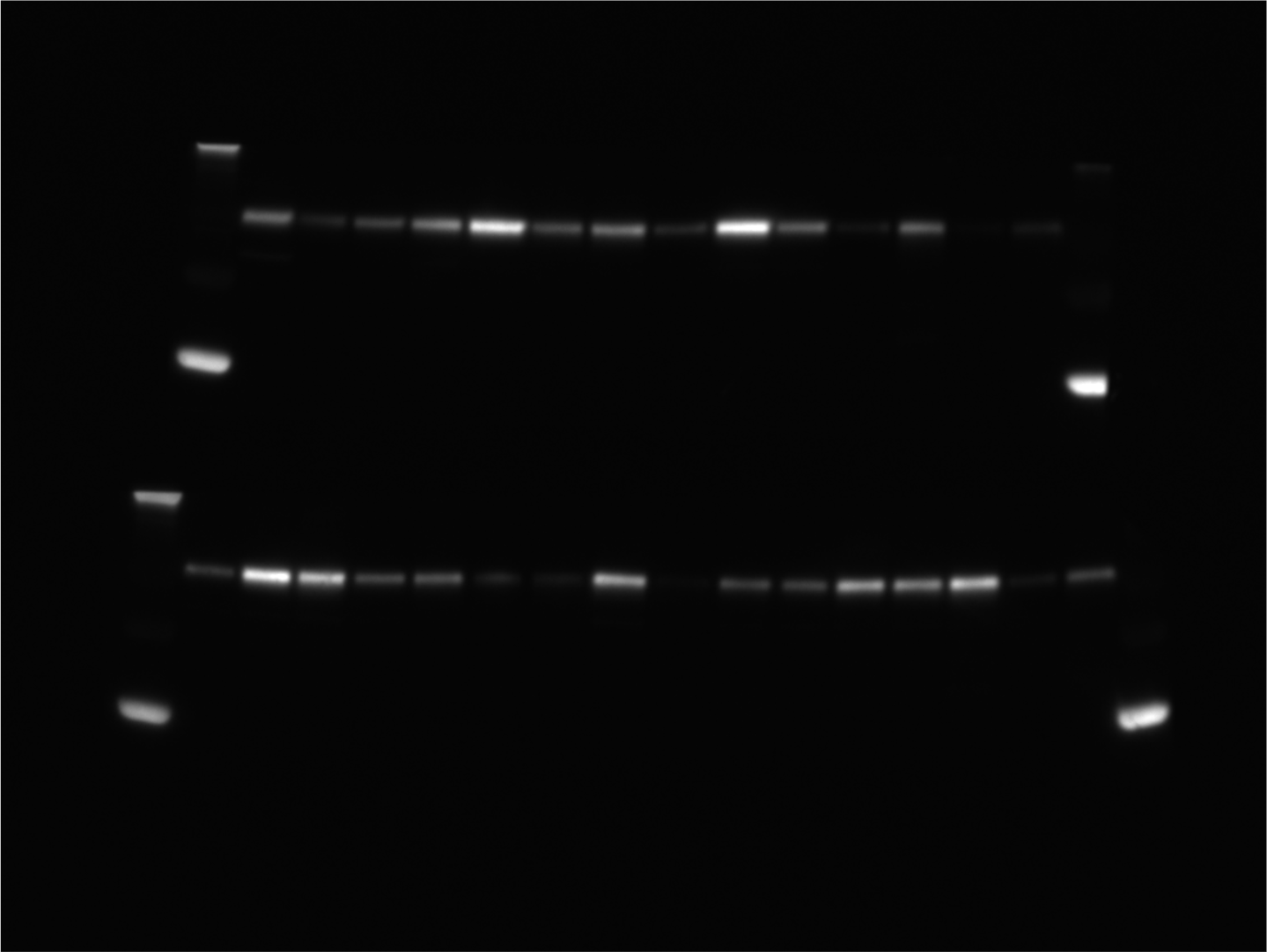

Supplement: Figure 8—figure supplement 1—source data 1. — Labelled (.pdf) and raw (folder) blot images showed in panel B are also included. [file elife-79840-fig8-figsupp1-data1.zip › Figure 8 - supplement figure 1 - source data/Figure 8 - figure supplement S1/Figure 8-S1B/Lane_TUB-8S1B.tiff]

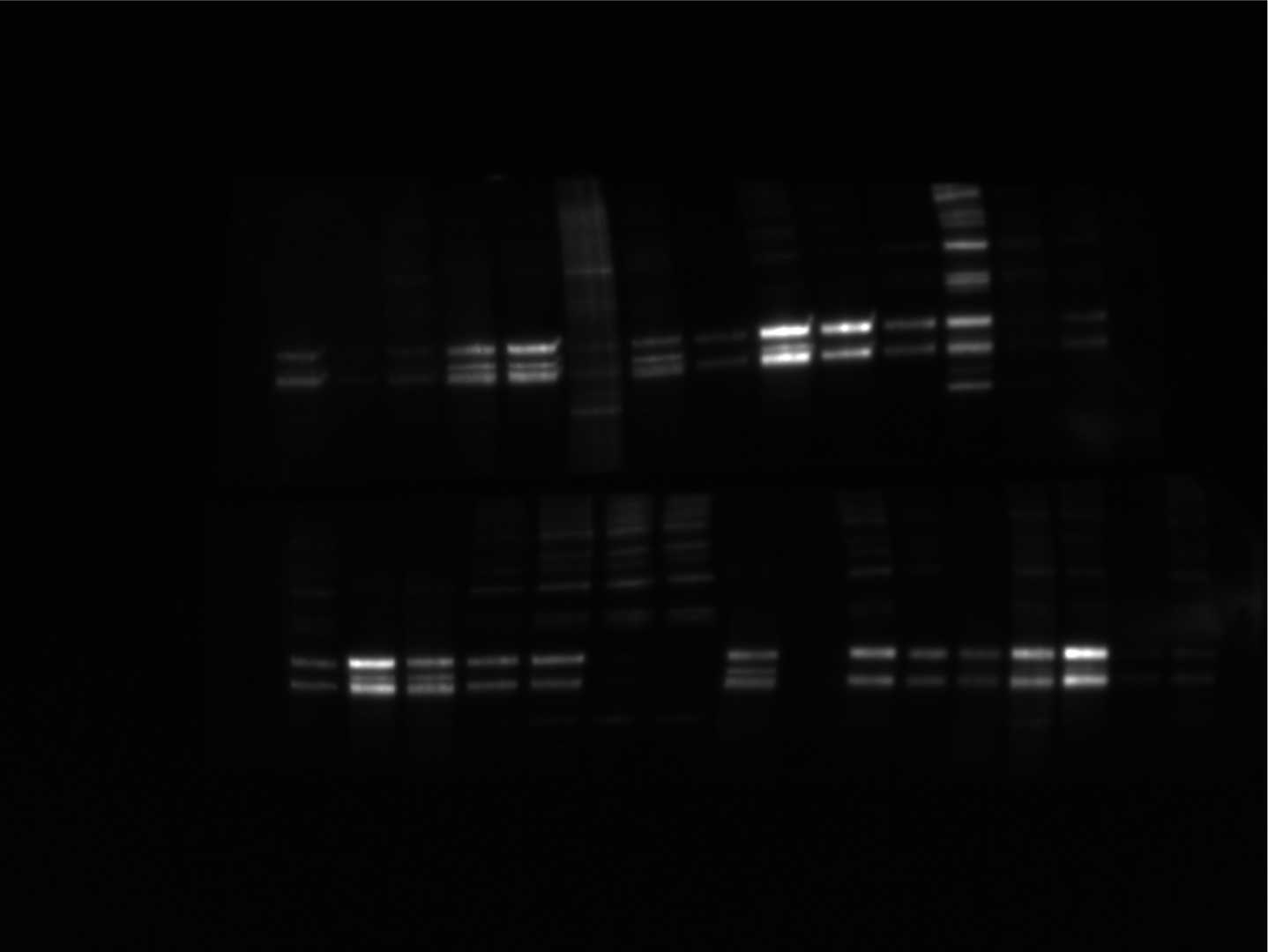

Supplement: Figure 8—figure supplement 1—source data 1. — Labelled (.pdf) and raw (folder) blot images showed in panel B are also included. [file elife-79840-fig8-figsupp1-data1.zip › Figure 8 - supplement figure 1 - source data/Figure 8 - figure supplement S1/Figure 8-S1B/Lane-COL1-8S1B.tiff]

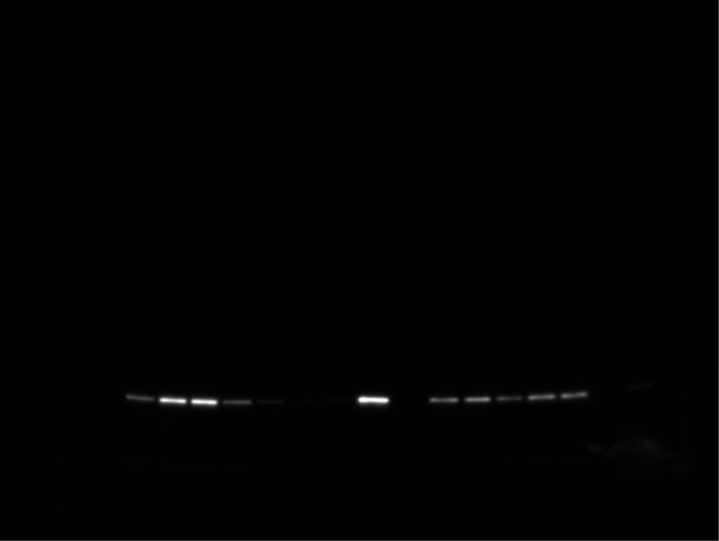

Supplement: Figure 8—figure supplement 1—source data 1. — Labelled (.pdf) and raw (folder) blot images showed in panel B are also included. [file elife-79840-fig8-figsupp1-data1.zip › Figure 8 - supplement figure 1 - source data/Figure 8 - figure supplement S1/Figure 8-S1B/Lane-ACTA2-8S1B.tiff]
