# Supplementary material for: Identification of Paired-related Homeobox Protein 1 as a key mesenchymal transcription factor in pulmonary fibrosis
Source: eLife. 2023 Jun 1;12:e79840. doi: 10.7554/eLife.79840 (PMC10275639; doi:10.7554/eLife.79840)

Blots for Figure 3-figure supplement 1A

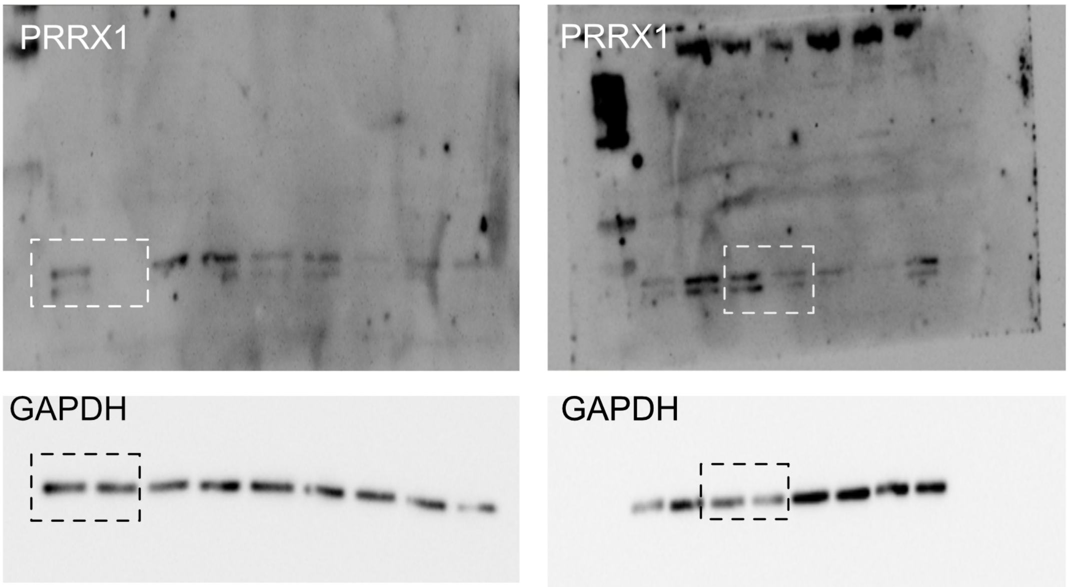

Blots for Figure 3-figure supplement 1D

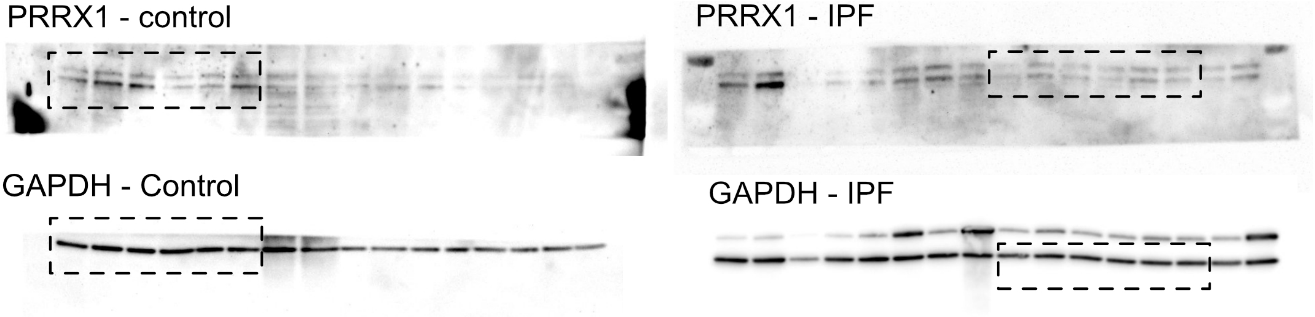

Blots for Figure 3-figure supplement 1E

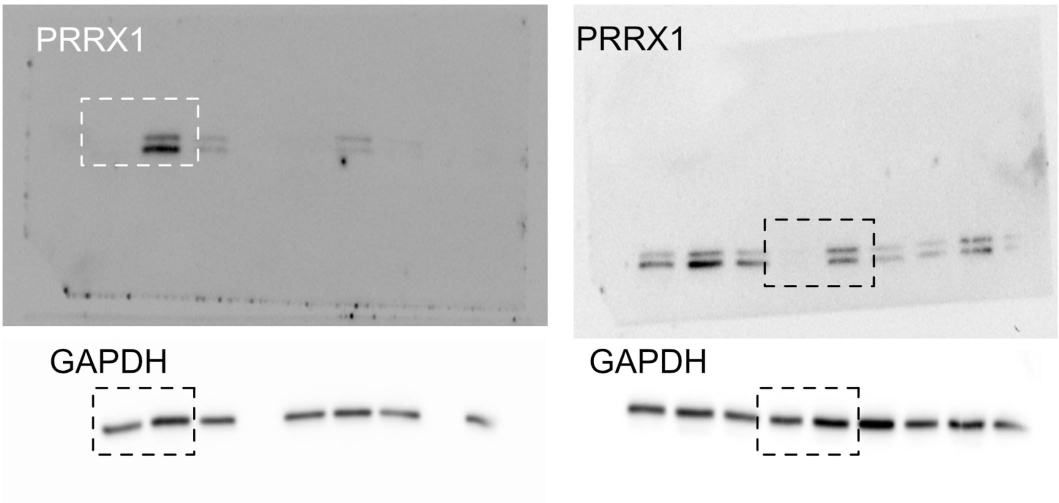

Blots for Figure 5-figure supplement 1B

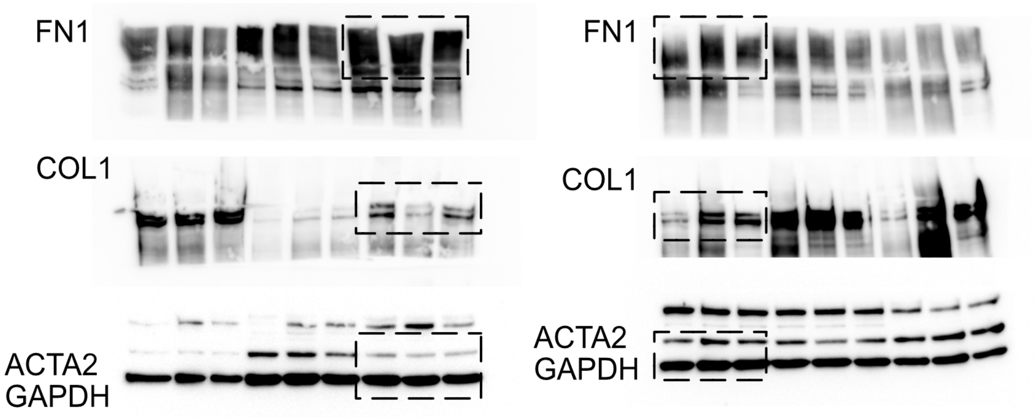

Blots for Figure 5-figure supplement 2C

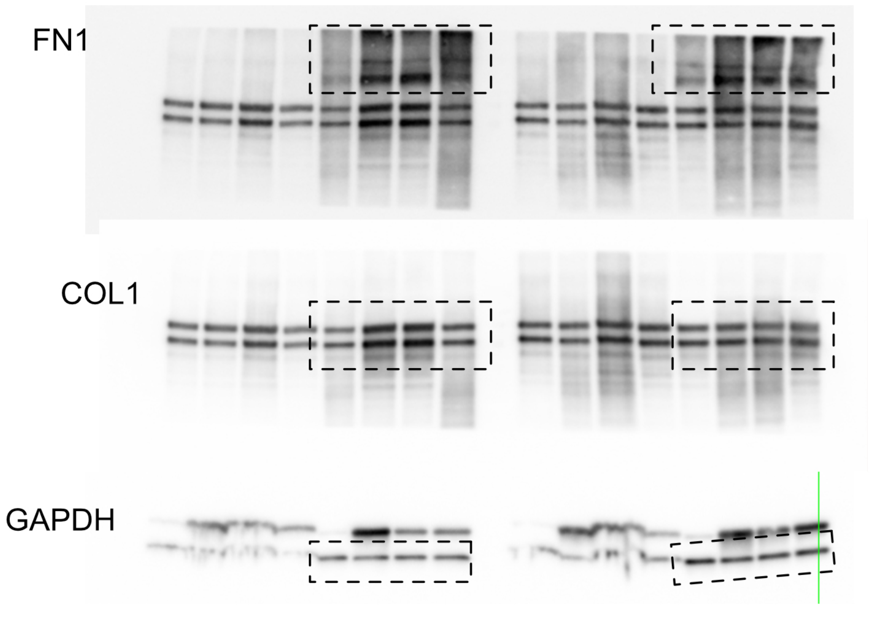

Blots for Figure 5-figure supplement 4A

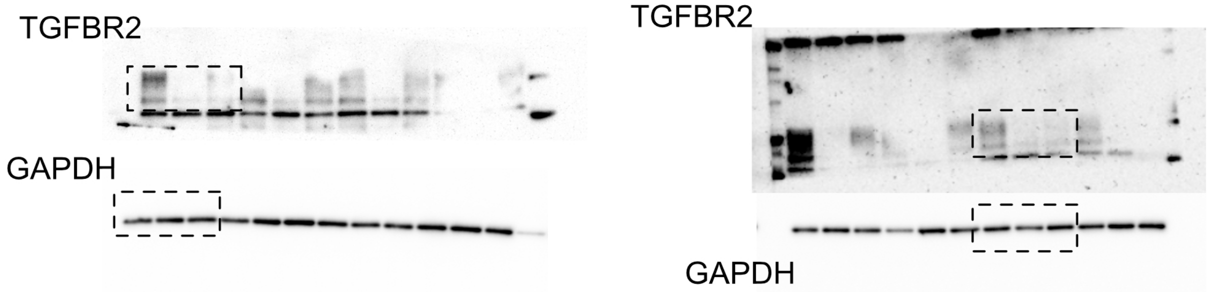

Blots for Figure 5-figure supplement 4B

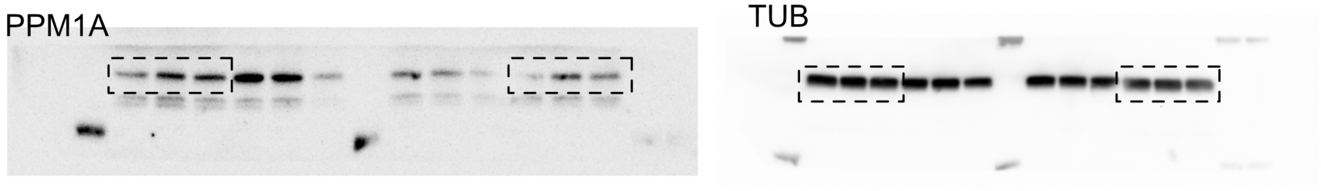

Blots for Figure 5-figure supplement 4C

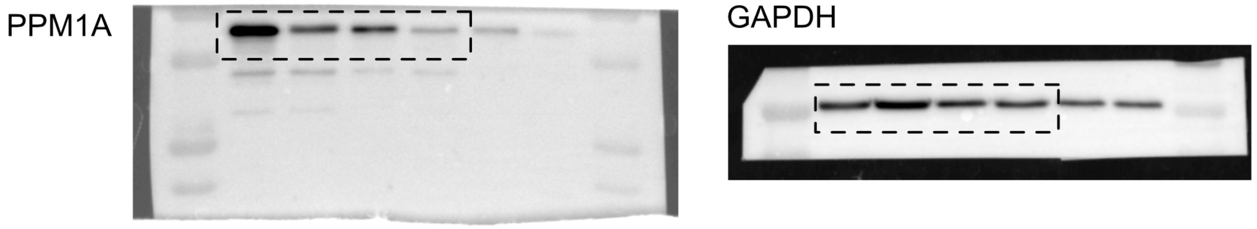

Blots for igure 5-figure supplement 4D

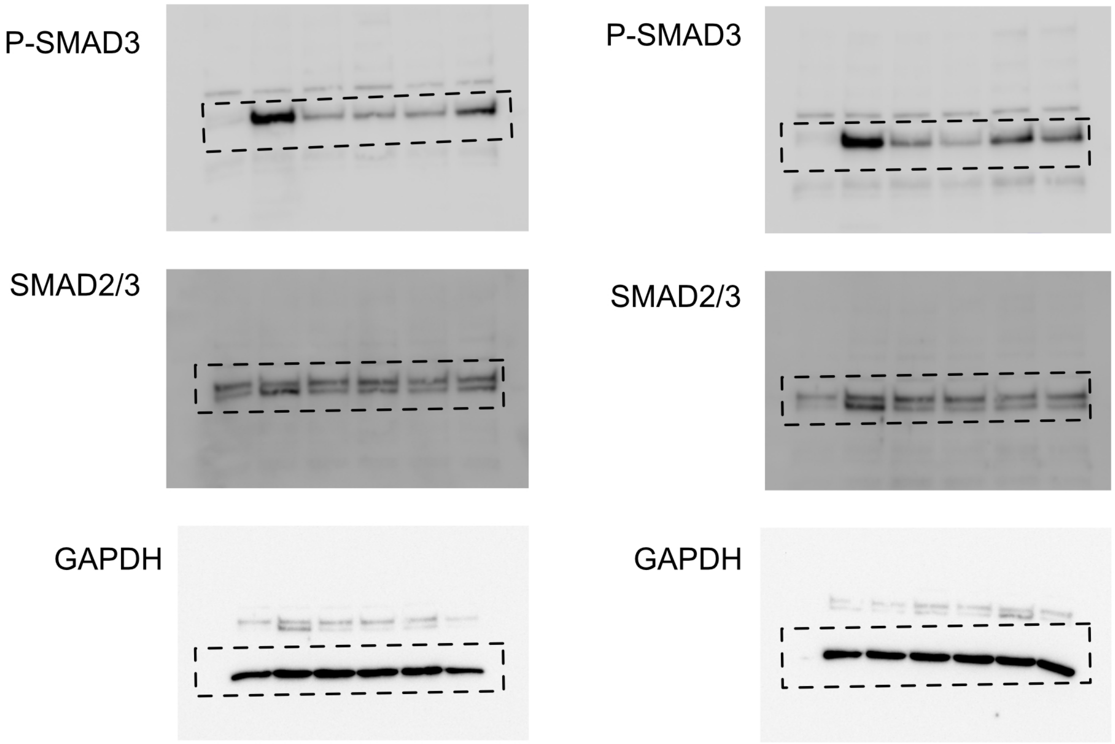

Blots for igure 5-figure supplement 5A

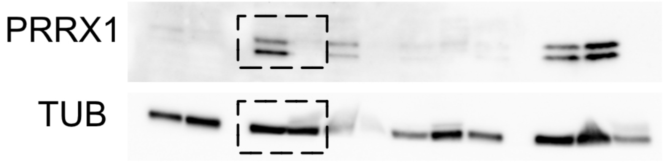

Blots for Figure 5-figure supplement 5C

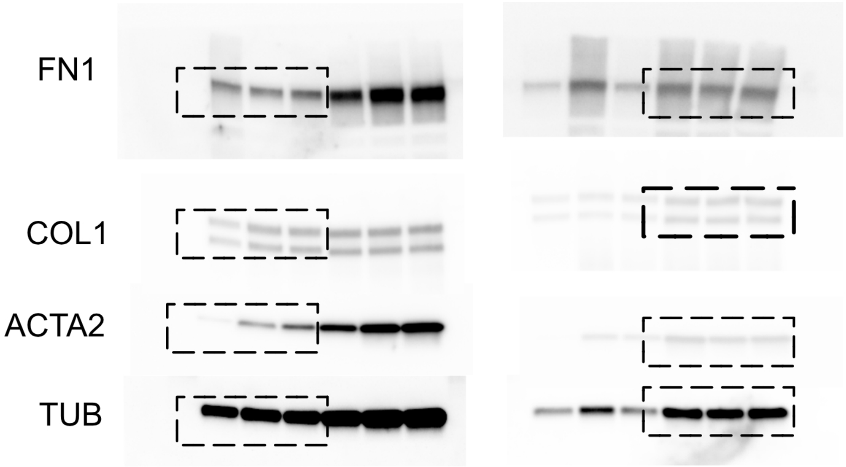

Blots for Figure 6-figure supplement 1B

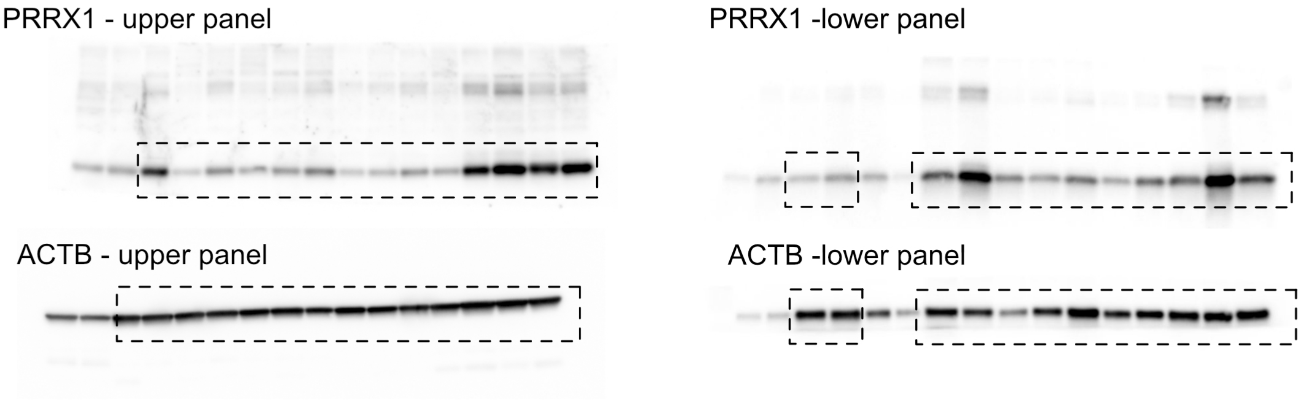

Blots for Figure 6-figure supplement 2B

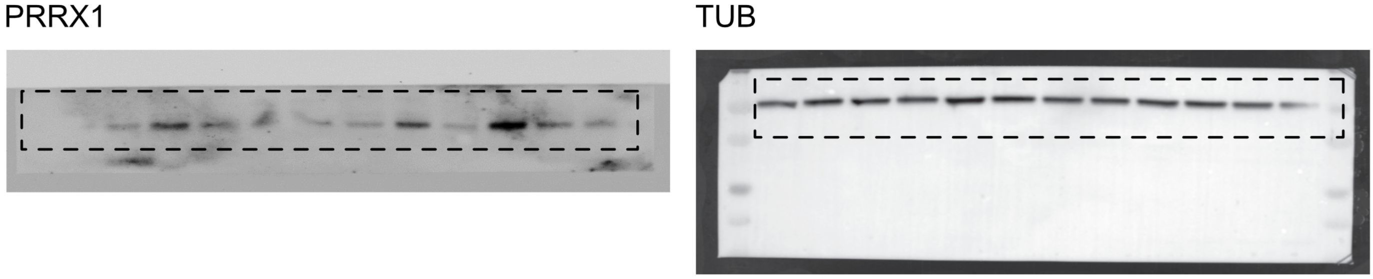

Blots for Figure 6-figure supplement 2G

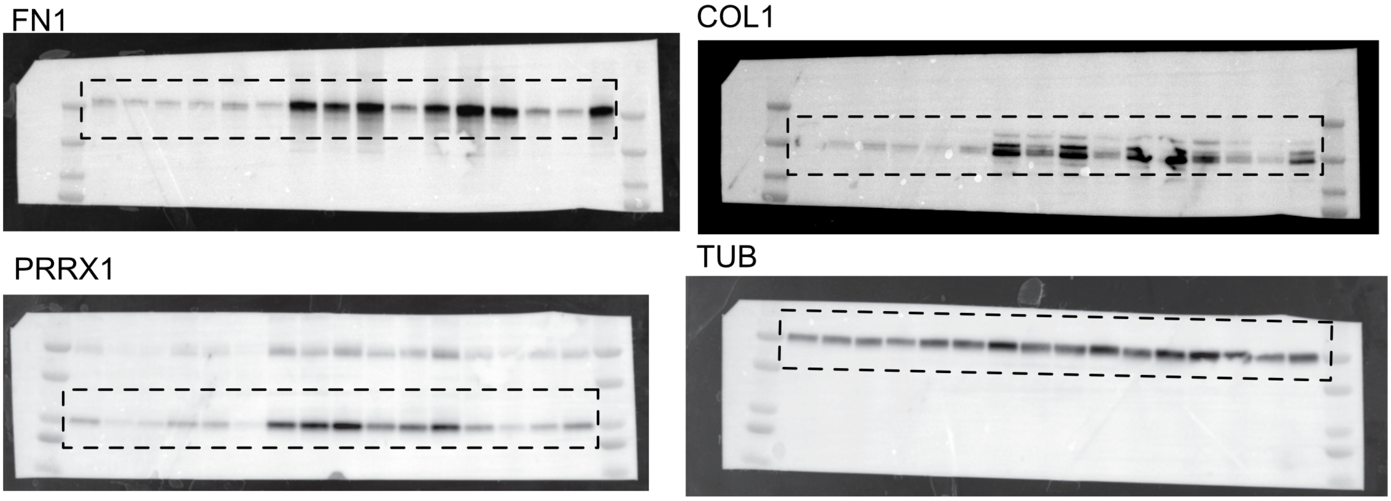

Blots for Figure 7-figure supplement 1F

FN1

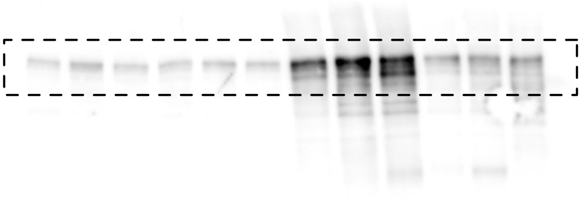

COL1

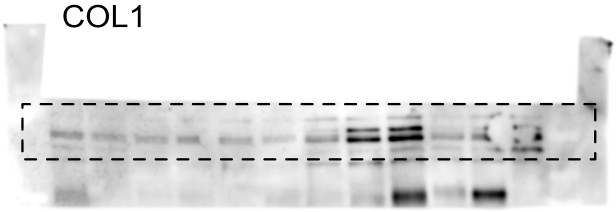

PRRX1

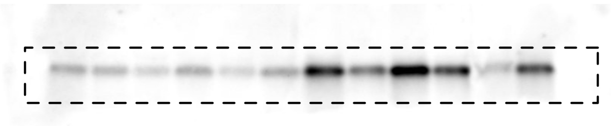

TUB

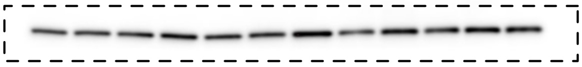

Blots for Figure 8-figure supplement 1B

FN1

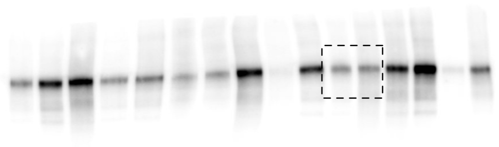

COL1

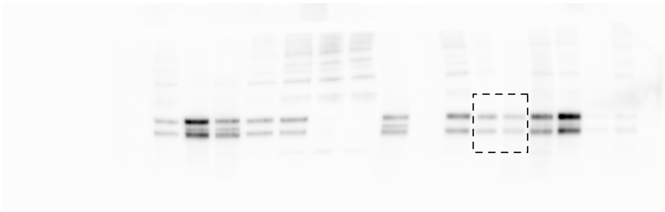

ACTA2

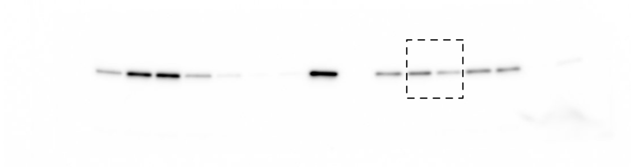

TUB

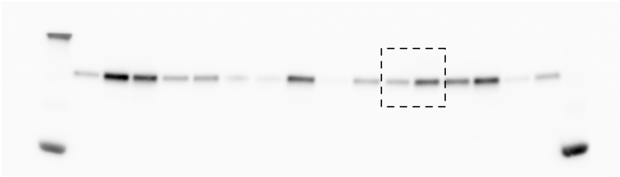

Supplement: Figure 3—figure supplement 1—source data 1. — Labelled (.pdf) and raw (folder) blot images showed in panels A,D, and E are also included. [file elife-79840-fig3-figsupp1-data1.zip › Figure 3-figure supplement 1 - source data/Blot-SupFigures-labelled.pdf]
