# Supplementary material for: Identification of Paired-related Homeobox Protein 1 as a key mesenchymal transcription factor in pulmonary fibrosis
Source: eLife. 2023 Jun 1;12:e79840. doi: 10.7554/eLife.79840 (PMC10275639; doi:10.7554/eLife.79840)
Supplement: Supplementary file 1. [file elife-79840-supp1.docx]

**Supplementary Table S1: list of common up-regulated genes in all three IPF transcriptome database analyzed.**

| **UPREGULATED GENES ANNOTATED AS TRANSCRIPTION FACTORS (PANTHER GO)** | | | |
| --- | --- | --- | --- |
| Gene symbol | Name | Gene symbol | Name |
| FHL2 | four and a half LIM domains 2 | SMAD1 | SMAD family member 1 |
| NFATC4 | nuclear factor of activated T-cells 4 | SOX2 | SRY-box 2 |
| NR2F2 | nuclear receptor subfamily 2 group F member 2 | TP63 | tumor protein p63 |
| **PRRX1** | **paired related homeobox 1** | TRIM29 | tripartite motif containing 29 |
| RHBDD3 | rhomboid domain containing 3 | ZNF671 | zinc finger protein 671 |
| RUNX1 | runt related transcription factor 1 | ZNF862 | zinc finger protein 862 |
| **OTHER UPREGULATED GENES (alphabetical order in column)** | | | |
| Gene symbol | Name | Gene symbol | Name |
| AAK1 | AP2 associated kinase 1 | LPP | LIM domain containing preferred translocation partner in lipoma |
| ABCC5 | ATP binding cassette subfamily C member 5 | LRRC17 | leucine rich repeat containing 17 |
| ACTN1 | actinin alpha 1 | LTBP1 | latent transforming growth factor beta binding protein 1 |
| ADCY3 | adenylate cyclase 3 | MAGED4B | MAGE family member D4B |
| ALDH3A1 | aldehyde dehydrogenase 3 family member A1 | MAP1A | microtubule associated protein 1A |
| ANGPTL2 | angiopoietin like 2 | MAP4K4 | mitogen-activated protein kinase kinase kinase kinase 4 |
| ANKH | ANKH inorganic pyrophosphate transport regulator | MFAP2 | microfibrillar associated protein 2 |
| ANTXR1 | anthrax toxin receptor 1 | MIR4697HG | MIR4697 host gene |
| AP2A1 | adaptor related protein complex 2 alpha 1 subunit | MMP7 | matrix metallopeptidase 7 |
| ASB2 | ankyrin repeat and SOCS box containing 2 | MOXD1 | monooxygenase DBH like 1 |
| ASPN | asporin | MRVI1 | murine retrovirus integration site 1 homolog |
| BACE2 | beta-site APP-cleaving enzyme 2 | MXRA5 | matrix remodeling associated 5 |
| BAG4 | BCL2 associated athanogene 4 | MYOF | Myoferlin |
| BCAS4 | breast carcinoma amplified sequence 4 | NAV2 | neuron navigator 2 |
| BPIFB1 | BPI fold containing family B member 1 | NLGN2 | neuroligin 2 |
| BRD8 | bromodomain containing 8 | NSG1 | neuron specific gene family member 1 |
| C8orf44 | chromosome 8 open reading frame 44 | OGN | Osteoglycin |
| CADPS | calcium dependent secretion activator | OLFM1 | olfactomedin 1 |
| CAMK1D | calcium/calmodulin dependent protein kinase ID | ORAI2 | ORAI calcium release-activated calcium modulator 2 |
| CAPN5 | calpain 5 | OSBPL6 | oxysterol binding protein like 6 |
| CARM1 | coactivator associated arginine methyltransferase 1 | P2RX5 | purinergic receptor P2X 5 |
| CC2D2A | coiled-coil and C2 domain containing 2A | P3H3 | prolyl 3-hydroxylase 3 |
| CCDC142 | coiled-coil domain containing 142 | P3H4 | prolyl 3-hydroxylase family member 4 |
| CCDC88C | coiled-coil domain containing 88C | PCDH7 | protocadherin 7 |
| CCL13 | C-C motif chemokine ligand 13 | PCNX2 | pecanex homolog 2 |
| CCL18 | C-C motif chemokine ligand 18 | PDLIM4 | PDZ and LIM domain 4 |
| CCND2 | cyclin D2 | PDLIM7 | PDZ and LIM domain 7 |
| CD248 | CD248 molecule | PGM5 | phosphoglucomutase 5 |
| CDH3 | cadherin 3 | PIK3R2 | phosphoinositide-3-kinase regulatory subunit 2 |
| CDKN2C | cyclin dependent kinase inhibitor 2C | PKIB | protein kinase (cAMP-dependent, catalytic) inhibitor beta |
| CEP250 | centrosomal protein 250 | PLA2G15 | phospholipase A2 group XV |
| CFH | complement factor H | PLEKHA4 | pleckstrin homology domain containing A4 |
| CHST3 | carbohydrate sulfotransferase 3 | POSTN | Periostin |
| CLDN15 | claudin 15 | PPP1R12B | protein phosphatase 1 regulatory subunit 12B |
| CLIP2 | CAP-Gly domain containing linker protein 2 | PRAF2 | PRA1 domain family member 2 |
| CLMN | calmin | PRUNE2 | prune homolog 2 |
| COL14A1 | collagen type XIV alpha 1 chain | PSD3 | pleckstrin and Sec7 domain containing 3 |
| COL15A1 | collagen type XV alpha 1 chain | PTGFRN | prostaglandin F2 receptor inhibitor |
| COL16A1 | collagen type XVI alpha 1 chain | R3HDM1 | R3H domain containing 1 |
| COL18A1 | collagen type XVIII alpha 1 chain | RAB3GAP1 | RAB3 GTPase activating protein catalytic subunit 1 |
| COL5A2 | collagen type V alpha 2 chain | RAMP1 | receptor activity modifying protein 1 |
| COL6A1 | collagen type VI alpha 1 chain | RBBP4 | RB binding protein 4, chromatin remodeling factor |
| COL6A2 | collagen type VI alpha 2 chain | RHOD | ras homolog family member D |
| COL6A3 | collagen type VI alpha 3 chain | RNFT2 | ring finger protein, transmembrane 2 |
| COL7A1 | collagen type VII alpha 1 chain | ROR2 | receptor tyrosine kinase like orphan receptor 2 |
| COMP | cartilage oligomeric matrix protein | S100A2 | S100 calcium binding protein A2 |
| CP | ceruloplasmin | SCARA3 | scavenger receptor class A member 3 |
| CPXM1 | carboxypeptidase X, M14 family member 1 | SCG5 | secretogranin V |
| CXCL14 | C-X-C motif chemokine ligand 14 | SEMA3C | semaphorin 3C |
| DCLK1 | doublecortin like kinase 1 | SERINC2 | serine incorporator 2 |
| DDO | D-aspartate oxidase | SERPINB5 | serpin family B member 5 |
| DIO2 | deiodinase, iodothyronine type II | SEZ6L2 | seizure related 6 homolog like 2 |
| DOCK3 | dedicator of cytokinesis 3 | SFI1 | SFI1 centrin binding protein |
| DOK5 | docking protein 5 | SFXN4 | sideroflexin 4 |
| ECM1 | extracellular matrix protein 1 | SH3GL1 | SH3 domain containing GRB2 like 1, endophilin A2 |
| EFNB3 | ephrin B3 | SHC3 | SHC adaptor protein 3 |
| EGFL6 | EGF like domain multiple 6 | SLC1A4 | solute carrier family 1 member 4 |
| EML6 | echinoderm microtubule associated protein like 6 | SLC22A23 | solute carrier family 22 member 23 |
| EPHB2 | EPH receptor B2 | SLC29A3 | solute carrier family 29 member 3 |
| EYA2 | EYA transcriptional coactivator and phosphatase 2 | SLC4A4 | solute carrier family 4 member 4 |
| FBLN1 | fibulin 1 | SLC6A8 | solute carrier family 6 member 8 |
| FBLN2 | fibulin 2 | SNCAIP | synuclein alpha interacting protein |
| FBXW9 | F-box and WD repeat domain containing 9 | SPP1 | secreted phosphoprotein 1 |
| FHOD3 | formin homology 2 domain containing 3 | SRGAP3 | SLIT-ROBO Rho GTPase activating protein 3 |
| FLRT2 | fibronectin leucine rich transmembrane protein 2 | STK36 | serine/threonine kinase 36 |
| FMO1 | flavin containing monooxygenase 1 | STK38 | serine/threonine kinase 38 |
| FUT6 | fucosyltransferase 6 | STMN3 | stathmin 3 |
| GABBR2 | gamma-aminobutyric acid type B receptor subunit 2 | SYNDIG1 | synapse differentiation inducing 1 |
| GALNT5 | polypeptide N-acetylgalactosaminyltransferase 5 | SYNJ2 | synaptojanin 2 |
| GDF11 | growth differentiation factor 11 | SYNPO2 | synaptopodin 2 |
| GLT8D2 | glycosyltransferase 8 domain containing 2 | SYNRG | synergin gamma |
| GPC1 | glypican 1 | SYT8 | synaptotagmin 8 |
| GPR87 | G protein-coupled receptor 87 | SYTL2 | synaptotagmin like 2 |
| GPX7 | glutathione peroxidase 7 | TAB1 | TGF-beta activated kinase 1 binding protein 1 |
| GUCY1A3 | guanylate cyclase 1 soluble subunit alpha | TENM4 | teneurin transmembrane protein 4 |
| H2AFY2 | H2A histone family member Y2 | TFF3 | trefoil factor 3 |
| HEPH | hephaestin | TGFB3 | transforming growth factor beta 3 |
| HOMER3 | homer scaffolding protein 3 | TGFBI | transforming growth factor beta induced |
|  |  | TGFBR2 | transforming growth factor beta receptor 2 |
| HS3ST1 | heparan sulfate-glucosamine 3-sulfotransferase 1 | THBS2 | thrombospondin 2 |
| HSD3B7 | hydroxy-delta-5-steroid dehydrogenase, 3 beta- and steroid delta-isomerase 7 | TLDC1 | TBC/LysM-associated domain containing 1 |
| HSPA4L | heat shock protein family A member 4 like | TM7SF3 | transmembrane 7 superfamily member 3 |
| IGF1 | insulin like growth factor 1 | TMED10 | transmembrane p24 trafficking protein 10 |
| IGFBP2 | insulin like growth factor binding protein 2 | TMEM158 | transmembrane protein 158 (gene/pseudogene) |
| IL13RA2 | interleukin 13 receptor subunit alpha 2 | TMEM40 | transmembrane protein 40 |
| ILF3 | interleukin enhancer binding factor 3 | TNPO1 | transportin 1 |
| INPP5D | inositol polyphosphate-5-phosphatase D | TP73 | tumor protein p73 |
| ITGA7 | integrin subunit alpha 7 | TRA2A | transformer 2 alpha homolog |
| ITGB4 | integrin subunit beta 4 | TRO | trophinin |
| KCNMA1 | potassium calcium-activated channel subfamily M alpha 1 | TRPM4 | transient receptor potential cation channel subfamily M member 4 |
| KCNMB1 | potassium calcium-activated channel subfamily M regulatory beta subunit 1 | UGT1A9 | UDP glucuronosyltransferase family 1 member A9 |
| KCNN3 | potassium calcium-activated channel subfamily N member 3 | USP35 | ubiquitin specific peptidase 35 |
| KCNN4 | potassium calcium-activated channel subfamily N member 4 | VILL | villin like |
| KIAA0100 | KIAA0100 | VWA1 | von Willebrand factor A domain containing 1 |
| KIAA0101 | KIAA0101 (PCLAF) | WRAP53 | WD repeat containing antisense to TP53 |
| LDOC1 | leucine zipper down-regulated in cancer 1 | XPR1 | xenotropic and polytropic retrovirus receptor 1 |
| LGALS7 | galectin 7 | ZDHHC13 | zinc finger DHHC-type containing 13 |
| LOC730101 | uncharacterized LOC730101 | ZMIZ1 | zinc finger MIZ-type containing 1 |
| LOXL1 | lysyl oxidase like 1 | ZNF207 | zinc finger protein 207 |
| LOXL2 | lysyl oxidase like 2 | ZNF71 | zinc finger protein 71 |
