# Supplementary material for: Identification of Paired-related Homeobox Protein 1 as a key mesenchymal transcription factor in pulmonary fibrosis
Source: eLife. 2023 Jun 1;12:e79840. doi: 10.7554/eLife.79840 (PMC10275639; doi:10.7554/eLife.79840)
Supplement: Supplementary file 2. — The table contains the best 597 genes significantly modulated by TGF-β in NHLF treated with siControl#1 and #2 (log2 fold change >4; adj. P Val <0.05). The table indicates the Fold Change (FC) for each of the genes: (i) column 1: TGF-β1" stimulation in presence of siControl#1; column 2: TGFβ1 fold stimulation in presence of siRNA#1; column 4: TGFβ1 fold stimulation in presence of siControl#2; column 5: TGFβ1 fold stimulation in presence of siRNA#2". Columns 5 and 6 give the percentage of residual modulation by TGF-β for each PRRX1 siRNA. Modulations are shown in progressively brighter shades of blue (attenuation) and orange (over-activation). The mean residual fold change following PRRX1 KD is 56.6% (siRNA#1) and 80.0% (siRNA#2). [file elife-79840-supp2.docx]

**Supplementary Table S2: Attenuation of the TGF-β response following PRRX1 knock-down.**

The table contains the best 597 genes significantly modulated by TGF-β in NHLF treated with siControl#1 and #2 (log2 fold change >4 ; adj. P Val<0.05). The table indicates the Fold Change (FC) for each of the genes: i) column 1: TGF-β1" stimulation in presence of siControl#1 ; column 2: TGFβ1 fold stimulation in presence of siRNA#1 ; column 4: TGFβ1 fold stimulation in presence of siControl#2 ; column 5: TGFβ1 fold stimulation in presence of siRNA#2". Columns 5 and 6 give the percentage of residual modulation by TGF-β for each PRRX1 siRNA. Modulations are shown in progressively brighter shades of blue (attenuation) and orange (over-activation). The mean residual fold change following PRRX1 KD is 56.6 % (siRNA#1) and 80.0 % (siRNA#2).

| **GeneName** | **TGF-β1 fold modulation** | | **Residual FC (%) siRNA#1** | **TGF-β1 fold modulation** | | **Residual FC (%) siRNA#2** |
| --- | --- | --- | --- | --- | --- | --- |
|  | **siControl#1** | **PRRX1 siRNA#1** |  | **siControl#2** | **PRRX1 siRNA#2** |  |
| COMP | 181.83 | 76.84 | 42.26 | 226.75 | 117.46 | 51.80 |
| NPPB | 92.03 | 10.45 | 11.35 | 54.08 | 27.80 | 51.41 |
| NOX4 | 87.32 | 36.32 | 41.59 | 66.29 | 47.43 | 71.55 |
| ENST00000573315 | 66.91 | 8.42 | 12.58 | 59.96 | 13.83 | 23.07 |
| NPPB | 48.50 | 5.59 | 11.53 | 32.47 | 18.37 | 56.58 |
| IGF1 | 41.21 | 12.97 | 31.47 | 39.15 | 14.68 | 37.50 |
| ELN | 39.80 | 10.85 | 27.26 | 65.16 | 57.14 | 87.69 |
| IGFBP3 | 36.44 | 6.17 | 16.93 | 16.64 | 11.73 | 70.50 |
| ITGA11 | 33.73 | 13.98 | 41.46 | 41.36 | 29.23 | 70.69 |
| TSPAN2 | 29.27 | 6.97 | 23.82 | 25.73 | 21.02 | 81.70 |
| FZD8 | 28.30 | 20.28 | 71.67 | 20.62 | 16.79 | 81.43 |
| SCRG1 | 28.06 | 5.04 | 17.95 | 26.01 | 11.43 | 43.93 |
| GDF6 | 24.92 | 11.94 | 47.91 | 33.72 | 15.22 | 45.12 |
| SLCO2A1 | 24.15 | 34.04 | 140.95 | 40.86 | 16.00 | 39.17 |
| ANKRD1 | 22.65 | 24.48 | 108.08 | 24.10 | 20.21 | 83.84 |
| AMIGO2 | 22.18 | 10.21 | 46.01 | 20.72 | 24.90 | 120.21 |
| SCX | 21.98 | 11.28 | 51.32 | 19.76 | 13.65 | 69.07 |
| MDFI | 21.89 | 4.26 | 19.47 | 18.44 | 14.31 | 77.61 |
| HAPLN1 | 21.83 | 6.13 | 28.08 | 17.97 | 5.47 | 30.44 |
| IL11 | 21.82 | 17.89 | 82.03 | 28.36 | 43.26 | 152.53 |
| SERTAD4 | 19.91 | 10.82 | 54.36 | 36.86 | 17.42 | 47.26 |
| HBEGF | 19.20 | 18.65 | 97.17 | 20.03 | 28.56 | 142.60 |
| PMEPA1 | 18.83 | 15.98 | 84.87 | 18.07 | 14.78 | 81.77 |
| KANK4 | 18.46 | 1.85 | 10.01 | 14.64 | 8.18 | 55.87 |
| CTPS1 | 18.08 | 11.99 | 66.32 | 15.62 | 16.23 | 103.95 |
| ELN | 18.05 | 8.40 | 46.54 | 23.79 | 15.05 | 63.28 |
| PRG4 | 17.89 | 4.65 | 26.02 | 18.10 | 7.58 | 41.90 |
| SERTAD4-AS1 | 17.58 | 13.00 | 73.91 | 32.23 | 24.67 | 76.54 |
| NKX3-1 | 16.94 | 3.47 | 20.51 | 10.40 | 8.59 | 82.63 |
| MEGF6 | 16.78 | 12.62 | 75.24 | 26.04 | 9.22 | 35.40 |
| KANK4 | 16.59 | 1.89 | 11.41 | 12.33 | 6.99 | 56.69 |
| FOXS1 | 16.51 | 7.88 | 47.75 | 7.81 | 9.68 | 124.01 |
| CNN1 | 15.29 | 5.32 | 34.81 | 18.08 | 9.98 | 55.19 |
| LINC01711 | 14.97 | 5.30 | 35.41 | 11.03 | 8.17 | 74.09 |
| LINC02593 | 14.45 | 10.38 | 71.82 | 7.71 | 12.13 | 157.34 |
| CTPS1 | 13.65 | 9.96 | 72.93 | 12.21 | 13.11 | 107.33 |
| EGR2 | 13.25 | 14.07 | 106.15 | 12.00 | 20.39 | 169.94 |
| TNFAIP6 | 13.23 | 3.73 | 28.15 | 8.36 | 11.21 | 134.14 |
| DSP | 12.36 | 6.61 | 53.43 | 13.41 | 9.37 | 69.89 |
| PMEPA1 | 12.35 | 12.27 | 99.42 | 13.56 | 13.19 | 97.28 |
| TPM1 | 12.12 | 9.58 | 79.00 | 15.34 | 15.06 | 98.18 |
| IGF1 | 11.88 | 4.53 | 38.12 | 10.33 | 6.47 | 62.68 |
| SERPINE1 | 11.84 | 15.32 | 129.40 | 27.83 | 32.38 | 116.34 |
| FSTL3 | 11.73 | 7.31 | 62.28 | 9.21 | 7.15 | 77.55 |
| BMP6 | 11.69 | 7.11 | 60.81 | 14.99 | 6.88 | 45.88 |
| LOC105377123 | 11.50 | 3.62 | 31.46 | 7.09 | 4.72 | 66.62 |
| COL4A1 | 11.48 | 6.98 | 60.80 | 12.26 | 10.42 | 84.95 |
| LYPD1 | 11.03 | 5.40 | 48.98 | 18.58 | 19.39 | 104.35 |
| TPM1 | 10.94 | 8.90 | 81.31 | 12.16 | 8.30 | 68.28 |
| XRCC4 | 10.53 | 2.90 | 27.53 | 6.90 | 7.18 | 104.08 |
| MFAP5 | 10.50 | 4.18 | 39.77 | 12.88 | 2.57 | 19.97 |
| lnc-RP11-597K23.2.1-2 | 10.49 | 3.40 | 32.40 | 8.06 | 5.14 | 63.80 |
| BHLHE40 | 10.38 | 6.97 | 67.22 | 8.17 | 9.36 | 114.62 |
| LOC105369340 | 10.16 | 3.87 | 38.07 | 5.31 | 2.80 | 52.68 |
| SERPINE2 | 10.06 | 8.52 | 84.72 | 12.50 | 19.06 | 152.44 |
| APCDD1L | 9.97 | 6.06 | 60.82 | 11.82 | 7.74 | 65.49 |
| PTGS1 | 9.96 | 6.40 | 64.27 | 8.43 | 5.19 | 61.49 |
| FGD5-AS1 | 9.73 | 4.18 | 42.98 | 9.86 | 6.94 | 70.39 |
| TPM1 | 9.68 | 7.58 | 78.35 | 13.55 | 8.67 | 63.98 |
| LOC105369205 | 9.47 | 7.01 | 74.07 | 16.54 | 8.60 | 51.97 |
| NUAK1 | 9.28 | 5.41 | 58.30 | 11.50 | 7.23 | 62.91 |
| SYT12 | 9.03 | 12.75 | 141.21 | 10.69 | 13.11 | 122.64 |
| COL10A1 | 8.93 | 3.47 | 38.83 | 11.21 | 9.34 | 83.32 |
| NOX4 | 8.76 | 5.78 | 66.00 | 8.13 | 7.67 | 94.27 |
| NPR3 | 8.66 | 3.10 | 35.74 | 5.36 | 4.96 | 92.44 |
| MXRA5 | 8.66 | 7.98 | 92.12 | 5.67 | 10.20 | 180.08 |
| ENC1 | 8.66 | 3.16 | 36.52 | 6.42 | 8.73 | 135.98 |
| IGFBP7 | 8.60 | 2.96 | 34.43 | 9.07 | 4.97 | 54.82 |
| WRB-SH3BGR | 8.48 | 2.45 | 28.90 | 6.78 | 3.19 | 47.02 |
| ADAM12 | 8.42 | 2.55 | 30.24 | 4.39 | 5.83 | 132.76 |
| IGFBP7 | 8.42 | 3.31 | 39.35 | 8.91 | 4.10 | 45.99 |
| LDLRAD4 | 8.40 | 2.62 | 31.24 | 5.18 | 3.48 | 67.10 |
| LOC107985502 | 8.39 | 8.08 | 96.36 | 6.30 | 11.51 | 182.64 |
| NPR3 | 8.36 | 3.51 | 42.02 | 4.34 | 3.95 | 90.97 |
| ADAM12 | 8.34 | 1.95 | 23.42 | 4.52 | 5.96 | 131.70 |
| GXYLT2 | 8.28 | 2.51 | 30.33 | 6.62 | 5.40 | 81.51 |
| PAWR | 8.23 | 4.36 | 53.04 | 9.09 | 7.74 | 85.11 |
| SEMA7A | 8.18 | 6.23 | 76.15 | 12.96 | 14.15 | 109.18 |
| ACTBL2 | 8.14 | 5.47 | 67.25 | 11.75 | 6.54 | 55.68 |
| CHAC1 | 8.14 | 5.78 | 71.00 | 13.74 | 12.93 | 94.11 |
| NALCN | 8.11 | 4.56 | 56.23 | 5.49 | 6.46 | 117.56 |
| ZNF365 | 8.06 | 5.83 | 72.27 | 7.41 | 8.15 | 109.97 |
| DYSF | 8.03 | 3.12 | 38.83 | 4.32 | 2.69 | 62.30 |
| ASNSP1 | 7.92 | 3.44 | 43.39 | 9.46 | 7.35 | 77.76 |
| XYLT1 | 7.84 | 5.52 | 70.37 | 5.89 | 7.03 | 119.35 |
| LYPD1 | 7.80 | 3.92 | 50.24 | 11.91 | 11.42 | 95.83 |
| ASNSP1 | 7.60 | 3.34 | 43.89 | 9.74 | 8.10 | 83.12 |
| PLCB4 | 7.54 | 2.79 | 37.07 | 6.06 | 5.00 | 82.46 |
| MFAP3L | 7.49 | 3.90 | 52.12 | 8.99 | 4.06 | 45.13 |
| HHIP | 7.36 | 4.23 | 57.44 | 8.46 | 3.45 | 40.76 |
| PLXDC2 | 7.34 | 6.83 | 93.06 | 8.37 | 7.46 | 89.12 |
| PI16 | 7.32 | 2.79 | 38.12 | 7.67 | 5.95 | 77.59 |
| DSP | 7.30 | 3.23 | 44.33 | 6.88 | 5.57 | 80.90 |
| MYH11 | 7.23 | 5.28 | 73.02 | 11.30 | 3.86 | 34.20 |
| MYH11 | 7.21 | 4.85 | 67.29 | 11.48 | 3.84 | 33.48 |
| PLPP4 | 7.18 | 4.30 | 59.92 | 7.77 | 4.28 | 55.11 |
| MTHFD2 | 7.16 | 2.51 | 35.14 | 7.77 | 5.38 | 69.21 |
| A_33_P3368900 | 7.08 | 1.88 | 26.57 | 6.03 | 3.88 | 64.32 |
| PSAT1 | 7.07 | 3.65 | 51.59 | 8.30 | 6.31 | 76.04 |
| AMTN | 7.07 | 1.77 | 25.11 | 6.38 | 3.31 | 51.88 |
| CDH2 | 7.02 | 2.75 | 39.15 | 3.70 | 3.39 | 91.56 |
| ENST00000511103 | 6.94 | 3.88 | 55.82 | 9.41 | 7.92 | 84.15 |
| SPDL1 | 6.94 | 2.86 | 41.27 | 5.23 | 7.67 | 146.86 |
| COL5A1 | 6.91 | 4.13 | 59.85 | 5.59 | 7.97 | 142.45 |
| LOC105369205 | 6.86 | 5.11 | 74.39 | 9.22 | 6.95 | 75.44 |
| HSD17B6 | 6.79 | 3.00 | 44.16 | 8.49 | 7.12 | 83.93 |
| PTHLH | 6.75 | 3.96 | 58.63 | 5.42 | 10.19 | 188.11 |
| ASPN | 6.71 | 2.10 | 31.32 | 8.38 | 4.99 | 59.50 |
| ASNS | 6.70 | 2.92 | 43.68 | 9.01 | 6.75 | 74.98 |
| SYNDIG1 | 6.67 | 3.33 | 50.00 | 4.01 | 4.76 | 118.65 |
| ELFN2 | 6.67 | 4.39 | 65.85 | 7.14 | 3.95 | 55.35 |
| PLOD2 | 6.66 | 3.54 | 53.23 | 4.01 | 7.92 | 197.64 |
| EFR3B | 6.66 | 5.28 | 79.29 | 4.76 | 6.41 | 134.69 |
| lnc-CABLES1-1 | 6.63 | 0.99 | 14.92 | 5.35 | 1.60 | 29.85 |
| NCF2 | 6.58 | 4.05 | 61.56 | 6.01 | 5.05 | 84.05 |
| GFRA1 | 6.53 | 3.41 | 52.27 | 6.86 | 3.90 | 56.88 |
| CEMIP2 | 6.49 | 2.41 | 37.16 | 5.05 | 6.74 | 133.53 |
| GADD45B | 6.49 | 12.37 | 190.64 | 9.88 | 12.23 | 123.75 |
| TES | 6.46 | 3.89 | 60.14 | 6.14 | 3.21 | 52.36 |
| MCAM | 6.45 | 2.78 | 43.10 | 6.85 | 3.74 | 54.57 |
| CRLF1 | 6.40 | 5.43 | 84.78 | 7.24 | 5.26 | 72.65 |
| ACTA2 | 6.38 | 7.65 | 119.88 | 11.69 | 7.65 | 65.43 |
| ENST00000518968 | 6.38 | 3.27 | 51.19 | 8.51 | 7.42 | 87.20 |
| NXPH4 | 6.30 | 6.13 | 97.34 | 6.74 | 6.20 | 92.01 |
| ACTG2 | 6.29 | 7.34 | 116.78 | 13.67 | 9.72 | 71.12 |
| ATP10A | 6.27 | 7.38 | 117.64 | 7.69 | 8.97 | 116.76 |
| A_33_P3255824 | 6.22 | 5.35 | 85.95 | 4.85 | 6.86 | 141.52 |
| CHAC1 | 6.21 | 5.52 | 88.88 | 9.54 | 8.96 | 93.95 |
| COL4A2 | 6.18 | 3.92 | 63.38 | 4.77 | 3.81 | 79.96 |
| ELN-AS1 | 6.11 | 3.56 | 58.16 | 7.39 | 4.67 | 63.25 |
| LIF | 5.93 | 6.14 | 103.54 | 7.15 | 8.07 | 112.93 |
| ADM2 | 5.89 | 4.33 | 73.51 | 8.06 | 5.67 | 70.38 |
| LOX | 5.85 | 3.50 | 59.80 | 6.42 | 5.03 | 78.34 |
| LINC01614 | 5.85 | 1.39 | 23.82 | 2.05 | 4.32 | 210.69 |
| UCK2 | 5.84 | 4.63 | 79.21 | 5.76 | 6.88 | 119.51 |
| ISLR2 | 5.84 | 3.08 | 52.68 | 3.89 | 3.13 | 80.48 |
| TNFSF4 | 5.80 | 4.43 | 76.38 | 10.15 | 3.97 | 39.10 |
| ACKR3 | 5.74 | 2.48 | 43.13 | 3.64 | 2.78 | 76.49 |
| CTH | 5.72 | 2.88 | 50.35 | 5.52 | 4.30 | 77.76 |
| SLC7A1 | 5.71 | 3.54 | 62.05 | 7.88 | 7.75 | 98.30 |
| CDH2 | 5.64 | 2.12 | 37.62 | 2.98 | 2.72 | 91.12 |
| DUSP26 | 5.63 | 1.38 | 24.62 | 5.58 | 2.22 | 39.76 |
| OXTR | 5.62 | 3.02 | 53.67 | 4.13 | 5.66 | 137.21 |
| PLCB4 | 5.61 | 2.56 | 45.52 | 5.17 | 3.60 | 69.69 |
| AMZ1 | 5.56 | 7.71 | 138.73 | 4.63 | 4.30 | 92.68 |
| EDN1 | 5.53 | 6.36 | 115.01 | 19.43 | 15.04 | 77.40 |
| EDIL3 | 5.52 | 2.02 | 36.55 | 4.41 | 3.96 | 89.86 |
| LF212376 | 5.51 | 2.73 | 49.50 | 5.62 | 3.31 | 58.98 |
| KCNN4 | 5.43 | 5.62 | 103.59 | 7.22 | 3.19 | 44.15 |
| HHIP | 5.39 | 2.87 | 53.15 | 5.69 | 2.98 | 52.40 |
| HSPA13 | 5.38 | 2.59 | 48.12 | 4.81 | 4.97 | 103.30 |
| GALNT18 | 5.38 | 3.70 | 68.81 | 7.15 | 4.21 | 58.84 |
| SPDL1 | 5.37 | 2.61 | 48.65 | 5.07 | 6.68 | 131.62 |
| FGF1 | 5.35 | 4.68 | 87.46 | 7.23 | 5.49 | 75.88 |
| LOC101928076 | 5.35 | 3.72 | 69.51 | 4.43 | 3.36 | 75.76 |
| TNS1 | 5.34 | 4.79 | 89.79 | 4.89 | 3.51 | 71.74 |
| UNC5B | 5.32 | 2.45 | 46.09 | 5.57 | 4.49 | 80.68 |
| ASPN | 5.32 | 1.72 | 32.28 | 5.60 | 4.44 | 79.35 |
| SPOCK1 | 5.30 | 3.95 | 74.52 | 4.92 | 3.92 | 79.65 |
| PRPS1L1 | 5.29 | 3.66 | 69.24 | 4.40 | 4.07 | 92.50 |
| SGCG | 5.27 | 2.71 | 51.39 | 9.71 | 3.18 | 32.79 |
| ADAMTS6 | 5.27 | 3.40 | 64.48 | 4.72 | 4.39 | 93.02 |
| MSMO1 | 5.23 | 2.60 | 49.77 | 4.06 | 4.05 | 99.80 |
| RFLNB | 5.22 | 3.44 | 65.90 | 4.16 | 4.25 | 102.11 |
| COL5A1 | 5.16 | 3.15 | 61.12 | 5.11 | 5.26 | 102.99 |
| PLN | 5.15 | 3.28 | 63.64 | 5.62 | 4.18 | 74.28 |
| LIMS2 | 5.14 | 3.37 | 65.71 | 7.50 | 4.15 | 55.34 |
| LOC105379057 | 5.08 | 3.36 | 66.03 | 5.98 | 4.11 | 68.80 |
| PCED1B | 5.07 | 4.06 | 80.09 | 5.43 | 4.90 | 90.27 |
| LOC105369340 | 5.06 | 3.77 | 74.49 | 4.95 | 2.41 | 48.63 |
| CCN2 | 5.06 | 4.52 | 89.37 | 9.73 | 8.96 | 92.05 |
| SIK1 | 5.04 | 4.34 | 86.06 | 4.06 | 7.46 | 183.85 |
| KLHDC7B | 5.02 | 3.79 | 75.43 | 4.44 | 4.46 | 100.34 |
| OSBPL10 | 5.00 | 3.82 | 76.43 | 6.27 | 4.93 | 78.57 |
| LINC01605 | 4.99 | 4.28 | 85.81 | 7.13 | 7.83 | 109.85 |
| ANXA8L1 | 4.97 | 2.73 | 55.03 | 3.09 | 1.82 | 59.01 |
| LOC105379010 | 4.97 | 2.01 | 40.42 | 3.62 | 2.29 | 63.39 |
| ALPK2 | 4.96 | 3.35 | 67.52 | 5.19 | 5.51 | 106.09 |
| MRAS | 4.95 | 5.16 | 104.25 | 4.63 | 4.66 | 100.69 |
| MYOSLID | 4.94 | 6.93 | 140.21 | 5.67 | 6.12 | 107.88 |
| KRT80 | 4.94 | 3.80 | 76.91 | 4.36 | 5.55 | 127.43 |
| ATP1B1 | 4.94 | 1.59 | 32.16 | 3.14 | 2.53 | 80.63 |
| SKIL | 4.92 | 2.57 | 52.14 | 3.51 | 5.40 | 153.77 |
| GALNT10 | 4.91 | 3.37 | 68.73 | 3.57 | 2.92 | 81.97 |
| LINC01013 | 4.89 | 3.34 | 68.22 | 5.64 | 3.21 | 56.93 |
| PROC | 4.89 | 8.23 | 168.46 | 2.95 | 7.15 | 242.19 |
| LOC105370706 | 4.87 | 2.67 | 54.87 | 3.56 | 1.93 | 54.27 |
| TUFT1 | 4.84 | 3.68 | 76.01 | 5.98 | 7.04 | 117.62 |
| PDLIM5 | 4.83 | 3.04 | 62.82 | 4.20 | 4.04 | 96.33 |
| TAGLN | 4.83 | 6.87 | 142.16 | 13.10 | 8.96 | 68.41 |
| ACTG2 | 4.83 | 3.20 | 66.32 | 4.28 | 7.49 | 175.08 |
| LINC01638 | 4.80 | 3.34 | 69.54 | 3.12 | 2.71 | 86.89 |
| PRPS1 | 4.80 | 3.40 | 70.94 | 4.38 | 3.76 | 85.65 |
| SMCO4 | 4.78 | 3.68 | 76.93 | 6.02 | 5.35 | 88.84 |
| KCNG1 | 4.77 | 4.29 | 89.87 | 7.12 | 6.62 | 93.05 |
| RUNX1 | 4.77 | 3.08 | 64.42 | 4.24 | 5.07 | 119.52 |
| POSTN | 4.77 | 2.39 | 50.13 | 5.68 | 9.83 | 172.89 |
| FLJ43315 | 4.77 | 2.48 | 51.94 | 5.31 | 4.51 | 84.95 |
| LINC00842 | 4.76 | 3.28 | 68.78 | 6.24 | 3.71 | 59.40 |
| ALDH1L2 | 4.75 | 1.89 | 39.75 | 4.37 | 3.74 | 85.52 |
| CALD1 | 4.75 | 3.32 | 69.85 | 4.12 | 4.34 | 105.33 |
| SUSD2 | 4.75 | 3.02 | 63.61 | 10.76 | 4.51 | 41.95 |
| DYNLT3 | 4.72 | 2.56 | 54.32 | 3.12 | 3.96 | 127.17 |
| KRT18 | 4.72 | 3.05 | 64.65 | 5.12 | 4.33 | 84.62 |
| UAP1 | 4.71 | 3.21 | 68.12 | 5.44 | 5.45 | 100.19 |
| DMD | 4.67 | 2.57 | 54.94 | 4.71 | 5.21 | 110.62 |
| ALDH1B1 | 4.67 | 3.61 | 77.28 | 4.72 | 3.66 | 77.53 |
| PRPS1 | 4.66 | 2.39 | 51.33 | 4.56 | 4.23 | 92.78 |
| SLC7A5 | 4.63 | 3.53 | 76.08 | 3.57 | 5.64 | 158.08 |
| ITGBL1 | 4.63 | 3.50 | 75.70 | 4.59 | 3.91 | 85.25 |
| CILP | 4.61 | 1.69 | 36.69 | 5.25 | 2.92 | 55.66 |
| ADAM19 | 4.60 | 3.07 | 66.80 | 5.21 | 5.64 | 108.37 |
| EIF4EBP1 | 4.60 | 3.47 | 75.45 | 7.33 | 4.66 | 63.58 |
| ADAMTS6 | 4.60 | 2.81 | 61.15 | 4.46 | 3.58 | 80.25 |
| IGFBP7 | 4.57 | 2.30 | 50.25 | 4.57 | 3.14 | 68.63 |
| MYOSLID | 4.57 | 6.78 | 148.27 | 5.60 | 6.00 | 107.23 |
| LINC01969 | 4.57 | 3.72 | 81.50 | 3.27 | 3.61 | 110.26 |
| SLC17A9 | 4.56 | 4.62 | 101.40 | 3.95 | 4.55 | 115.25 |
| TRIB3 | 4.55 | 2.41 | 52.92 | 5.68 | 5.35 | 94.12 |
| SEC11C | 4.53 | 2.64 | 58.35 | 3.76 | 3.08 | 81.85 |
| SRRM3 | 4.53 | 4.75 | 104.86 | 6.51 | 3.72 | 57.06 |
| DIAPH3 | 4.53 | 1.70 | 37.63 | 4.05 | 2.98 | 73.60 |
| QPCT | 4.51 | 1.54 | 34.20 | 3.19 | 2.90 | 90.90 |
| GLS | 4.50 | 1.56 | 34.64 | 2.22 | 2.04 | 92.15 |
| WNT5B | 4.49 | 3.84 | 85.53 | 4.78 | 4.58 | 95.72 |
| MATN3 | 4.48 | 2.26 | 50.46 | 4.79 | 4.11 | 85.80 |
| LINC02407 | 4.47 | 5.85 | 130.89 | 6.60 | 7.00 | 106.09 |
| C1QTNF3 | 4.47 | 1.28 | 28.53 | 2.06 | 1.82 | 88.36 |
| SNAR-F | 4.46 | 3.18 | 71.33 | 4.82 | 3.68 | 76.34 |
| CHST6 | 4.46 | 3.47 | 77.79 | 3.27 | 3.94 | 120.30 |
| TAGLN | 4.46 | 6.83 | 153.28 | 13.19 | 8.36 | 63.41 |
| ENST00000439132 | 4.45 | 2.49 | 55.99 | 5.46 | 5.52 | 101.07 |
| TXNDC5 | 4.42 | 1.80 | 40.80 | 3.65 | 4.72 | 129.09 |
| ITGBL1 | 4.42 | 3.69 | 83.68 | 5.17 | 4.12 | 79.70 |
| MMP24 | 4.40 | 2.72 | 61.86 | 4.81 | 2.71 | 56.29 |
| THUMPD2 | 4.39 | 3.72 | 84.71 | 3.13 | 4.06 | 129.84 |
| CAMK1D | 4.39 | 3.25 | 74.12 | 5.75 | 4.37 | 75.91 |
| HES6 | 4.38 | 5.32 | 121.36 | 6.44 | 4.53 | 70.38 |
| MLLT11 | 4.38 | 3.73 | 85.14 | 5.18 | 3.30 | 63.70 |
| THBS2 | 4.38 | 4.40 | 100.33 | 4.48 | 4.17 | 93.03 |
| ADAM12 | 4.38 | 2.53 | 57.71 | 3.05 | 4.98 | 163.09 |
| GLS | 4.37 | 2.14 | 48.94 | 2.61 | 2.00 | 76.75 |
| THC2642375 | 4.37 | 3.73 | 85.52 | 3.34 | 3.05 | 91.28 |
| SH3PXD2A | 4.37 | 3.58 | 81.97 | 3.94 | 4.04 | 102.74 |
| SH3PXD2A | 4.36 | 2.75 | 63.20 | 3.54 | 3.26 | 92.08 |
| TMC7 | 4.35 | 3.01 | 69.23 | 2.89 | 3.02 | 104.54 |
| RAI14 | 4.33 | 3.20 | 73.92 | 4.28 | 6.15 | 143.74 |
| GLS | 4.31 | 2.23 | 51.89 | 2.99 | 5.21 | 174.06 |
| KRT18 | 4.30 | 2.96 | 68.88 | 5.67 | 4.47 | 78.83 |
| DACT1 | 4.29 | 2.29 | 53.52 | 3.28 | 4.19 | 127.82 |
| SNAR-G2 | 4.28 | 2.77 | 64.69 | 4.25 | 3.30 | 77.70 |
| CLIC4 | 4.28 | 2.87 | 67.00 | 4.53 | 3.63 | 80.06 |
| KRT17 | 4.28 | 2.85 | 66.59 | 5.15 | 2.96 | 57.35 |
| INHBA | 4.27 | 3.16 | 74.01 | 3.27 | 4.63 | 141.40 |
| RTKN2 | 4.27 | 2.69 | 63.03 | 5.48 | 5.08 | 92.79 |
| SAMD11 | 4.26 | 3.80 | 89.21 | 3.42 | 4.12 | 120.31 |
| ABCA3 | 4.25 | 2.53 | 59.60 | 4.48 | 3.52 | 78.49 |
| GLIPR2 | 4.23 | 2.74 | 64.65 | 4.78 | 3.10 | 64.91 |
| PTPRN | 4.23 | 2.28 | 53.78 | 5.33 | 2.32 | 43.48 |
| PDLIM3 | 4.22 | 2.29 | 54.28 | 5.09 | 4.58 | 90.14 |
| CDH6 | 4.22 | 3.46 | 81.96 | 3.24 | 6.39 | 197.23 |
| TSPAN13 | 4.21 | 1.70 | 40.41 | 4.72 | 3.16 | 66.88 |
| DYNLT3 | 4.20 | 2.21 | 52.62 | 2.81 | 3.48 | 123.70 |
| PXDC1 | 4.20 | 4.48 | 106.58 | 4.75 | 4.90 | 103.06 |
| RBP1 | 4.19 | 2.54 | 60.65 | 5.18 | 2.25 | 43.41 |
| SNAR-D | 4.17 | 2.33 | 55.73 | 3.84 | 2.87 | 74.65 |
| LINC01583 | 4.16 | 2.29 | 55.10 | 3.36 | 1.92 | 57.25 |
| SNAR-H | 4.15 | 2.53 | 60.95 | 3.89 | 3.17 | 81.38 |
| lnc-CALHM3-2 | 4.15 | 4.01 | 96.51 | 2.98 | 2.65 | 89.06 |
| LINC00592 | 4.15 | 3.26 | 78.50 | 4.25 | 4.28 | 100.64 |
| PCK2 | 4.14 | 2.84 | 68.72 | 5.77 | 3.00 | 51.98 |
| SORCS2 | 4.14 | 3.81 | 91.98 | 3.76 | 4.55 | 120.94 |
| LINC01638 | 4.13 | 2.76 | 66.88 | 2.79 | 2.92 | 104.71 |
| PPP1R13L | 4.12 | 6.01 | 145.86 | 5.00 | 4.08 | 81.56 |
| TNFRSF12A | 4.12 | 3.63 | 88.13 | 4.88 | 6.17 | 126.61 |
| AFP | 4.12 | 2.52 | 61.14 | 2.60 | 2.79 | 107.45 |
| CENPN | 4.11 | 2.85 | 69.28 | 3.27 | 2.44 | 74.72 |
| SNAR-B2 | 4.11 | 2.21 | 53.73 | 3.97 | 2.98 | 75.03 |
| NFKBIZ | 4.10 | 2.22 | 54.19 | 3.23 | 3.35 | 103.60 |
| LAMP3 | 4.07 | 3.45 | 84.90 | 8.29 | 3.54 | 42.65 |
| ADAM12 | 4.06 | 2.03 | 50.07 | 3.10 | 3.13 | 100.79 |
| EPRS | 4.06 | 1.78 | 43.79 | 2.90 | 3.41 | 117.72 |
| SLC39A14 | 4.05 | 2.67 | 65.93 | 2.57 | 3.39 | 131.78 |
| LMO7 | 4.05 | 2.49 | 61.46 | 5.88 | 5.58 | 94.79 |
| CLEC18B | 4.05 | 1.42 | 34.98 | 3.78 | 1.61 | 42.61 |
| AK4 | 4.03 | 1.96 | 48.50 | 2.37 | 3.07 | 129.66 |
| CLTCL1 | 4.03 | 2.61 | 64.84 | 2.84 | 2.21 | 77.81 |
| QPCT | 4.02 | 1.51 | 37.59 | 3.51 | -0.33 | 0.00 |
| HHAT | 4.02 | 2.29 | 56.95 | 4.63 | -0.43 | 0.00 |
| CMKLR1 | 4.01 | 3.51 | 87.55 | 5.51 | -0.19 | 0.00 |
| GLI1 | 4.01 | 1.93 | 48.22 | 2.66 | -0.40 | 0.00 |
| DHX58 | -3.99 | -2.30 | 57.72 | -3.54 | -3.07 | 0.00 |
| ALDH3A2 | -3.99 | -2.12 | 53.18 | -5.24 | -3.76 | 71.73 |
| HLA-DMB | -4.00 | -3.40 | 84.97 | -5.31 | -3.73 | 70.24 |
| TYMSOS | -4.01 | -2.46 | 61.47 | -4.43 | -3.72 | 83.99 |
| PCDH18 | -4.02 | -2.08 | 51.81 | -3.33 | -3.21 | 96.31 |
| ZNRF1 | -4.02 | -1.83 | 45.58 | -3.59 | -3.30 | 91.92 |
| CD36 | -4.02 | -4.27 | 106.09 | -7.47 | -2.72 | 36.43 |
| C1S | -4.03 | -1.10 | 27.25 | -2.53 | -2.75 | 108.91 |
| PGM5P4-AS1 | -4.03 | -1.65 | 41.05 | -2.63 | -3.94 | 149.98 |
| LTBP4 | -4.03 | -1.53 | 37.87 | -3.06 | -4.40 | 143.44 |
| HDAC5 | -4.04 | -2.26 | 55.96 | -3.21 | -3.41 | 106.36 |
| TLE1 | -4.04 | -1.88 | 46.40 | -3.28 | -2.96 | 90.32 |
| VAT1L | -4.05 | -2.96 | 73.09 | -3.05 | -2.88 | 94.56 |
| AHNAK2 | -4.05 | -2.23 | 54.96 | -3.88 | -6.34 | 163.34 |
| P2RX7 | -4.05 | -1.99 | 49.11 | -2.47 | -2.43 | 98.39 |
| LINC01315 | -4.06 | -2.44 | 60.06 | -6.08 | -2.92 | 48.05 |
| PPARGC1A | -4.06 | -4.99 | 123.05 | -6.07 | -4.47 | 73.62 |
| SERPING1 | -4.08 | -1.43 | 34.98 | -2.47 | -2.70 | 109.21 |
| CYP3A7 | -4.08 | -3.01 | 73.77 | -4.48 | -2.55 | 57.04 |
| VEGFD | -4.08 | -5.31 | 130.02 | -4.26 | -3.09 | 72.51 |
| SGSH | -4.09 | -1.83 | 44.80 | -3.89 | -3.39 | 87.09 |
| ACKR4 | -4.09 | -2.02 | 49.34 | -4.22 | -1.48 | 35.08 |
| ADRB2 | -4.11 | -2.02 | 49.05 | -3.44 | -3.11 | 90.32 |
| RGS2 | -4.12 | -3.18 | 77.13 | -3.75 | -1.82 | 48.57 |
| SERPING1 | -4.13 | -1.14 | 27.50 | -2.24 | -2.12 | 94.85 |
| TNXB | -4.13 | -1.77 | 42.85 | -4.52 | -3.19 | 70.56 |
| FBLN1 | -4.13 | -1.65 | 39.99 | -4.82 | -3.40 | 70.53 |
| COL4A6 | -4.13 | -1.89 | 45.63 | -4.46 | -3.24 | 72.74 |
| MAST4 | -4.14 | -2.17 | 52.35 | -3.55 | -3.31 | 93.29 |
| AQP3 | -4.14 | -2.82 | 68.17 | -5.39 | -3.68 | 68.22 |
| FAM43A | -4.15 | -2.11 | 50.79 | -2.55 | -3.40 | 133.59 |
| COL13A1 | -4.15 | -2.24 | 53.85 | -3.95 | -4.13 | 104.61 |
| AKR1C3 | -4.16 | -2.19 | 52.67 | -4.54 | -3.31 | 72.75 |
| MX1 | -4.16 | -1.03 | 24.88 | -2.65 | -1.74 | 65.73 |
| TFPI | -4.16 | -2.25 | 53.98 | -3.97 | -2.59 | 65.18 |
| RASSF5 | -4.17 | -2.45 | 58.71 | -3.89 | -3.96 | 101.84 |
| CEMIP | -4.18 | -1.50 | 35.95 | -3.56 | -1.61 | 45.27 |
| STAR | -4.18 | -3.10 | 74.32 | -4.38 | -4.56 | 104.00 |
| CIT | -4.18 | -3.08 | 73.69 | -3.90 | -3.78 | 96.95 |
| CD302 | -4.18 | -1.92 | 45.79 | -5.47 | -2.88 | 52.68 |
| HLA-DMA | -4.19 | -1.18 | 28.30 | -3.73 | -4.29 | 115.02 |
| IFIT3 | -4.19 | -2.04 | 48.77 | -3.50 | -2.44 | 69.75 |
| BTN3A2 | -4.19 | -1.98 | 47.25 | -4.10 | -3.61 | 88.15 |
| HMOX1 | -4.20 | -1.41 | 33.63 | -4.12 | -2.56 | 62.20 |
| ACP5 | -4.21 | -2.28 | 54.13 | -4.66 | -2.81 | 60.27 |
| LYNX1 | -4.21 | -2.29 | 54.40 | -3.81 | -3.80 | 99.64 |
| ADSSL1 | -4.23 | -2.30 | 54.37 | -4.15 | -3.15 | 75.83 |
| ABCC6 | -4.24 | -3.54 | 83.65 | -6.40 | -4.19 | 65.44 |
| HACD4 | -4.24 | -2.85 | 67.37 | -4.06 | -3.73 | 91.89 |
| CDHR3 | -4.24 | -2.43 | 57.23 | -3.92 | -2.88 | 73.27 |
| TNS2 | -4.25 | -2.10 | 49.41 | -3.40 | -2.89 | 85.09 |
| IGFBP5 | -4.25 | -1.90 | 44.72 | -2.36 | -2.96 | 125.40 |
| PLA2G4C | -4.26 | -2.52 | 59.16 | -4.52 | -5.20 | 115.15 |
| SLC7A8 | -4.31 | -2.65 | 61.62 | -2.56 | -3.31 | 128.90 |
| KCTD12 | -4.32 | -4.49 | 104.04 | -3.82 | -4.49 | 117.32 |
| GCHFR | -4.32 | -2.61 | 60.47 | -2.76 | -2.57 | 93.27 |
| DHRS13 | -4.32 | -3.23 | 74.74 | -3.10 | -3.86 | 124.63 |
| SOD3 | -4.34 | -1.88 | 43.40 | -4.02 | -2.90 | 72.00 |
| SBSN | -4.34 | -2.62 | 60.27 | -4.41 | -2.32 | 52.65 |
| CU678501 | -4.35 | -1.92 | 44.11 | -4.02 | -3.54 | 88.20 |
| IMPA2 | -4.36 | -1.84 | 42.30 | -2.45 | -2.78 | 113.75 |
| AK5 | -4.36 | -2.25 | 51.65 | -4.00 | -3.77 | 94.13 |
| ANO3 | -4.40 | -2.17 | 49.22 | -4.07 | -2.59 | 63.78 |
| CDC25B | -4.40 | -2.15 | 48.92 | -4.10 | -3.91 | 95.52 |
| LOC101927809 | -4.41 | -2.50 | 56.75 | -3.29 | -3.02 | 91.78 |
| MT1X | -4.41 | -2.49 | 56.53 | -5.03 | -4.03 | 80.20 |
| FER1L4 | -4.44 | -2.54 | 57.33 | -3.80 | -4.19 | 110.14 |
| HLA-DMA | -4.44 | -2.02 | 45.42 | -4.87 | -4.39 | 90.01 |
| PCDH18 | -4.46 | -2.36 | 52.95 | -3.11 | -2.72 | 87.28 |
| RAC2 | -4.46 | -2.03 | 45.45 | -3.28 | -2.94 | 89.48 |
| LPAR1 | -4.46 | -2.11 | 47.25 | -2.98 | -3.01 | 100.83 |
| SERPINF1 | -4.47 | -1.54 | 34.35 | -3.93 | -2.67 | 67.93 |
| SLC27A3 | -4.49 | -2.76 | 61.53 | -3.47 | -5.42 | 156.41 |
| ITGB8 | -4.49 | -2.15 | 47.85 | -4.99 | -2.69 | 53.81 |
| THBD | -4.51 | -4.03 | 89.35 | -3.78 | -3.84 | 101.82 |
| CATSPERZ | -4.51 | -4.00 | 88.70 | -6.82 | -4.69 | 68.75 |
| LTBP4 | -4.57 | -1.86 | 40.66 | -3.67 | -3.42 | 93.26 |
| C16orf89 | -4.57 | -2.66 | 58.07 | -5.42 | -4.49 | 82.87 |
| GAPLINC | -4.58 | -3.67 | 80.30 | -5.09 | -3.68 | 72.29 |
| CCDC102B | -4.59 | -3.99 | 86.90 | -5.78 | -3.11 | 53.75 |
| TNXB | -4.63 | -2.20 | 47.41 | -6.57 | -3.94 | 59.90 |
| S1PR2 | -4.64 | -2.18 | 47.04 | -4.88 | -3.60 | 73.67 |
| ITPKB | -4.65 | -2.50 | 53.70 | -3.42 | -3.40 | 99.26 |
| UBA7 | -4.65 | -1.69 | 36.40 | -3.46 | -3.09 | 89.11 |
| H19 | -4.67 | -1.41 | 30.19 | -4.00 | -3.16 | 79.12 |
| CCR10 | -4.67 | -3.19 | 68.35 | -4.24 | -4.28 | 101.13 |
| BDKRB1 | -4.67 | -2.68 | 57.35 | -3.19 | -2.01 | 63.03 |
| IL6R | -4.68 | -2.41 | 51.43 | -7.17 | -4.62 | 64.44 |
| LSAMP | -4.68 | -1.89 | 40.40 | -3.58 | -2.12 | 59.37 |
| COL14A1 | -4.68 | -3.20 | 68.31 | -6.26 | -3.07 | 49.12 |
| PTN | -4.69 | -1.68 | 35.77 | -5.81 | -1.85 | 31.87 |
| FXYD1 | -4.70 | -1.76 | 37.41 | -3.44 | -3.16 | 91.81 |
| SHC3 | -4.70 | -1.92 | 40.73 | -4.88 | -2.18 | 44.77 |
| KLHL41 | -4.71 | -4.71 | 99.90 | -4.15 | -3.33 | 80.29 |
| lnc-GMDS-3 | -4.81 | -3.47 | 72.06 | -5.06 | -4.04 | 79.90 |
| TM4SF1 | -4.82 | -2.56 | 53.05 | -4.64 | -3.47 | 74.93 |
| NR1H3 | -4.84 | -2.73 | 56.27 | -6.20 | -4.58 | 73.83 |
| PLEKHO2 | -4.87 | -1.91 | 39.29 | -3.86 | -4.01 | 103.68 |
| MBP | -4.89 | -1.88 | 38.49 | -4.71 | -2.93 | 62.09 |
| SIPA1L2 | -4.90 | -2.15 | 43.97 | -4.61 | -2.20 | 47.81 |
| CD302 | -4.90 | -2.29 | 46.81 | -5.88 | -2.32 | 39.45 |
| TBX2 | -4.91 | -2.34 | 47.73 | -4.17 | -3.31 | 79.29 |
| RAB7B | -4.91 | -2.92 | 59.47 | -4.82 | -4.54 | 94.16 |
| ZNF436-AS1 | -4.91 | -2.94 | 59.86 | -4.99 | -3.99 | 79.80 |
| lnc-MRGPRF-1 | -4.91 | -2.89 | 58.74 | -5.18 | -4.32 | 83.31 |
| GUCY1B1 | -4.92 | -5.10 | 103.63 | -4.54 | -2.62 | 57.75 |
| BAALC | -4.93 | -2.50 | 50.84 | -4.31 | -2.94 | 68.11 |
| SCN4B | -4.94 | -3.98 | 80.74 | -3.77 | -4.07 | 107.96 |
| CYP27A1 | -4.95 | -2.55 | 51.44 | -5.47 | -3.36 | 61.48 |
| C16orf74 | -4.97 | -3.67 | 73.89 | -4.42 | -3.84 | 87.05 |
| TNXB | -4.98 | -1.96 | 39.27 | -5.27 | -4.09 | 77.55 |
| LAMA4 | -4.99 | -3.00 | 60.24 | -3.88 | -3.70 | 95.27 |
| PDK4 | -4.99 | -6.37 | 127.74 | -5.35 | -2.62 | 48.89 |
| SLITRK6 | -5.00 | -5.26 | 105.24 | -6.17 | -7.93 | 128.44 |
| ANGPTL2 | -5.01 | -2.60 | 51.76 | -6.35 | -3.46 | 54.45 |
| TMTC1 | -5.02 | -0.71 | 14.12 | -3.35 | -1.97 | 58.81 |
| LAMA4 | -5.02 | -2.82 | 56.17 | -5.04 | -4.10 | 81.37 |
| SNED1 | -5.03 | -1.95 | 38.86 | -4.81 | -2.82 | 58.62 |
| BMP2 | -5.03 | -2.08 | 41.29 | -4.48 | -2.65 | 59.15 |
| PLEKHG4 | -5.03 | -1.90 | 37.78 | -3.45 | -3.19 | 92.33 |
| PPARG | -5.04 | -2.13 | 42.18 | -4.57 | -3.78 | 82.71 |
| IGFBP4 | -5.05 | -2.12 | 42.01 | -2.99 | -2.31 | 77.30 |
| PKDCC | -5.08 | -2.47 | 48.72 | -5.63 | -5.09 | 90.47 |
| KCNK2 | -5.09 | -2.35 | 46.17 | -4.76 | -2.40 | 50.57 |
| DPP4 | -5.12 | -2.12 | 41.33 | -6.18 | -1.66 | 26.92 |
| PKDCC | -5.14 | -2.59 | 50.45 | -4.85 | -4.33 | 89.17 |
| HS6ST1 | -5.14 | -3.21 | 62.36 | -5.85 | -4.25 | 72.68 |
| PYCARD | -5.16 | -2.97 | 57.54 | -3.70 | -3.53 | 95.38 |
| CASP1 | -5.16 | -2.89 | 55.92 | -5.53 | -2.64 | 47.70 |
| FENDRR | -5.19 | -2.11 | 40.78 | -4.41 | -3.17 | 71.86 |
| LCE2C | -5.19 | -4.87 | 93.99 | -13.39 | -3.07 | 22.93 |
| ZNF395 | -5.19 | -2.16 | 41.62 | -5.09 | -3.81 | 74.86 |
| CAMK2N1 | -5.19 | -2.39 | 46.09 | -3.99 | -3.65 | 91.46 |
| PRRT2 | -5.20 | -5.60 | 107.75 | -6.13 | -6.21 | 101.33 |
| HGF | -5.20 | -1.81 | 34.71 | -3.86 | -1.72 | 44.55 |
| QSOX1 | -5.21 | -1.30 | 25.01 | -3.88 | -2.99 | 77.07 |
| RNA5-8SN5 | -5.21 | -3.08 | 59.14 | -8.32 | -14.95 | 179.63 |
| FMO3 | -5.21 | -3.44 | 66.07 | -4.00 | -3.03 | 75.72 |
| C4B | -5.22 | -2.03 | 38.94 | -3.10 | -2.31 | 74.37 |
| NR2F1 | -5.24 | -3.33 | 63.58 | -5.57 | -4.54 | 81.59 |
| BMPER | -5.24 | -2.92 | 55.63 | -3.52 | -3.12 | 88.50 |
| FENDRR | -5.25 | -1.92 | 36.64 | -4.48 | -2.80 | 62.46 |
| CA11 | -5.26 | -2.39 | 45.42 | -3.55 | -3.30 | 93.05 |
| CHI3L2 | -5.27 | -2.94 | 55.72 | -4.97 | -3.26 | 65.51 |
| PDE7B | -5.29 | -4.02 | 75.98 | -4.67 | -3.11 | 66.56 |
| SPIRE2 | -5.30 | -2.65 | 50.01 | -4.93 | -4.21 | 85.49 |
| SLC15A3 | -5.31 | -1.95 | 36.66 | -3.70 | -2.92 | 78.85 |
| S100A4 | -5.32 | -2.75 | 51.75 | -3.89 | -3.33 | 85.55 |
| ISYNA1 | -5.35 | -2.15 | 40.13 | -3.27 | -2.46 | 75.19 |
| PSMB9 | -5.36 | -2.73 | 50.96 | -4.05 | -3.48 | 85.71 |
| IL33 | -5.41 | -3.16 | 58.37 | -6.88 | -1.88 | 27.35 |
| PLEKHG4 | -5.44 | -2.43 | 44.69 | -3.92 | -3.38 | 86.24 |
| CLDN23 | -5.44 | -3.47 | 63.79 | -6.36 | -5.02 | 79.00 |
| QSOX1 | -5.52 | -1.39 | 25.24 | -3.70 | -2.80 | 75.84 |
| OSR2 | -5.55 | -4.71 | 84.91 | -4.86 | -7.07 | 145.49 |
| BATF2 | -5.55 | -2.47 | 44.42 | -5.58 | -3.65 | 65.33 |
| ANKRD33B | -5.63 | -3.27 | 58.14 | -7.53 | -5.29 | 70.26 |
| H19 | -5.63 | -1.43 | 25.30 | -4.86 | -3.57 | 73.33 |
| GAPLINC | -5.66 | -4.07 | 71.96 | -5.52 | -4.31 | 77.98 |
| BDKRB2 | -5.66 | -4.10 | 72.42 | -5.19 | -4.07 | 78.40 |
| AKAP12 | -5.68 | -2.15 | 37.80 | -3.32 | -2.00 | 60.16 |
| NEURL1B | -5.72 | -4.41 | 77.16 | -4.22 | -4.79 | 113.70 |
| DAPK2 | -5.73 | -1.97 | 34.42 | -2.26 | -2.21 | 97.77 |
| S1PR1 | -5.73 | -6.43 | 112.28 | -6.59 | -4.96 | 75.33 |
| TGFBR3 | -5.73 | -3.93 | 68.64 | -6.60 | -2.67 | 40.43 |
| OLFML2A | -5.79 | -2.62 | 45.23 | -3.90 | -3.57 | 91.42 |
| BFSP1 | -5.79 | -3.38 | 58.36 | -6.44 | -4.07 | 63.11 |
| TNFRSF14 | -5.87 | -3.09 | 52.59 | -5.74 | -4.66 | 81.28 |
| TMEM158 | -5.89 | -2.68 | 45.51 | -4.39 | -1.74 | 39.61 |
| ATOH8 | -5.90 | -3.24 | 54.88 | -7.66 | -3.94 | 51.37 |
| KRT19 | -5.90 | -2.84 | 48.19 | -5.56 | -4.06 | 73.05 |
| PHLDA1 | -5.90 | -3.04 | 51.44 | -3.49 | -3.42 | 98.20 |
| VWCE | -5.97 | -3.40 | 56.94 | -7.67 | -4.76 | 62.14 |
| RASL12 | -5.98 | -4.51 | 75.36 | -5.87 | -3.52 | 60.02 |
| AKAP12 | -6.18 | -1.42 | 22.99 | -3.37 | -1.96 | 58.10 |
| SOCS2 | -6.19 | -2.76 | 44.60 | -5.64 | -4.40 | 78.05 |
| GRB14 | -6.21 | -5.42 | 87.40 | -2.19 | -2.81 | 128.18 |
| SELENBP1 | -6.21 | -4.22 | 67.89 | -6.70 | -7.19 | 107.26 |
| CLEC3B | -6.22 | -3.02 | 48.59 | -4.66 | -3.14 | 67.35 |
| C9orf47 | -6.26 | -4.64 | 74.08 | -7.38 | -3.87 | 52.47 |
| FBLN1 | -6.28 | -2.21 | 35.22 | -6.27 | -2.78 | 44.40 |
| RTP4 | -6.31 | -2.80 | 44.46 | -5.41 | -3.99 | 73.74 |
| HGF | -6.33 | -2.83 | 44.81 | -5.75 | -2.63 | 45.73 |
| IFIT1 | -6.33 | -2.95 | 46.61 | -4.59 | -4.22 | 91.88 |
| DNM3 | -6.34 | -4.62 | 72.79 | -9.25 | -6.25 | 67.59 |
| LRRC20 | -6.35 | -3.30 | 51.96 | -4.89 | -4.85 | 99.06 |
| CARD16 | -6.42 | -2.13 | 33.25 | -4.99 | -2.57 | 51.55 |
| NR0B1 | -6.43 | -5.57 | 86.75 | -7.50 | -6.70 | 89.33 |
| FGF13 | -6.45 | -2.89 | 44.80 | -5.45 | -4.36 | 80.05 |
| PCOTH | -6.45 | -2.93 | 45.36 | -5.42 | -4.21 | 77.65 |
| FBLN1 | -6.46 | -1.60 | 24.81 | -6.00 | -3.28 | 54.67 |
| DCLK1 | -6.48 | -4.17 | 64.30 | -4.27 | -3.11 | 72.83 |
| GUCY1A1 | -6.50 | -6.04 | 92.95 | -7.81 | -5.62 | 71.88 |
| MME | -6.51 | -3.18 | 48.87 | -6.44 | -2.92 | 45.38 |
| P2RY1 | -6.53 | -3.04 | 46.53 | -9.48 | -3.94 | 41.54 |
| PCOTH | -6.55 | -2.86 | 43.66 | -6.46 | -4.49 | 69.53 |
| SAMD5 | -6.57 | -4.21 | 64.17 | -4.23 | -4.19 | 99.06 |
| CCDC102B | -6.62 | -5.38 | 81.25 | -8.12 | -4.64 | 57.07 |
| NEDD4L | -6.63 | -3.17 | 47.87 | -5.48 | -4.03 | 73.43 |
| CARD17 | -6.66 | -1.94 | 29.06 | -4.85 | -2.43 | 50.03 |
| GRK5 | -6.69 | -2.98 | 44.54 | -7.72 | -4.68 | 60.64 |
| CASP17P | -6.70 | -2.87 | 42.85 | -7.03 | -3.27 | 46.48 |
| AQP3 | -6.74 | -6.01 | 89.20 | -8.67 | -5.31 | 61.27 |
| MAOA | -6.78 | -2.07 | 30.55 | -5.42 | -3.85 | 71.03 |
| SPRY1 | -6.91 | -1.58 | 22.82 | -4.30 | -3.21 | 74.72 |
| APOC1 | -6.92 | -2.99 | 43.27 | -6.21 | -4.53 | 73.00 |
| ENST00000528781 | -6.93 | -1.85 | 26.68 | -3.54 | -3.64 | 103.00 |
| PDE5A | -6.93 | -3.66 | 52.89 | -4.62 | -3.10 | 66.99 |
| ZFP36L2 | -6.98 | -3.17 | 45.47 | -7.28 | -3.66 | 50.31 |
| COLEC12 | -6.99 | -3.89 | 55.56 | -8.25 | -2.94 | 35.62 |
| EPAS1 | -7.16 | -2.32 | 32.45 | -5.62 | -4.00 | 71.16 |
| KIT | -7.16 | -1.97 | 27.59 | -2.52 | -1.85 | 73.62 |
| ACVRL1 | -7.19 | -3.04 | 42.34 | -2.81 | -3.66 | 130.36 |
| ATP1A2 | -7.19 | -3.34 | 46.49 | -4.31 | -3.47 | 80.47 |
| OLFML1 | -7.22 | -2.50 | 34.64 | -4.19 | -4.08 | 97.40 |
| PTGIR | -7.36 | -2.04 | 27.67 | -3.42 | -1.99 | 58.29 |
| RASL11A | -7.38 | -5.73 | 77.66 | -8.14 | -4.37 | 53.63 |
| ALDH1A3 | -7.41 | -4.40 | 59.41 | -13.50 | -3.88 | 28.72 |
| CEMIP | -7.45 | -1.39 | 18.60 | -8.54 | -1.57 | 18.38 |
| CCBE1 | -7.54 | -2.23 | 29.61 | -7.22 | -5.14 | 71.18 |
| APCDD1 | -7.54 | -3.10 | 41.10 | -6.08 | -5.28 | 86.79 |
| FAXDC2 | -7.60 | -4.31 | 56.66 | -5.99 | -5.37 | 89.54 |
| PALMD | -7.66 | -3.42 | 44.59 | -6.85 | -4.87 | 71.03 |
| CCL2 | -7.76 | -0.92 | 11.87 | -5.95 | -2.18 | 36.54 |
| MYOC | -7.76 | -3.52 | 45.42 | -6.19 | -6.67 | 107.71 |
| LAMA4 | -7.79 | -2.82 | 36.16 | -7.22 | -4.82 | 66.76 |
| KITLG | -7.82 | -3.00 | 38.33 | -6.48 | -3.00 | 46.25 |
| ST8SIA1 | -7.90 | -4.59 | 58.15 | -8.26 | -6.09 | 73.65 |
| SVEP1 | -7.94 | -1.43 | 17.96 | -4.47 | -2.46 | 55.03 |
| LINC00484 | -8.08 | -6.04 | 74.73 | -7.21 | -6.41 | 88.90 |
| MME | -8.18 | -4.33 | 52.91 | -8.03 | -3.63 | 45.21 |
| NTN1 | -8.25 | -2.89 | 35.04 | -7.61 | -4.01 | 52.62 |
| RIPOR3 | -8.27 | -3.65 | 44.22 | -4.27 | -4.61 | 108.12 |
| CXCL12 | -8.30 | -1.73 | 20.79 | -9.20 | -4.16 | 45.17 |
| SLC9A9 | -8.36 | -5.47 | 65.38 | -7.32 | -6.36 | 86.86 |
| AMPD3 | -8.40 | -3.35 | 39.85 | -5.54 | -3.45 | 62.31 |
| IMPA2 | -8.42 | -2.26 | 26.85 | -4.51 | -4.28 | 94.94 |
| MOXD1 | -8.44 | -1.95 | 23.10 | -6.71 | -3.27 | 48.77 |
| GDF5 | -8.57 | -1.35 | 15.78 | -3.66 | -2.91 | 79.54 |
| IFITM1 | -8.78 | -1.82 | 20.73 | -5.83 | -1.87 | 32.00 |
| TBX2-AS1 | -8.81 | -3.97 | 45.11 | -6.90 | -4.97 | 72.11 |
| TCF21 | -8.91 | -5.15 | 57.80 | -9.79 | -8.82 | 90.08 |
| QPRT | -8.92 | -4.17 | 46.70 | -6.73 | -5.79 | 86.08 |
| APOL6 | -8.92 | -5.06 | 56.74 | -2.80 | -2.10 | 75.21 |
| TBX2-AS1 | -9.05 | -4.02 | 44.47 | -7.14 | -5.01 | 70.23 |
| DENND2A | -9.13 | -3.82 | 41.86 | -7.17 | -3.93 | 54.72 |
| METTL7A | -9.18 | -3.55 | 38.69 | -6.60 | -4.68 | 70.80 |
| GALNT15 | -9.26 | -3.40 | 36.75 | -4.83 | -3.49 | 72.13 |
| HSD11B1 | -9.30 | -2.82 | 30.29 | -10.23 | -3.99 | 39.01 |
| ACPP | -9.33 | -1.90 | 20.38 | -5.52 | -3.58 | 64.88 |
| TMTC1 | -9.33 | -2.35 | 25.22 | -5.34 | -2.93 | 54.86 |
| HTR2B | -9.40 | -2.01 | 21.39 | -3.84 | -1.83 | 47.53 |
| SFRP1 | -9.43 | -3.25 | 34.43 | -8.06 | -4.34 | 53.91 |
| CASP17P | -9.45 | -2.81 | 29.74 | -8.41 | -3.18 | 37.81 |
| MME | -9.47 | -3.41 | 35.96 | -7.84 | -3.15 | 40.17 |
| SMAD3 | -9.49 | -2.70 | 28.42 | -6.05 | -3.58 | 59.07 |
| CCN3 | -9.54 | -2.62 | 27.43 | -5.19 | -3.99 | 76.86 |
| HSPB3 | -9.64 | -2.38 | 24.71 | -6.40 | -2.73 | 42.67 |
| NR4A3 | -9.72 | -4.45 | 45.80 | -12.57 | -4.47 | 35.60 |
| IFITM1 | -9.92 | -1.83 | 18.46 | -6.86 | -2.26 | 32.95 |
| KRT32 | -9.95 | -2.87 | 28.85 | -6.73 | -4.87 | 72.34 |
| RGCC | -10.21 | -3.69 | 36.11 | -4.65 | -3.51 | 75.54 |
| PLEKHA6 | -10.29 | -3.88 | 37.76 | -9.24 | -5.01 | 54.26 |
| KCNJ2 | -10.32 | -3.87 | 37.56 | -8.59 | -3.64 | 42.41 |
| LCE2A | -10.41 | -6.14 | 59.00 | -28.27 | -4.48 | 15.86 |
| KRTAP1-5 | -10.49 | -1.89 | 18.01 | -6.80 | -1.89 | 27.74 |
| LRRN4CL | -10.59 | -2.68 | 25.34 | -7.05 | -6.06 | 85.94 |
| SPRY1 | -10.70 | -2.18 | 20.36 | -7.26 | -4.41 | 60.70 |
| MAN1C1 | -11.00 | -3.05 | 27.70 | -7.78 | -4.15 | 53.37 |
| S1PR3 | -11.17 | -3.86 | 34.59 | -9.45 | -5.93 | 62.74 |
| ANGPTL4 | -11.89 | -1.50 | 12.66 | -4.28 | -1.97 | 46.09 |
| GPX3 | -12.16 | -2.61 | 21.49 | -11.57 | -4.50 | 38.94 |
| LOC102724458 | -12.55 | -2.45 | 19.54 | -10.74 | -3.85 | 35.88 |
| PPL | -12.61 | -1.80 | 14.31 | -5.67 | -6.58 | 116.04 |
| ASPA | -12.67 | -4.76 | 37.58 | -10.56 | -5.80 | 54.96 |
| SYNE3 | -12.95 | -4.16 | 32.16 | -8.17 | -7.23 | 88.46 |
| PLIN4 | -13.00 | -3.45 | 26.56 | -16.30 | -9.20 | 56.45 |
| LSP1 | -13.52 | -5.12 | 37.88 | -7.28 | -6.20 | 85.13 |
| GPX3 | -13.70 | -2.68 | 19.53 | -10.27 | -4.92 | 47.91 |
| SHISA3 | -13.89 | -8.04 | 57.92 | -16.90 | -9.13 | 54.00 |
| LINC02154 | -14.07 | -3.45 | 24.50 | -7.49 | -4.86 | 64.90 |
| VSIR | -14.20 | -4.25 | 29.90 | -7.67 | -8.84 | 115.30 |
| FGL2 | -14.56 | -6.37 | 43.74 | -17.82 | -2.93 | 16.46 |
| PLPP3 | -14.66 | -4.75 | 32.41 | -11.48 | -5.58 | 48.62 |
| TMEM119 | -15.06 | -3.45 | 22.94 | -16.32 | -7.36 | 45.11 |
| SLC40A1 | -15.81 | -9.40 | 59.48 | -13.05 | -8.32 | 63.75 |
| RARRES3 | -16.76 | -4.10 | 24.49 | -10.44 | -5.07 | 48.56 |
| ADAMTS8 | -16.97 | -0.97 | 5.70 | -5.00 | -2.00 | 39.90 |
| A2M | -17.72 | -2.23 | 12.59 | -12.52 | -6.56 | 52.42 |
| SECTM1 | -18.30 | -4.51 | 24.67 | -18.30 | -6.98 | 38.11 |
| ALDH1A1 | -18.45 | -2.65 | 14.35 | -11.89 | -3.01 | 25.35 |
| SELENOP | -19.14 | -5.48 | 28.64 | -14.91 | -5.55 | 37.19 |
| TSGA10IP | -19.38 | -6.73 | 34.75 | -14.33 | -14.35 | 100.10 |
| SELENOP | -19.41 | -6.60 | 34.01 | -16.79 | -5.84 | 34.78 |
| ADH1B | -20.05 | -7.89 | 39.34 | -25.55 | -15.60 | 61.06 |
| TNXB | -20.13 | -3.28 | 16.28 | -20.56 | -5.35 | 26.03 |
| TNXB | -21.44 | -2.68 | 12.49 | -21.07 | -6.22 | 29.51 |
| SOCS1 | -24.81 | -10.60 | 42.71 | -18.63 | -12.67 | 68.01 |
| CLDN11 | -26.38 | -3.48 | 13.17 | -18.07 | -11.83 | 65.50 |
| FOXQ1 | -27.96 | -9.44 | 33.78 | -17.14 | -12.11 | 70.65 |
| NPTX1 | -34.26 | -16.29 | 47.56 | -36.43 | -14.79 | 40.61 |
| FMO2 | -42.81 | -7.63 | 17.82 | -27.39 | -3.88 | 14.17 |
| ENST00000584094 | -43.29 | -8.25 | 19.06 | -35.74 | -10.24 | 28.64 |
| ADM | -60.64 | -3.17 | 5.22 | -19.16 | -6.63 | 34.60 |
| ADH1A | -163.06 | -7.29 | 4.47 | -73.21 | -21.03 | 28.73 |
| ADH1C | -246.54 | -7.06 | 2.86 | -96.87 | -21.31 | 22.00 |
