# Supplementary material for: Identification of Paired-related Homeobox Protein 1 as a key mesenchymal transcription factor in pulmonary fibrosis
Source: eLife. 2023 Jun 1;12:e79840. doi: 10.7554/eLife.79840 (PMC10275639; doi:10.7554/eLife.79840)
Supplement: Supplementary file 3. [file elife-79840-supp3.docx]

**Supplementary Table S3: PCR primer sequences**

|  |  |  |
| --- | --- | --- |
| **Gene** | ***Forward*** | ***Reverse*** |
| *hUBC* | GTGGTGCGTCCAGAGAGAC | GGCCTTCGCCATATCCTTTTC |
| *hPRRX1a* | AGCGTCTCCGTACAGATCCT | GTAGCCATGGCGCTTTTCAG |
| *hPRRX1b* | TCCGAGACCCACCGATTATCT | AAGTAGCCATGGCGCTGTA |
| *hACTA2* | GAAGAGCATCCCACCCTGC | ATTTTCTCCCGGTTGGCCT |
| *hCOL1A1* | GCCAAGACGAAGACATCCCA | GTTTCCACACGTCTCGGTCA |
| *hFN1* | AGCAAGCCCGGTTGTTATGA | CCCACTCGGTAAGTGTTCCC |
| *hCCNA2* | CATGTCACCGTTCCTCCTTG | CCAATGGTTTTCTGGGTCCA |
| *hCCNE2* | TGGCCACCTGTATTATCTGGG | TCCCCAGCTTAAATCAGGCA |
| *hACTG2* | ATGTACGTCGCCATTCAAGC | TCTCTCTCAGCTGTGGTCAC |
| *hTAGLN* | GTATGACGAGGAGCTGGAGG | TCAGGGTACAGGCTGTTCAC |
| *hPPM1A* | TGCATGTGATGGTATCTGGGA | GCTTCTGGCGATACTTTGGG |
| *hTGFBR2* | ATGCTGCTTCTCCAAAGTGC | GCTGATGCCTGTCACTTGAA |
| *mRna18S* | CTTAGAGGGACAAGTGGCG | ACGCTGAGCCAGTCAGTGTA |
| *mPrrx1a* | CTCTCCGTACAGCGCCAT | GTTGGCCATGTTGATACCCT |
| *mPrrx1b* | CCGTACAGATCTTCGTCCCT | TTCCTCAGTTGACTGTTGGC |
| *mActa2* | AGTCGCTGTCAGGAACCCTGAGA | ATTGTCGCACACCAGGGCTGTG |
| *mCol1a1* | GTGTGTGACAAGGGTGAGACA | GAGAACCAGGAGAACCAGGA |
| *mFn1* | TGGTGGCCACTAAATACGAA | GGAGGGCTAACATTCTCCAG |
| *mCol14a1* | GTTCAACGTGGGCTCAGAAA | ACTCCTCGATCCTGCTTCTG |
| *mMki67* | AACAAGAGTGAGGGAATGCC | GCTGTGAGTGCCAAGAGACT |
| *hCCNA2* promoter | TTTAACACGGAGCTCACATAGT | CAGTAGTTCAAGGTGCCATCTTA |
| *hCCNE2* promoter | CGTGACGCCGGCAAAATAAT | AGCGTTAGAAATGGCAGAAAGT |
| *hMKI67* promoter | CCGCAACATTAGCAAATCGATTT | CGTCACTTTTCCTTGGTGCT |
| *hPPM1A* promoter | TGCGAATGTGGTGTAGGTCA | CGCCGGGGATAGAATGACAA |
