# Supplementary material for: Identification of Paired-related Homeobox Protein 1 as a key mesenchymal transcription factor in pulmonary fibrosis
Source: eLife. 2023 Jun 1;12:e79840. doi: 10.7554/eLife.79840 (PMC10275639; doi:10.7554/eLife.79840)
Supplement: Supplementary file 4. [file elife-79840-supp4.docx]

**Supplementary Table S4: Antibody list**

| ***Antibody*** | ***Concentration*** | ***Application*** |
| --- | --- | --- |
| GAPDH | Mouse monoclonal, Covalab, Villeurbanne, France (00006513) | Western Blot |
| β-TUBULIN | Rabbit polyclonal, Abcam, Cambridge, USA (ab6046) | Western Blot |
| β-ACTIN | Mouse monoclonal clone AC-74 (A2228), Sigma, Saint-Louis, USA | Western Blot |
| PRRX1 | Mouse monoclonal, clone 1E2, Sigma, Saint-Louis, USA (SAB1412737 | Western Blot |
|  | Rabbit polyclonal, Sigma, Saint-Louis, USA **(HPA051084)** | Immunochemistry; immunofluorescence and chromatin Immunoprecipitation |
| COL-I | Goat polyclonal ,Southern Biotech, Birmingham, USA ( 1310-01) | Western Blot |
|  | Rabbit polyclonal, Abcam, Cambridge, UK (ab34710) | Immunochemistry |
| FN-1 | Rabbit polyclonal, Abcam, Cambridge, UK (ab2413) | Western Blot |
| ACTA2 | Mouse monoclonal, clone 1A4, Sigma, Saint-Louis, USA (A5228) | Western Blot and immunochemistry |
| Phospho(S423+S425) SMAD3 | Rabbit monoclonal, Abcam, Cambridge, USA (ab52903) | Western Blot |
| SMAD3 | Rabbit polyclonal, Abcam, Cambridge, UK (ab28379) | Western Blot |
| Phospho(S255)-SMAD2 | Rabbit monoclonal, Abcam, Cambridge, USA (ab188334) | Western Blot |
| SMAD2 | Rabbit monoclonal, Cell signalling Technology, Danves, USA (5339S) | Western Blot |
| TGFBR2 | Rabbit polyclonal, Cell signalling Technology, Danves, USA (79424) | Western Blot |
| PPM1A | Rabbit polyclonal, Sigma, Saint-Louis, USA (HPA029209) | Western Blot |
| KI67 | Rabbit monoclonal, clone RM227, Sigma, Saint-Louis, USA (SAB5600050) | Immunochemistry |
| Vimentin | Rabbit monoclonal, Abcam, Cambridge, USA (ab92547) | Immunochemistry |
| CD45 | Mouse Monoclonal, Dako **Agilent Technologies**, Les Ulis France (M0701) | Immunochemistry |
| PDGFR | Rabbit polyclonal, Abcam, Cambridge, UK (ab32570) | Immunochemistry |
